# LMT USV Toolbox, a Novel Methodological Approach to Place Mouse Ultrasonic Vocalizations in Their Behavioral Contexts—A Study in Female and Male C57BL/6J Mice and in *Shank3* Mutant Females

**DOI:** 10.3389/fnbeh.2021.735920

**Published:** 2021-10-13

**Authors:** Fabrice de Chaumont, Nathalie Lemière, Sabrina Coqueran, Thomas Bourgeron, Elodie Ey

**Affiliations:** Human Genetics and Cognitive Functions, Institut Pasteur, UMR 3571 CNRS, Université de Paris, Paris, France

**Keywords:** mouse, ultrasonic vocalization (USV), social behavior analysis, mouse model, autism, age, sex, *Shank3*

## Abstract

Ultrasonic vocalizations (USVs) are used as a phenotypic marker in mouse models of neuropsychiatric disorders. Nevertheless, current methodologies still require time-consuming manual input or sound recordings clean of any background noise. We developed a method to overcome these two restraints to boost knowledge on mouse USVs. The methods are freely available and the USV analysis runs online at https://usv.pasteur.cloud. As little is currently known about usage and structure of ultrasonic vocalizations during social interactions over the long-term and in unconstrained context, we investigated mouse spontaneous communication by coupling the analysis of USVs with automatic labeling of behaviors. We continuously recorded during 3 days undisturbed interactions of same-sex pairs of C57BL/6J sexually naive males and females at 5 weeks and 3 and 7 months of age. In same-sex interactions, we observed robust differences between males and females in the amount of USVs produced, in the acoustic structure and in the contexts of emission. The context-specific acoustic variations emerged with increasing age. The emission of USVs also reflected a high level of excitement during social interactions. We finally highlighted the importance of studying long-term spontaneous communication by investigating female mice lacking *Shank3*, a synaptic protein associated with autism. While the previous short-time constrained investigations could not detect USV emission abnormalities, our analysis revealed robust differences in the usage and structure of the USVs emitted by mutant mice compared to wild-type female pairs.

## Introduction

Social communication regulates major biological functions under strong selective pressure, such as finding reproductive partners, raising progeny, group coordination for territory advertisement, and protection from predators (Bradbury and Vehrencamp, 2011). It is not yet clear whether the mechanisms underlying these functions are shared between species, but an increasing number of genes and brain circuits related to social interaction and communication have been identified (e.g., Arriaga et al., 2012; Chen and Hong, 2018; Tu et al., 2019; Kelley et al., 2020), suggesting that at least certain social brain circuits are conserved across species. Several genes associated with autism have been mutated in animal models and lead to atypical social interactions (e.g., mice: Ey et al., 2011; Crawley, 2012; rats: Modi et al., 2018; Drosophila: Coll-Tané et al., 2019; monkeys: Tu et al., 2019). This suggests that animals can be used as models to better understand the causes of neuropsychiatric conditions affecting social interactions and communication, keeping in mind their limits (e.g., the absence of convincing proofs of vocal learning, a major characteristic of human communication, in the above-cited model species; Jarvis, 2019). Hereafter, we will focus on mice, due to their broad use as models for neuropsychiatric disorders.

Mice are social animals, naturally living in *demes*, with a single dominant male, occasionally a few subordinate males, and several females occupying contiguous nests, but only a fraction of them reproduce (Palanza et al., 2005). This social organization leads mice to use tactile, olfactory, visual, and vocal (mostly in the ultrasonic range) signals in same-sex social interactions, male-female socio-sexual interactions, and mother-infant relationships (Latham and Mason, 2004; Brennan and Kendrick, 2006; Portfors, 2007). Tactile, visual, and olfactory cues are investigated through the observation of contacts, body postures, and marking behavior (e.g., Mathis et al., 2018; de Chaumont et al., 2019). Ultrasonic vocalizations (USVs) are interpreted as a proxy for vocal communication (Zippelius and Schleidt, 1956; Sewell, 1970; Portfors, 2007). USVs may also represent emotional reflectors of social interactions, reflecting the positive or negative valence of the interaction and the excitement of the interacting mice. Our understanding of mouse USVs nevertheless is still poor compared to our knowledge of vocal communication in other species, such as birds or primates (reviewed in Naguib et al., 2009; for non-human primates in Fischer and Hammerschmidt, 2010).

Mouse USVs are generally investigated in contexts in which the motivation of the animals is controlled, through either social deprivation or the introduction of a sexual component to trigger a maximum quantity of USVs (reviewed in Portfors, 2007). Indeed, after the developmental period when isolated mouse pups emit USVs that trigger approach and retrieval from the mother (Zippelius and Schleidt, 1956; Sewell, 1970), the amount of USVs emitted by juveniles and adults is maximized by social deprivation (Ey et al., 2018). In these contexts, USVs may be used to regulate close contacts and hierarchy (Moles et al., 2007; Ey et al., 2018). Finally, an estrous female, or at least its odor, stimulates adult males to vocalize (Holy and Guo, 2005; Chabout et al., 2015). Such male USVs are suspected to facilitate close body contact with the female (Pomerantz et al., 1983; Hammerschmidt et al., 2009; reviewed in Egnor and Seagraves (2016). Examining USVs in these specific contexts allowed to suggest some of their functions, but, to refine them, linking USVs emission to behavioral contexts is required.

Under constrained conditions, previous studies highlighted that USVs are mostly emitted during close contacts and approach behaviors in male-male (Ferhat et al., 2015) and female-female (Ferhat et al., 2016) interactions (including when one is socially deprived). Specific phases of courtship interactions trigger different call types, which might therefore reflect the different behaviors expressed in these phases (Nyby, 1983; Hanson and Hurley, 2012). It was also shown that male-female and female-female interactions trigger the longest USVs sequences, with the most complex types of USVs, compared to male-male interactions (Matsumoto and Okanoya, 2021). In a group of four adult mice (two males and two females, aged 3–5 months) interacting for 5 h after at least 2 weeks of social deprivation, both the females and males vocalized, more specifically during chasing (Neunuebel et al., 2015). The lowest call rates occurred when the animals were isolated from one another, whereas the highest call rates occurred when the mice were sniffing each other’s ano-genital region. Vocalizations are therefore emitted in different proportions depending on the behavioral context (Sangiamo et al., 2020). In paired interactions, USVs trigger no behavioral variations in female-female interactions, but males run faster when the females accelerate while vocalizing in male-female interactions (Warren et al., 2018a,b, 2020). Altogether, linking USVs with behavioral contexts remains focused on constrained interactions and does not shed light on the usage of spontaneous USVs.

**Figure 1:**
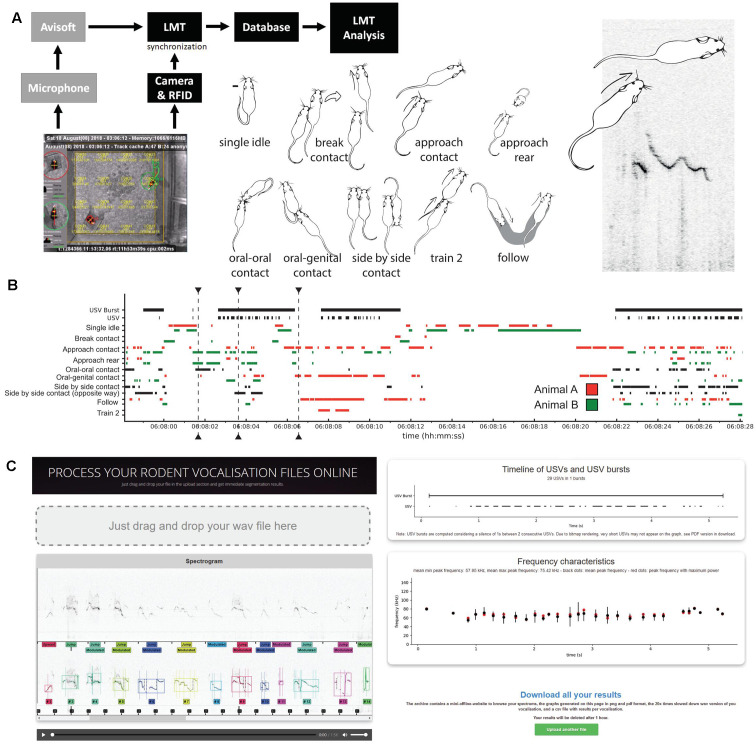
Overview of the study of spontaneous ultrasonic vocalizations (USVs) in same-sex pairs of mice. **(A)** Set-up used to synchronize the spontaneous USVs and behaviors of a same-sex pair of mice using AviSoft and Live Mouse Tracker (LMT). Examples of behaviors automatically extracted using LMT; see definitions in “Materials and Methods” Section and in de Chaumont et al. (2019). **(B)** Timeline of 30 s of the experiment (day 1, 6 a.m.), with USVs, USV bursts, and the major behavioral events. **(C)** Screenshots of the online processing application (https://usv.pasteur.cloud) with an example set. Spectrogram and automatically extracted USVs in which each color labels a different USV. The segmentation follows the shape of the frequency of maximum amplitude. USV and USV burst timeline extracted, frequency characteristics. “Download all results” button providing figures and data for each acoustic feature extracted for each voc.

In the present study, we developed a method using the Live Mouse Tracker (LMT; de Chaumont et al., 2019), which allows a detailed description of the behavior, synchronized with USV recordings (Figures 1A,B). We delivered the complete pipeline of recording and analyses, as well as an original online application^1^ (Figure 1C) to automatically segment and analyze the USVs. Using this method, we characterized the spontaneous social and vocal behavior of C57BL/6J (hereafter B6) mice in same-sex pairs over an undisturbed period of 3 days and nights. We tested whether the usage and structure of USVs vary according to their behavioral contexts of emission. We focused on same-sex interactions between sexually naive mice to avoid mixing sexual motivation in the factors that influenced vocal behavior. We compared the vocal behavior of male and female pairs at three different ages (5 weeks and 3 and 7 months) to examine age-related maturation. In addition, we investigated whether spontaneous USVs are perturbed in mice lacking Shank3, a glutamatergic synaptic scaffolding protein that we previously associated with autism (Durand et al., 2007; Leblond et al., 2014; deletion of exon 11; Schmeisser et al., 2012; Vicidomini et al., 2017). This first in-depth investigation of spontaneous mouse social communication and the resources presented here will be helpful for the community to design more sensitive tests to better investigate the natural abilities of mice (Warburton et al., 1988; Gerlai and Clayton, 1999), as well as to detect and interpret social communication phenotypes in mouse models of neuropsychiatric disorders.

## Materials and Methods

### Animals

We tested eight male and eight female C57BL/6J mice (hereafter B6 mice; Charles River Laboratories, Ecully, France). Mice arrived at 3 weeks of age as a group and were directly housed in same-sex pairs in the experimental facility. *ProSAP2/Shank3* mutant mice (deletion of exon 11; Schmeisser et al., 2012) were generated onsite from heterozygous parents on a C57BL/6J background (>10 backcrosses). Twelve *Shank3*^−/−^ females arrived at 1–1.5 month of age in the experimental facility. Before arrival, these mice were housed within mixed-genotype groups, littermates or not. *Shank3^+/+^* females were not available in sufficient number to conduct a similar study with these control mice, and therefore B6 females aged 3 months were used as control group given their similar genetic background. *Shank3*^−/−^ females were directly housed in age-matched pairs upon arrival, at least 3 weeks before the recording session to let them stabilize social relationships. In this mouse model, we focused on a single age class (3 months of age) since it is the classical age for behavioral characterization. We focused only on females given the low amount of USVs spontaneously emitted by B6 males, which is the genetic background used to generate the *Shank3* mutant mice tested here. In the experimental facility, mice were housed in classical laboratory cages with food and water *ad libitum* and 11 h/13 h dark/light rhythm (lights off at 08:00 p.m.).

We inserted RFID chips (12 × 2.12 mm; Biolog-Id, Bernay, France) subcutaneously behind the left ear and pushed it down to the flank at 4 weeks of age in B6 mice and between 5 and 7 weeks in *Shank3*^−/−^ mice. After this operation conducted under gas (isoflurane) anesthesia and local analgesia (<0.05 ml lidocaïne at 20 mg/ml), mice were left at least 1 week to recover.

We did not control for the sexual status of females in our experiments. Nevertheless, as the experiments lasted 3 days, we cover a large proportion of the sexual cycle (see also von Merten et al., 2014). In addition, we wanted to avoid manipulating animals throughout the recording session and following the estrus cycle would have necessitated daily handling.

### Behavioral and USV Recordings

For the recordings, each pair was placed in the LMT setup (de Chaumont et al., 2019), a Plexiglas cage of 50 × 50 cm with transparent walls furnished with fresh bedding, food dispersed on the ground in the center, a water bottle at the down right side of the cage, a house (width: 100 mm, depth: 75 mm, height: 40 mm) in red Plexiglas in the down left corner and nesting material (six dental cottons) spread on the bedding in the center of the cage. Each LMT setup was isolated in an experimental room, with no other mouse in the same room. Recordings were launched between 03:00 and 04:00 p.m. (20–23°C, 80–100 lux when lights were on). Once the recordings were started, we did not disturb the mice anymore for 3 days (11 h/13 h dark/light rhythm, with lights off at 08:00 p.m.). Recordings were stopped after 71 h and setups were cleaned with soap water, dried, and re-furnished before launching another recording session. In WT mice, the first recordings occurred at 5 weeks of age. We next recorded in identical conditions the same mice at 3 and 7 months of age. Between the recording sessions, the pairs of mice were left undisturbed, with only a weekly change of the bedding. The six pairs of *Shank3*^−/−^ mice were recorded only once, at 2.5–3 months of age, the age class classically recorded in behavioral phenotyping of mouse models.

Launching recordings consisted in monitoring both behavior and USV emission. Behavioral monitoring occurred through the LMT system (Kinect camera on the top, plugins 465, 524, 600, or 705; de Chaumont et al., 2019), in which mice were automatically identified and tracked throughout the recording session. All spontaneous ultrasonic vocalization sequences were recorded using an Avisoft-Bioacoustics CM16/CMPA microphone (located 50 cm above the bottom right corner of the LMT cage, oriented toward the center of the cage) and the Avisoft UltraSoundGate Recorder system (Avisoft Bioacoustics, Glienicke, Germany; 300 kHz sampling rate, 16-bit format) using the trigger function (trigger: level of this channel; pre-trigger: 1 s; hold time: 1 s; duration >0.005 s; trigger event: 2% energy in 25–125 kHz with entropy <50%). Both systems were synchronized within LMT (see hereafter). Behaviors were attributed to individuals while USVs were measured at the scale of the pair of mice since we cannot identify the emitter.

### Behavioral Events

The spontaneous behavior of the mice was automatically labeled using the LMT system. Both social and non-social behaviors were used, as in (de Chaumont et al., 2019; recapitulated in Table 1).

**Table 1:** Definitions of behavioral events extracted by Live Mouse Tracker.

Behavioral events	
Name	Description
**Single idle**	The mouse is stopped (speed lower than 5 pixels per frame) and not in contact with any other mouse.
**Single move**	The mouse is moving (speed higher than 5 pixels per frame) and not in contact with any other mouse.
**Break contact**	The mouse breaks a contact with another mouse and moves away from this mouse.
**Social approach**	The mouse is approaching another one, i.e., the distance between the two animals shortens, the speed of the mouse is higher than the speed of the approached mouse, and the distance between the two animals is shorter than two mean body lengths (of the approached animal). This approach does not necessarily lead to a contact.
**Approach contact**	The mouse is approaching another one and makes contact with it.
**Contact**	The mouse is in contact (i.e., the two masks have one common pixel) with the other mouse.
**Oral-oral contact**	The mouse is sniffing the oral region of another mouse, i.e., the two nose points are less than 15 pixels (26 mm) from one another.
**Oral-genital contact**	The mouse is sniffing the ano-genital region of another mouse, i.e., the nose point of the first mouse is within 15 pixels (26 mm) from the tail point of the other mouse.
**Side by side contact**	The side of a mouse is within 30 pixels (52 mm) from the side of the other mouse; both animals are oriented in the same direction.
**Side by side contact (head-to-tail)**	The side of a mouse is within 30 pixels (52 mm) from the side of the other mouse; both animals are oriented in opposite directions.
**Follow**	The mouse is walking alone directly in the path of another mouse. Both mice move at a speed higher than 5 pixels per frame, with an angle between their direction vectors smaller than 45°, and the distance between their centers of mass shorter than two mean body lengths.
**Train2**	The mouse is following another mouse (speed higher than 5 pixels per frame) while sniffing its ano-genital region.

### USV Segmentation Method

#### Vocalization Waveform to Spectrum

We process wav files recorded at a sampling rate of 300 kHz with a resolution of 16 bits per sample. We first apply an FFT (overlap of 0.75% and FFT-size = 1,024 points) to the original audio signal to get its spectrum.

The signal recorded presents three main problems: (1) The continuity of the USVs can be interrupted if the signal gets too low. (2) The animals produce a lot of noise by interacting with their environment. (3) The animal facility environment itself produces interfering ultrasonic noise. To overcome these problems which might prevent correct classification, we need to filter the signal of the spectrum. The processing steps are detailed in the Supplementary Methods section (Supplementary Methods—USV segmentation method: Method parameters, Filtering spectrum data, Constant and blinking frequency canceler, Vocalization segmentation, Spectrum signal extraction). The USV segmentation was validated on a set of manually annotated USVs (Supplementary Methods—USV detection validation; Supplementary Table I). As recorded files might contain USVs or noise, we sorted them automatically (Supplementary Methods—Filtering out wave files containing only noise) and double-check them manually.

#### Acoustic Feature Extracted

The acoustic traits were computed for each USV (Supplementary Table II) and each USV burst (Supplementary Table III).

#### Avisoft Burst Record and Synchronization With Live Mouse Tracker

The system is designed to work for an unlimited duration. As USVs are infrequent events, we do not record the sound continuously. We instead use the automatic record trigger functionality of Avisoft-RECORDER. The automatic trigger of Avisoft-RECORDER monitors the sound level and starts recording a sound if the current sound level within a given frequency range is over a given threshold. The sound is recorded as long as the sound level is over the threshold. This function takes a hold time parameter: if Avisoft-RECORDER detects another signal during the hold period, the record is not interrupted. The hold period also adds a record period around the first and the last signal over the threshold. In our experiment, we use a hold time of 1 s.

To synchronize USV recording with the tracking, we use the “Trigger control” of Avisoft-RECORDER. This function allows launching of an external program at each start and end of records. We use the free software PacketSender^2^ to perform communication between Avisoft-RECORDER and Live Mouse Tracker (LMT). Through PacketSender, we send a UDP string packet containing the file number currently recorded by Avisoft-RECORDER. This information is recorded by LMT within the database as an “USV event.” The goal of the synchronization is to match the USV record with the current data frame number recorded by LMT.

### LMT USV Toolbox, an Open-Source, Free, and Online USV Analysis Pipeline

The currently available methods to detect and analyze mouse USVs need specific installations and software. To facilitate the testing of our own algorithm, we provide a website to test the method or to process data online^1^. The user simply drags and drops his/her wave file, waits a few seconds (depending on the length of the sample file), and finally evaluates the quality of the USV segmentation and the data extracted from the sound file. The goal of this website is to provide immediate access to the method without installing any software.

The first panel of the website is dedicated to evaluating USV detection. The first spectrogram represents the original data and the second one provides the annotated data. The player under this spectrogram allows to listen to the sound file slowed down by twenty times. The other panels display:

- the length of the wave file given as input.
- the number of USVs detected within the wave file.
- a timeline displaying the USVs detected over the whole file and their temporal organization in USV bursts, in which the intervals between USVs are shorter than 750 ms.
- the frequency characteristics of each USV within the sound file (in kHz). Each vertical black bar displays the min/max peak frequency of the USV (and therefore also the frequency range) while the black dot displays the mean peak frequency and the red dot displays the peak frequency with the maximum amplitude in each USV.
- the duration of each USV (in ms).
- the power (i.e., amplitude) of each USV, depicted in arbitrary unit.
- the proportion of USVs with frequency modulations.
- the proportion of USVs containing one or more frequency jump(s).
- a table gathering all the acoustic variables extracted on each USV of the sound file.

The user can download all these results for his/her own sound file. These results are deleted after 1 h. Data downloaded from this web page can be directly used with the scripts that we provide in the present study. To perform the analysis on thousands of files, we also provide the desktop version of the analysis program, working in batch mode (download through the Live Mouse Tracker website: http://livemousetracker.org).

### USV Analysis Toolbox

For Live Mouse Tracker, we provided a full API in Python to process event classification and to process queries. As for Live Mouse Tracker, we provide an API in Python for the biologists to process USVs^3^. This package allows one to re-create all data representations used in this study with its own data. This API is available on gitHub^3^.

### Analyses and Statistical Tests

The whole data processing, computations, statistical analyses, and figure generation were conducted using Python 3.8. *P*-values are presented as uncorrected for multiple testing, with a distinctive sign (°) if they survived after Bonferroni correction indicated hereafter for each analysis.

#### Behavioral Profiles of Mice

We built a behavioral profile for each mouse considering the following behavioral event types: single move, single idle, get away, break contact, approach within the social range, approach contact, nose-nose contact, nose-anogenital contact, side-by-side contact, side-by-side contact/head-to-tail, follow, train2. We also examined the total distance traveled. For each behavioral event type, we compared the total time spent in this behavioral event type, the number of events, and the mean duration of these events.

Each of these variables was tested for the effect of age within each sex separately. We used a non-parametric Kruskal–Wallis test followed by paired Wilcoxon tests to compare age classes within each sex. When applying a correction for multiple testing, we corrected it by the number of tests conducted (2). The effect of sex was tested within each age class separately using Mann–Whitney *U*-tests. When applying a correction for multiple testing, we corrected it by the number of age classes (3). Variables for *Shank3*^−/−^ mice were compared to variables for B6 mice aged of 3 months using Mann–Whitney *U*-tests (no correction applied).

#### Comparison Between Age Classes, Sexes, or Genotypes of USVs and USV Bursts

To examine the effect of age class, we used nonparametric paired Wilcoxon tests to compare the amount of USVs between age classes within each sex. When applying a correction for multiple testing, we corrected it by the number of age classes (3). We compared the amount of USVs between *Shank3*^−/−^ and B6 mice using nonparametric Mann–Whitney *U*-tests (no correction applied).

To detect an age or a sex effect, we used a linear mixed model (fixed factor: age or sex, random factor: pair) to compare the number of USVs per USV burst between sexes within each age class (5 weeks, 3 months, and 7 months). When applying a correction for multiple testing, we corrected it by the number of age classes (3). To detect an effect of the *Shank3* mutation on the number of USVs per USV burst, we used a linear mixed model (fixed factor: genotype, random factor: pair) to compare the number of USVs per USV burst between *Shank3*^−/−^ pairs and B6 pairs. The same analyses were conducted within each behavioral context (single idle, break contact, approach contact, nose-nose contact, nose-anogenital contact, side-by-side contact, side-by-side contact/head-to-tail, follow, train2). When applying a correction for multiple testing, we corrected it by the number of behavioral contexts examined (9).

Acoustic features of USVs were compared between sexes within age classes, between age classes within each sex, and also between sexes within age classes and within each behavioral context using Linear Mixed Models using the pairs as a random factor. When applying a correction for multiple testing, we corrected it by the number of age classes (3) multiplied by the number of acoustic variables tested (16) when testing sex effect, by two tests multiplied by the number of acoustic traits (16) when testing age effect. When comparing acoustic traits across contexts, corrections included the number of acoustic traits (16) and the number of contexts combinations. We also compared the acoustic features between B6 and *Shank3*^−/−^ mice using Linear Mixed Models with genotype as a fixed factor and genotype as a random factor. When applying a correction for multiple testing, we corrected it by the number of acoustic variables (16).

#### Context-Specific Acoustic Features

We first selected representative acoustic variables (Supplementary Methods—Selection of representative acoustic variables) for the main text but nevertheless depicted all of them in the Supplementary Figures. We tested whether acoustic features of USVs (duration, frequency characteristics, frequency range, modulations, harshness, slope) varied according to the contexts in which USVs were emitted. For that purpose, after computing the acoustic features of all USVs, we compared acoustic features of USVs between the different behavioral contexts (single idle, break contact, approach contact, nose-nose contact, nose-anogenital contact, side-by-side contact, side-by-side contact/head-to-tail, follow, train2) using linear mixed models (fixed factor: context, random factor: pair) with a Bonferroni correction (combination of two within nine behavioral events × 16 acoustic features). Acoustic variations between contexts in *Shank3*^−/−^ mice were tested similarly (Figure M1c-d). Heatmaps represented significance levels after Bonferroni correction (size of the points) as well as the effect size and direction of variations (cold/warm colors).

#### Relationship Between the Occurrence of USV Bursts and the Speed of Mice as Well as the Duration of Social Events

We focused on the following sample of behavioral events: break contact, approach contact, contact, nose-anogenital contact, follow, and Train2. First, we tested whether animals displaying these behaviors accompanied by USVs had a higher speed than when they displayed the same behaviors without USVs. For that purpose, for each behavioral event of each type, we computed the mean speed of the mouse displaying this behavior over the whole event. We performed a paired Wilcoxon test (alternative = “greater”) to compare the mean speed of the animal with and without USVs at the group level. In addition, to better understand inter-individual variations, we compared, within each individual and for each behavioral event type, the speeds during events overlapping with USVs to the speeds during events not overlapping with USVs using Mann–Whitney *U*-tests (unpaired, one-sided).

Second, we tested whether one type of behavioral event was longer when emitted concomitantly with USVs than when it was not concomitant with USVs. For that purpose, within each individual and for each behavioral event type, we gathered the durations of events overlapping with USVs as well as the duration of events not overlapping with USVs. We compared these two lists of durations using Mann–Whitney *U*-tests (unpaired, one-sided) within each individual. We also performed a paired Wilcoxon test (alternative = “greater”) to compare at the group level the mean duration of the event with USVs and the mean duration of the same event without USVs. To control for the fact that longer behavioral events were more likely to include USVs, we used a random process in which USVs would occur at a random time. In this case, the longer USVs would have more chances to overlap with a USV. We simulated 1,000 different random distributions of the USVs, and we kept the same proportions of USVs overlapping with behavioral events as in the original data. We performed this test for each behavioral event in each experiment separately (data not shown). In each simulation, we performed the same statistical test (Mann–Whitney *U*-test) as in the original data, and we kept the *p*-value. The ratio of the number of *p*-values below the original one provided the probability to obtain the same effect randomly. In our study, this probability was less than 3%, suggesting that the effects of the presence of USVs on the duration of behavioral events could be found randomly with only a weak probability, i.e., longer events were not more likely to occur with USVs than shorter ones.

***simulation code:***

***Compute_Duration_Events_With_USV_Simulation.py***

We conducted these tests for both animals of each WT female pair at 5 weeks, 3 months, and 7 months of age as well as for both animals of *Shank3*^−/−^ pairs aged of 3 months for all behavioral events above cited. Overall, we adjusted our statistical analyses for multiple testing accordingly (Bonferroni correction).

#### Intonation Within USV Bursts

The intonation graph displays a representation of the burst where the duration of each burst is stretched from 0 to 100. 0 for the start of the burst and 100 for its end. Corresponding frames are computed and if a USV is present at this frame, we report the value for the given acoustic parameter and display −/+ its STD. The same method is performed for the number of USVs.

***Corresponding code and graph generation:***

***HarshLMocationInBurst3.py***

## Results

### A Platform to Analyze and Synchronize USVs With Behaviors

We needed to automatically detect USVs despite the background noise (i.e., animals moving in the bedding, continuous electronic or ventilation noises) and to extract their acoustic features, all these aspects being synchronized with behavioral monitoring (Supplementary Methods—Motivation to create a new recording and analysis pipeline). In our system, sounds were recorded using Avisoft SASLab Pro Recorder and synchronized with behavioral monitoring using LMT (Methods—Avisoft record and synchronization with Live Mouse Tracker). Mechanical noises (e.g., amplitudes at continuous frequencies from electronic devices or ventilation) were removed (Supplementary Methods—Filtering spectrum data) and the USVs extracted (Supplementary Methods—USV segmentation method/USV detection validation). The USVs were then synchronized with behaviors and the acoustic characteristics extracted and stored as metadata in the database (Supplementary Tables II, III—Description of the acoustic variables measured for each USV and burst). We provide a library in Python to access and query these data. We developed an online application that allows the segmentation and analysis of wave files without software installation^1^. Annotated spectrograms are generated online to allow visual inspection of the results. We also provide online examples. The desktop version of this resource is also available and open source.

### Age- and Sex-Related Variations in Spontaneous Social Behaviors

**Figure 2:**
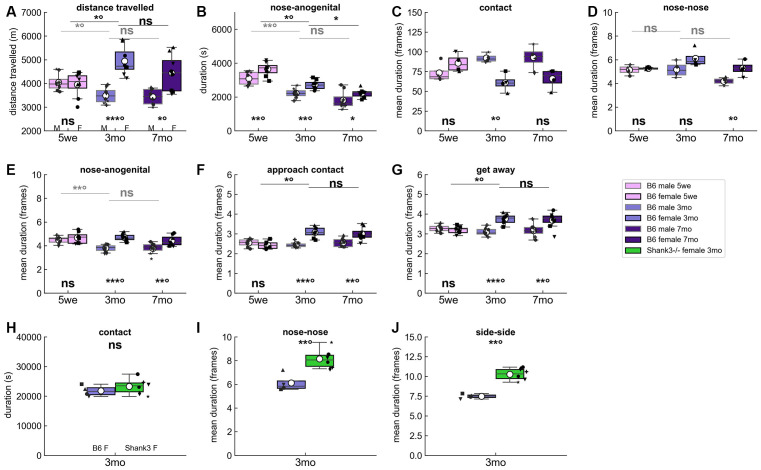
Spontaneous behaviors in B6 male and female pairs as well as in *Shank3*^−/−^ female pairs over three nights. **(A)** Total distance traveled over the three nights of recording in B6 male (M) and female (F) pairs at the three age classes tested. Age- and sex-related variations in the **(B)** total time spent in nose-anogenital contact, **(C)** mean duration of contact events (i.e., contacts in a broad sense without any specific body part involved), **(D)** mean duration of nose-nose contact events, **(E)** mean duration of nose-anogenital contact events, **(F)** mean duration of social approach events, and **(G)** mean duration of get away events over the three nights of recording. Each black dot corresponds to an individual in **(A,B,E–G)** with a similar shape for the two individuals of the pair, while in **(C,D)** each dot corresponds to a pair since the behaviors are symmetrical (Mann–Whitney *U*-tests used to test for differences between sexes within each age class; Kruskal–Wallis test followed by Wilcoxon paired test if significant to test for differences between 5 weeks and 3 months and between 3 months and 7 months within each sex). Comparisons between B6 female pairs aged 3 months and age-matched *Shank3*^−/−^ female pairs of the **(H)** total time spent in contact, **(I)** mean duration of nose-nose contact events, and **(J)** mean duration of side-side contact events over the three nights. Each black dot corresponds to a pair since the behaviors are symmetrical (Mann–Whitney *U*-tests used to test for differences between genotypes). Uncorrected *p*-values: ns: non significant, *p < 0.05, **p < 0.01, ***p < 0.001. *P*-values followed by ° survived a correction for multiple testing over age classes.

We examined the spontaneous social interactions of male and female same-sex pairs of B6 mice over 3 days. The previously observed reduced activity in males compared to females (de Chaumont et al., 2019) was not significant in juvenile males but only in mature males (i.e., aged 3 months or more) compared to females (Figure 2A). The reduced time spent in social contacts displayed by males compared to females was visible already in immature males (example of nose-anogenital contact in Figure 2B; see also Supplementary Figure 1). Surprisingly, the mean duration of non-specific contacts (i.e., contact between the two mice without specifying any body parts) tended to be longer in mature males than in females (Figure 2C), while, as soon as specific contact are considered, the mean duration of these specific contacts such as nose-nose (Figure 2D) and nose-anogenital contacts (Figure 2E and Supplementary Figure 2) were shorter in males than in females. Therefore, even if contact events are globally longer in males than in females, these two very specific contact types seem more prominent in females than in males. Males also displayed significantly shorter approach (Figure 2F) or escape (Figure 2G) behaviors compared to females in adulthood, suggesting that their contacts initiated and terminated more abruptly, possibly reflecting aggressive interactions. As expected, these sex-related differences were less visible when the animals were immature (i.e., aged 5 weeks; Supplementary Figures 1, 2). In both sexes, the total time spent in nose-nose and nose-anogenital contacts tended to decrease with increasing age (Figure 2B and Supplementary Figure 1).

Compared to age-matched pairs of wild-type B6 female mice, *Shank3^−/−^* pairs displayed atypical organization of their social contacts. Indeed, while they spent typical total time in social interactions (Figure 2H and Supplementary Figure 3), *Shank3*^−/−^ pairs displayed oral-oral (Figure 2I) and side-side (Figure 2J) contact events of significantly longer mean durations compared to age-matched B6 females (see also Supplementary Figure 4).

### Major Sex-Related Variations in the Usage of Spontaneous USVs

#### Quantification of USVs

**Figure 3:**
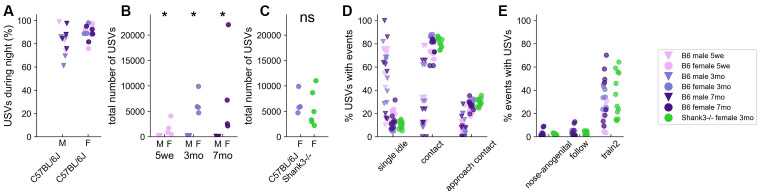
Spontaneous emission of USVs in B6 male and female pairs as well as in *Shank3*^−/−^ female pairs over three nights. **(A)** The proportion of USVs emitted during the night periods of the 3 days of recording in B6 pairs. **(B)** Total number of USVs recorded in male-male and female-female B6 pairs at 5 weeks, 3 months, and 7 months of age over the 71 h of recording (Mann–Whitney *U*-tests; uncorrected *p*-values: *p < 0.05; none of the *p*-values survived a correction for multiple testing. **(C)** Total number of USVs recorded in female-female B6 pairs and in female-female *Shank3*^−/−^ pairs aged 3 months (Mann–Whitney *U*-tests; ns: not significant). **(D)** The proportion of USVs occurring simultaneously with single idle and contact events in male-male and female-female B6 pairs at 5 weeks, 3 months, and 7 months of age (age classes represented by colors) as well as in female-female *Shank3*^−/−^ pairs aged 3 months. **(E)** The proportion of nose-anogenital contacts, follow and train2 behaviors occurring simultaneously with USVs in female-female B6 pairs at 5 weeks, 3 months, and 7 months of age (age classes represented by colors) as well as in female-female *Shank3*^−/−^ pairs aged 3 months.

Over these 3 days of monitoring, spontaneous USVs were mostly (around 80%) emitted during the nocturnal activity period in both B6 males and females of any age (Figure 3A and Supplementary Figures 5, 6). Interestingly, there was a strong sex-related difference in USV emission, with pairs of B6 males emitting significantly fewer USVs than pairs of B6 females across all age classes over the 3 days of recording (Figure 3B). The total number of USVs recorded for 3 months-old *Shank3*^−/−^ females over the 3 days (5,527.0 ± 3,236.8) was not statistically different from that of USVs recorded for age-matched B6 females (6,589.3 ± 1,979.8; Mann–Whitney *U*-test: *U* = 8.0, *p* = 0.228; Figure 3C). *Shank3*^−/−^ mice emitted significantly fewer USVs than B6 mice only during the second night (*U* = 2.0, *p* = 0.021; data not shown).

#### Contexts of Emission of Ultrasonic Vocalizations

We next examined the contexts in which USVs were emitted. Again, we observed a strong sex-related difference in the contexts of emission. Indeed, in males, most USVs were emitted when the mice were at a distance from each other (i.e., single idle), while in females most USVs were emitted in contact with each other, initiating contact, or approaching in a social range (Figure 3D). Interestingly, the Train2 behavior (i.e., following while keeping ano-genital contact) was highly specific to USV emission since up to 70% of these events were accompanied by USVs, in contrast to even closely related social behaviors such as ano-genital contacts or follow without contact (Figure 3E). The context-specificity described here was observed in all age classes as well as in 3-months old *Shank3*^−/−^ female pairs (Figures 3D,E), despite their perturbed social behavior (see behavioral profiles above).

### Variations of the Acoustic Characteristics of USVs With Sex, Age, and Genotype

**Figure 4:**
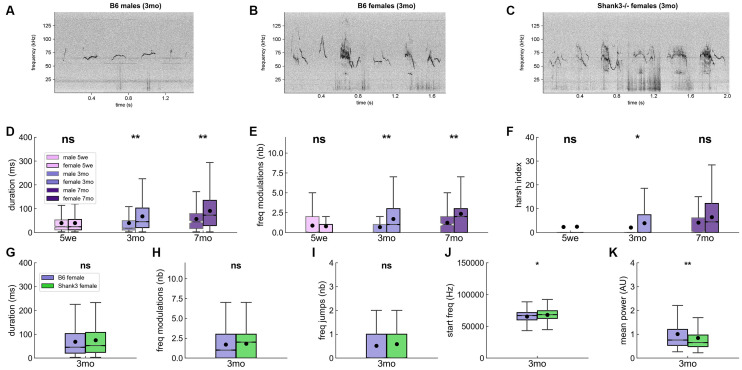
Acoustic characteristics over age and sex classes in B6 male and female pairs of mice and in *Shank3*^−/−^ female pairs. Spectrograms of six examples of USVs in **(A)** B6 males, **(B)** B6 females, and **(C)** *Shank3*^−/−^ females aged 3 months (1,024 FFT points, 1.7 ms time resolution, Hamming window, 50% overlap). Comparisons of the duration of the USVs **(D)**, number of frequency modulations per USV **(E)**, and harsh index of the USVs **(F)** between males and females at 5 weeks (males: 630 USVs, females: 7,167 USVs), 3 months (males 688 USVs, females: 26,357 USVs) and 7 months (males: 241 USVs, females: 33,954 USVs) of age in B6 mice (Linear Mixed Model with sex as fixed factor and pair as random factor). Comparisons of the duration of USVs **(G)**, the number of frequency modulations per USV **(H)**, the number of frequency jumps **(I)**, the frequency at the beginning of the USV **(J)**, and the mean power of USVs **(K)** between B6 females (26,357 USVs, purple) and *Shank3*^−/−^ females (33,162 USVs, green) aged 3 months (Linear Mixed Model with genotype as fixed factor and pair as random factor). ns, not significant; uncorrected *p*-values: *p < 0.05, **p < 0.01. None of the tests survived a correction for multiple testing.

For clarity of presentation, we selected acoustic variables based on PCA over the whole set of B6 USVs and validated through correlations between acoustic variables (see Supplementary Methods—Selection of representative acoustic variables). The whole set of acoustic features is available in the supplementary files. Spectrograms of six USVs for B6 males (Figure 4A), B6 females (Figure 4B) and *Shank3*^−/−^ females (Figure 4C) aged 3 months are given as examples. When all USVs were considered, we observed that mature B6 males emitted significantly shorter (Figure 4D), less modulated (Figure 4E) and less harsh (i.e., less noisy; Figure 4F) USVs compared to females (see also Supplementary Figure 7). These differences did not reach significance in juveniles (5 weeks of age) but only when animals were fully mature (i.e., at 3 and 7 months of age; Supplementary Figure 7). B6 males also emitted USVs with limited frequency difference between the start and the end points of the signal compared to females at any age (Supplementary Figure 7). Overall, USVs emitted by males appeared to be shorter, purer, and flatter compared to females’ USVs.

When examining the influence of age on vocal production, we found that, in B6 females, the duration, frequency modulations, harshness, and loudness of USVs increased with increasing age, while the frequency-related traits decreased (Supplementary Figure 8). In males, this evolution with increasing age was more restricted, with a significant increase in duration, frequency, and harshness only between 3 and 7 months of age, as if it took more time for males to establish their social signals. Age-related variations in frequency-related traits appeared not to be stable between age classes, with the lowest frequency-related traits at 3 months of age (Supplementary Figure 8).

Contrary to our hypothesis of simpler vocal communication in *Shank3*^−/−^ mice, *Shank3*^−/−^ mice emitted USVs with acoustic characteristics that were not significantly different from those of B6 females (Figures 4G–J). There was only a trend for USVs to be starting at higher frequency (genotype: *z* = 2.31 [396.0, 4,775.1], *p* = 0.021; intercept: *z* = 75.66, *p* < 0.001 [63,713.9, 67,102.7]; Figure 4J) and to be quieter (i.e., less powerful) in *Shank3*^−/−^ mice compared to B6 female mice (genotype: *z* = −2.80 [−0.42, −0.07], *p* = 0.005; intercept: *z* = 15.22, *p* < 0.001 [0.90, 1.17]; Figure 4K; see also Supplementary Figure 9). The differences in the power characteristics were not related to differences in the proximity to the microphone (Supplementary Figures 10A,B). The slightly smaller body volume (previously reported for body size in Schmeisser et al., 2012) and here estimated through body surface on the tracking mask; Supplementary Figure 10C) of *Shank3*^−/−^ mice relative to that of B6 mice could partially explain the reduced power of *Shank3*^−/−^ mouse USVs.

We investigated further these variations related to age, sex, and genotype across the different contexts of emission. We aimed at testing whether the differences described above depend on the behavioral contexts.

### Acoustic Variations Related to the Context of Emission of USVs

#### Age- and Sex-Related Variations Across Contexts

**Figure 5:**
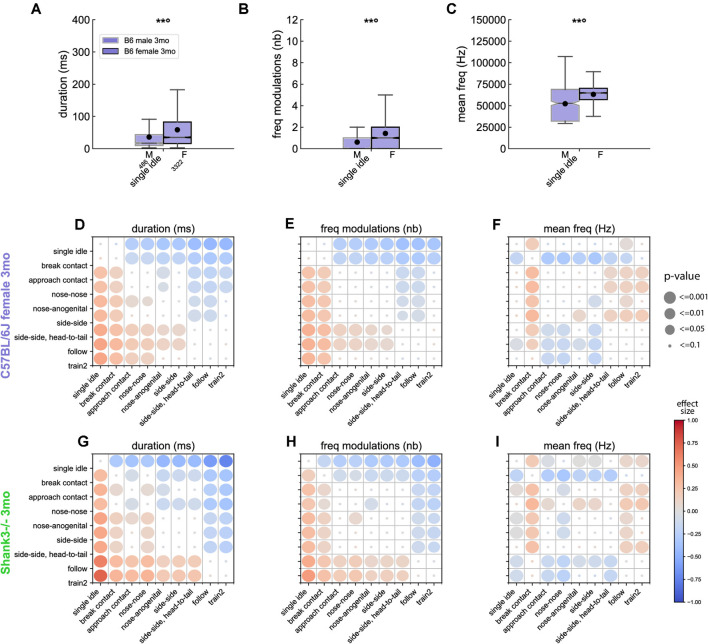
Acoustic variations of USVs between contexts of emission in B6 and *Shank3*^−/−^ mice. Sex-related variations in **(A)** the duration, **(B)** the number of frequency modulations, and **(C)** the mean frequency of USVs emitted during single idle by 3 months old male (486 USVs) and female (3,322 USVs) B6 mice (Linear Mixed Model with sex as fixed factor and pair as random factor; ns: not significant, uncorrected *p*-values: **p < 0.01: *p*-values followed by ° survived the correction for multiple testing for acoustic variables and events). Comparison of the duration **(D)**, number of frequency modulations **(E)** and mean frequency **(F)** of USVs emitted by 3 months old B6 females between the different contexts of emission (Linear Mixed Models with context as fixed effect and pair as random effect). Blue colors indicate that the acoustic feature of USVs given during y-event is lower than the acoustic feature of USVs given in x-event; red colors indicate that the acoustic feature of USVs given during y-event is higher than the acoustic feature of USVs given in x-event; the effect size is represented by the color scale while the significance levels are represented by the size of the circles. Comparison of the duration **(G)**, number of frequency modulations **(H)** and mean frequency **(I)** of USVs emitted by 3 months old *Shank3*^−/−^ females between the different contexts of emission (Linear Mixed Models with context as fixed effect and pair as random effect; the effect size is represented by the color scale while the significance levels corrected for multiple testing across acoustic variables and contexts combinations are represented by the size of the circles).

In B6 mice, sex-related acoustic variations were significant mostly in USVs emitted during a single idle (Supplementary Figures 11–13). The direction of variations differed between 5 weeks of age and mature age classes. Indeed, at 5 weeks of age, during single idle, females emitted USVs that were shorter, steeper, with less frequency modulations than males (Supplementary Figure 11). In contrast, at three (and to a lesser extent seven) months of age, in single idle, females emitted longer (Figure 5A), steeper, more modulated (Figure 5B), higher-pitched (Figure 5C), and more frequency-broken (i.e., more jumps) USVs compared to males (Supplementary Figures 12, 13).

In males, the variations observed with increasing age remained significant only in single idle (and to a lower extent in approaching before a contact and in nose-nose contacts), where frequency-related traits were minimum at 3 months of age (Supplementary Figure 14). In other behavioral contexts, either the number of USVs emitted was too low or the variations were not significant. In females, the variations related to age were consistent across all behavioral contexts, with increasing duration, frequency modulations, power, and harshness as well as decreasing frequency-related traits with increasing age (Supplementary Figure 15).

*Shank3*^−/−^ mice emitted USVs with higher frequency-related traits compared to B6 females in the least social contexts (single idle and break contact; Supplementary Figure 16). Interestingly, the reduced power detected in USVs emitted by *Shank3*^−/−^ females compared to B6 females was not significant in the most intense social context (Train2), while it was significant in other types of social contacts and in approach before a contact (Supplementary Figure 16). This suggests that the high level of arousal in the Train2 context masks the power deficit displayed by *Shank3*^−/−^ mice.

#### Variations of Acoustic Features Between Contexts

From this step of the analysis, we restricted our investigations to females, given the low number of USVs emitted by males in other contexts than single idle. In B6 females, the context specificity increased with increasing age. Indeed, at 5 weeks (Supplementary Figure 17) and 3 months (Supplementary Figure 18) of age, USVs emitted during single idle and break contact displayed significantly shorter duration (Figure 5D), lower frequency modulations (Figure 5E), lower harsh index compared to USVs emitted in other contexts. USVs emitted in both contexts were similar to each other, except for the mean frequency that was lower in break contact than in any other context (Figure 5F). In addition, the USVs emitted during the different types of close contacts (nose-nose, nose-anogenital, side-side) displayed similar frequency characteristics and modulations but differed from other social contexts (see example for the frequency modulations in Figure 5E). At 7 months of age (Supplementary Figure 19), USVs emitted during the different types of close contacts were again not significantly different. In contrast, USVs emitted during the approach preceding a contact emerged as acoustically different from the USVs emitted during close contacts, follow and train2 behaviors as if the aging animals refined their approach behavior.

In *Shank3^−/−^* mice recorded at 3 months of age, we observed that USVs emitted during Follow and Train2 behaviors were acoustically similar (Supplementary Figure 20), and both types significantly differed compared to USVs emitted in all other contexts, i.e., longer (Figure 5G), harsher, larger frequency modulations (Figure 5H) and lower mean frequency (Figure 5I). Interestingly, USVs emitted by *Shank3*^−/−^ mice during nose-to-nose contacts were significantly different [i.e., shorter duration (Figure 5G), lower frequency modulation (Figure 5H), higher-pitched (Figure 5I)] from USVs emitted in other types of contacts. This observation confirmed the specific impairment of *Shank3*^−/−^ mice to make nose-nose contacts (see above).

### Comparisons of Behaviors With and Without USVs

We next hypothesized that behaviors accompanied by USVs were more intense (i.e., longer and with higher mean speed) than behaviors not accompanied by USVs. As a proxy for arousal, we considered the speed of the animals. We thus quantified the mean speed of the animals during behaviors accompanied or not by USVs, as well as the duration of these behaviors.

**Figure 6:**
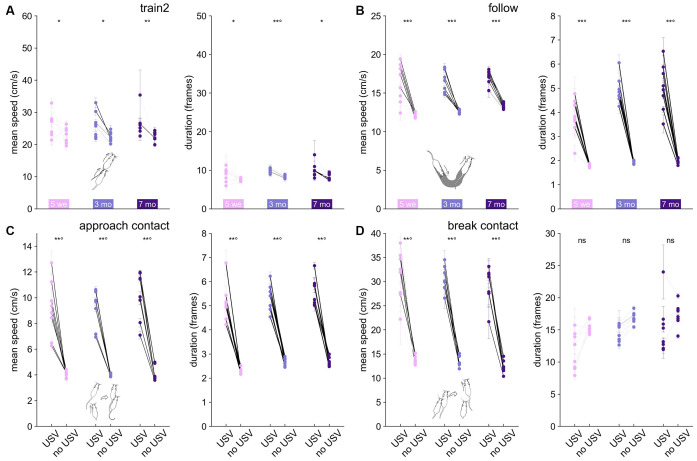
Variations in mean speed and duration of each behavioral event according to the presence or absence of USVs in B6 females. Variations of the mean speed of the animal performing the behavior (left panel) and of the event duration (right panel) for **(A)** Train2, **(B)** follow, **(C)** approach contact, and **(D)** break contact. Paired comparisons between behaviors with and without USVs were conducted at the group level using the paired Wilcoxon test for each age class (*p < 0.05, **p < 0.01; *p*-values remaining significant after correction for multiple testing over age classes were followed by °). Significant differences at the individual level using Mann–Whitney *U*-tests after Bonferroni correction are depicted by the color of the segments linking the mean value, with and without USVs. light gray: not significant, medium gray: *p* < 0.05, dark gray: *p* < 0.01, black: *p* < 0.001.

Our hypothesis was not verified for Train2 events in any age class at the individual level. Indeed, within each individual, the duration of the event and the speed of the mice did not vary significantly, regardless of whether USVs were emitted or not (Figure 6A). This might be related to the fact that this behavior was the most specific to USVs (Figure 3E). In contrast, follow (Figure 6B), approach contact (Figure 6C), contact, getaway, and oral-genital contact (data not shown) showed both a significantly higher speed and longer duration when accompanied by USVs, in agreement with our hypothesis at the individual level. For break contact, the presence of USVs was accompanied by higher speed but not necessarily a longer duration (Figure 6D). Overall, the relationship between USV emission and behavioral display (speed and duration of the event) suggests USVs can be considered as an expression of higher arousal in simple (e.g., follow, approach contact, break contact, oral-genital contact) social investigation, but not in the very intense and highly specific social investigation (such as Train2). These results obtained in B6 mice were also observed in *Shank3*^−/−^ mice (Supplementary Figure 21).

### Organization in Bursts

#### Event-Driven Determination of the Threshold for Burst Definition

We observed that USVs were spontaneously emitted in sequences that we call “USV bursts.” The inter-USV interval threshold to classify two successive USVs in two different USV bursts was determined by examining the optimal correlation between USV bursts and behavioral events (see Supplementary Methods—Determining intervals between USVs to define USV burst). We defined that USVs separated by intervals longer than 750 ms belong to two different bursts. This 750 ms threshold was valid in pairs of B6 males, in pairs of B6 females in all age classes, as well as in *Shank3*^−/−^ pairs.

#### Sex, Age, and Genotype Effects on Bursts Characteristics

**Figure 7:**
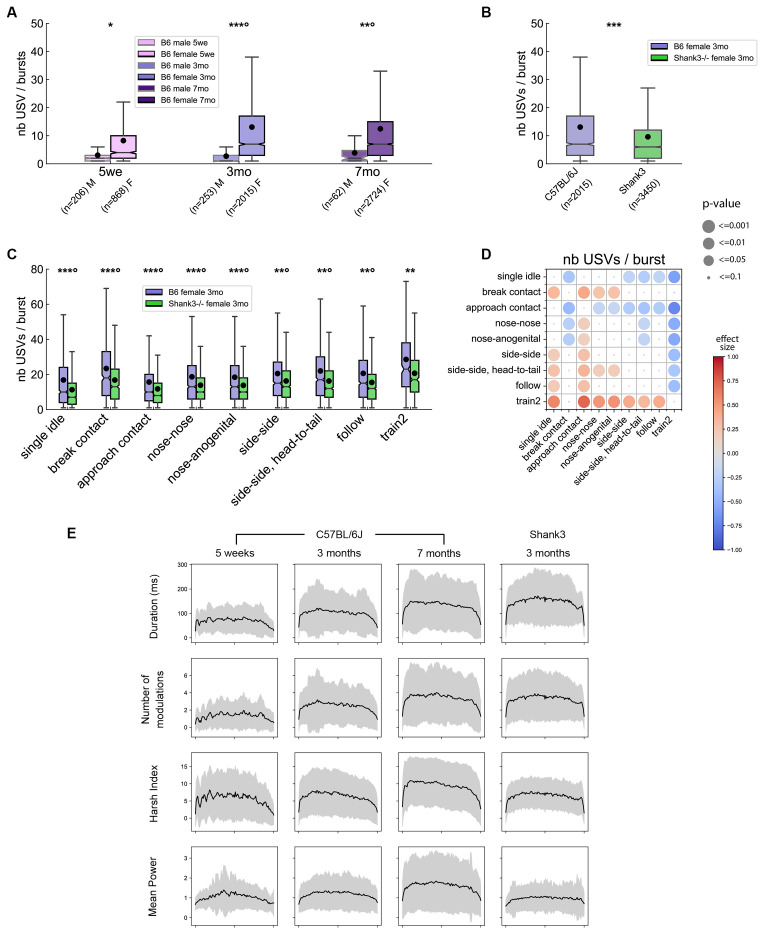
Bursts characteristics in male and female pairs of B6 mice and in *Shank3*^−/−^ female mice. **(A)** Comparison of the number of USVs per burst between B6 males and females at 5 weeks, 3 months and 7 months of age (Linear Mixed Model with sex as fixed factor and pair as random factor). **(B)** Comparison of the number of USVs per burst between B6 females and *Shank3*^−/−^ females aged 3 months (Linear Mixed Model with genotype as fixed factor and pair as random factor). **(C)** Comparison of the number of USVs per burst between B6 females and *Shank3*^−/−^ females aged 3 months across the different contexts of emission (Linear Mixed Model with sex as fixed factor and pair as random factor). ns: not significant, uncorrected *p*-values: *p < 0.05, **p < 0.01, ***p < 0.001; *p*-values followed by ° survived a correction for multiple testing (**A**: age classes, **B**: no correction, **C**: number of behavioral contexts). **(D)** Comparison of the number of USVs per burst emitted by 3 months old B6 females between the different contexts of emission (Linear Mixed Models with context as fixed factor and pair as random factor). Blue colors indicate that the number of USVs per burst given during y-event is lower than the number of USVs per burst given in x-event; red colors indicate that the number of USVs per burst given during y-event is higher than the number of USVs per burst given in x-event; the effect size is represented by the color scale while the significance levels are represented by the size of the circles. **(E)** Evolution of acoustic characteristics of USVs (duration, frequency modulations, harsh index, mean power) according to their position within a burst in B6 females aged 5 weeks, 3 months, and 7 months as well as in *Shank3*^−/−^ female mice aged 3 months.

Using this definition of a USV burst, we again observed a robust sex-related difference in the burst organization. Indeed, males tended to emit USV bursts containing less USVs than females at any age (Linear Mixed Models, hereafter LMM: 5 weeks: sex: *z* = −2.22 [−8.53, −0.52], *p* = 0.027; intercept: *z* = 5.83, *p* < 0.001 [5.20, 10.48]; 3 months: sex: *z* = −9.55, *p* < 0.001 [−12.45, −8.21], intercept: *z* = 29.87, *p* < 0.001 [12.19, 13.90]; 7 months: sex: *z* = −3.43, *p* = 0.001 [−12.12, −3.31], intercept: *z* = 14.89, *p* < 0.001 [10.11, 13.18]; Figure 7A). No significant age effect was detected for the number of USVs per USV burst in males. In contrast, females aged 5 weeks emitted USV bursts with fewer USVs than 3-month-old females (LMM: age: *z* = −7.77, *p* < 0.001 [−6.01, −3.64], intercept: *z* = 22.96, *p* < 0.001 [11.82, 14.021]), but the number of USVs per USV burst did not differ significantly between 3 and 7 months of age (age: *z* = 1.68, *p* = 0.093 [0.13, 1.69]; Figure 7A). Interestingly, the burst organization was perturbed in *Shank3*^−/−^ females. Indeed, at 3 months of age, *Shank3*^−/−^ mice emitted USV bursts that contained fewer USVs (mean ± standard deviation: 9.6 ± 10.6 USVs per USV burst) compared to age-matched B6 mice (13.1 ± 15.9 USVs per USV burst; LMM: genotype: *z* = −4.93, *p* < 0.001 [−5.29, −2.28], intercept: *z* = 21.96, *p* < 0.001 [11.87, 14.20]; Figure 7B). This was verified across all contexts (Figure 7C).

#### Context-Specificity of Burst Structure

Across all age classes, USV bursts emitted during single idle and approach contact were the shortest compared to bursts emitted in all other contexts in B6 pairs and in *Shank3*^−/−^ pairs (Figure 7D for B6 females aged 3 months; see Supplementary Figure 22 for other age classes and for between-contexts comparisons in *Shank3*^−/−^ females). In contrast, USV bursts emitted during Train2 and to a lesser extent in side-side, head-to-tail, and break contact were the longest (Figure 7D and Supplementary Figure 22). USV bursts emitted during the different types of close contacts were not different from one another.

#### Evolution of Variables Over the Bursts

For bursts containing nine or more USVs, we examined the evolution of the acoustic characteristics over the sequences of USVs. We restrained our analyses to females since such long USV bursts were extremely rare in males. In 3 months old B6 females, USVs appeared to be more complex in the middle of the USV bursts than at the extremities. This means that USVs in the middle of a burst displayed longer duration, larger frequency modulations, higher harsh index, and higher power in comparison with those at the beginning or at the end of the burst. These profiles were more subtle at 5 weeks of age and clearer at 7 months of age (Figure 7E, three left columns). Interestingly, *Shank3*^−/−^ mice displayed similar variations over the bursts, except for the mean power. Indeed, *Shank3*^−/−^ pairs showed a reduced ability to modulate the power of their USVs over a burst compared to B6 mice (Figure 7E, right column).

## Discussion

### Tackling the Complexity of Mouse Ultrasonic Vocalizations

In the vast majority of previous studies investigating mouse social communication, USVs were triggered by social deprivation (e.g., 2 weeks of isolation) and recorded in the first minutes or hours of interaction (Scattoni et al., 2011; Neunuebel et al., 2015; Warren et al., 2018a, 2020; Sangiamo et al., 2020). The USV types were either classified in a pre-determined repertoire (Scattoni et al., 2011) or simplified for modeling to reduce the complexity of the signals (e.g., ignoring harmonic components or frequency jumps or normalizing over duration; (Neunuebel et al., 2015; Warren et al., 2018a, 2020; Sangiamo et al., 2020; but see Warren et al., 2021). In our study, we provide a complementary approach by determining a large set of acoustic variables to avoid masking the complexity of the signals and leave the door open for any user to design their own classification as in Kikusui et al. (2021). This approach has the limit of inducing a large number of statistical tests over the different acoustic traits. In addition, the reduced number of pairs here recorded allowed to highlight only large effects between age or sex classes, and also between genotypes, despite the large amount of USVs recorded from each pair. We kept this limit in mind in our exploratory study, and we remain as cautious as possible throughout the study in the interpretation of the results. Future studies will elaborate on these results which work as working paths.

We chose not to build an exhaustive repertoire, as the link between meaning and classification can be a pitfall due to the normalization of time and frequency, which provides the same meaning to short, long, or differently dynamic USVs. We chose to examine specific acoustic characteristics and relate them directly to the behavioral context. This approach should help to understand the information carried in USVs (Hertz et al., 2020). We observed that USVs emitted during intense social interactions were longer, harsher, more modulated, and showed more frequency jumps than USVs emitted during non-social behaviors or at the end of social contacts (Figure 5). This is reminiscent of USVs emitted by wild house mice during social contact, which are longer than USVs emitted when standing alone (near a food spot, in the nest; Hoier et al., 2016).

### Hypotheses About the Functions of USVs

As previously observed in captive wild house mice (von Merten et al., 2014), we confirm over a long period that males in same-sex pairs emit few spontaneous USVs. Over short-term experiments, C57BL/6J male-male pairs also emitted the lowest number of USVs compared to male-female and female-female pairs, with the shortest bouts and simplest USVs (Matsumoto and Okanoya, 2021). In contrast, socially-deprived males have been shown to emit a non-negligible number of USVs (e.g., Chabout et al., 2012; Hammerschmidt et al., 2012; Ferhat et al., 2015). In these previous studies, the vocal repertoire did not differ significantly between males and females (Hammerschmidt et al., 2012; Ey et al., 2013; but see Matsumoto and Okanoya, 2021) and both sexes emitted USVs mostly during ano-genital sniffing and approach behavior (Ferhat et al., 2015, 2016). In the present study, males emitted USVs in short bursts with context-specific rates a 100 times lower than that of females, probably because females were more active (longer distance traveled during the night) and spent more time in social interactions than males (Supplementary Figure 1; see also de Chaumont et al., 2019). Overall, because males are slightly less social and active than females, they may encounter fewer situations that trigger USVs. Nevertheless, this incommensurate decrease in call rate relative to females may be related to the natural social structure of mice, with only subordinate males regrouped in same-sex subgroups (Palanza et al., 2005). Other communication modalities, such as body posture and tactile contacts, may be sufficient for males to regulate their social interactions.

We observed that females emitted USVs mostly during intense social interactions, such as Train2, and follow behaviors. The speed of the mice was also significantly higher during behaviors associated with USVs than those without (Figure 6). It is thus possible that USVs reflect a high level of arousal, at least in C57BL/6J, the mouse strain tested here. The emission of a large number of spontaneous USVs suggests that female mice are sufficiently aroused in their “home-cage-like” life. In short-term experiments, socially-housed mice never reach this level of excitement during the first minutes of interaction (probably focusing on the exploration of the environment) and therefore emit few USVs (e.g., Hammerschmidt et al., 2012; Ey et al., 2018). The emission of a large quantity of USVs can only be reached by social deprivation over such short periods. Social behaviors, such as follow, or approach contact, were significantly longer when accompanied by USVs than when occurring without (Figure 6). This result parallels the reported increased duration of chasing when USVs were emitted by females chased by males (Neunuebel et al., 2015). It is thus possible that some USVs are emitted only when a certain level of excitement is reached, for example after a specific time spent in contact. Whether certain USVs serve to maintain social contacts is currently unknown and would need to be tested using playback experiments synchronized with behavioral monitoring.

### Subtle Social Communication Abnormalities in *Shank3* Mutant Mice

In our setting, *Shank3^−/−^* mice were less active, took a longer time to approach, and shorter time to escape their conspecific (Supplementary Figure 3), while individual contact events tended to be longer than those of WT mice (Supplementary Figure 4). These activity and social specificities are consistent with the results of previous studies using the same mutant mouse strain (Vicidomini et al., 2017; de Chaumont et al., 2019). Other genetic models of *Shank3* mutant mice also display reduced social interest (*Shank3*-KO ex4–9: Bozdagi et al., 2010; Wang et al., 2011; Yang et al., 2012; *Shank3*-KO in ex21: Duffney et al., 2015; *Shank3* ex4–9: Jaramillo et al., 2016; *Shank3*-cKI: Mei et al., 2016; *Shank3* exon 4–22 complete KO: Wang et al., 2016; *Shank3B*: Balaan et al., 2019; *Shank3* exon 13–16: Peca et al., 2011; Dhamne et al., 2017; Fourie et al., 2018; *Shank3*B^+/–^: Orefice et al., 2019; Pagani et al., 2019). Nevertheless, other models failed to detect any atypical social interest in *Shank3* mutant mice (*Shank3*-KO ex4–9: Drapeau et al., 2014; *Shank3-*KO ex9: Lee et al., 2015; *Shank3*-KO ex 21: Kouser et al., 2013; *Shank3*-KO and HZ in ex21: Speed et al., 2015; *Shank3*-KO ex11–21 rat model: Song et al., 2019; conditional *Shank3* exon 4–22 knockout in the forebrain, striatum, and striatal D1 and D2 cells: Bey et al., 2018; *Shank3*^+/Q321R^ and *Shank3*^Q321R/Q321R^: Yoo et al., 2019). Such variability may be the consequence of differences in the type of mutation, the behavioral protocols, or housing conditions.

There are few studies that have investigated ultrasonic communication in *Shank3* mutant mice. Pup isolation calls were not affected in *Shank3*-KO ex4–9 mice (Yang et al., 2012; Jaramillo et al., 2016), the *Shank3*-KO ex11–21 rat model (Song et al., 2019), or *Shank3b*-KO mice (Balaan et al., 2019), whereas those of complete *Shank3-*KO ex4–22 mice showed a lower rate, as well as shorter duration, lower frequency, and lower amplitude (Wang et al., 2016). In the context of a male briefly interacting with an estrus female, the rate of USV emission was not affected in *Shank3-*KO ex21 (Kouser et al., 2013) or *Shank3-*KO ex4–9 mice (Yang et al., 2012). Nevertheless, it was lower in *Shank3B*-KO mutant males (Dhamne et al., 2017; Pagani et al., 2019) and higher in *Shank3-*KO ex4–9 males (Wang et al., 2011; Jaramillo et al., 2016) than in WT male mice. In *Shank3*^Q321R/Q321R^ mutant mice, the number of USVs in male-female interactions was not significantly different from that of WT mice, but *Shank3*^+/Q321R^ mutants showed a higher mean duration of USVs than WT mice (Yoo et al., 2019). Male complete *Shank3-*KO ex4–22 mice also showed a lower rate of USVs than WT males when encountering an estrus female, but they were of shorter duration, reduced amplitude, and normal peak frequency (Wang et al., 2016). One strength of our study is that it was designed to directly relate communication deficits with behavioral deficits. For example, the lower call rate of Shank3^−/−^ mice on the second night may be related to a significantly reduced total duration of Train2 and significantly fewer of these Train2 events (data not shown), a behavior that triggers a high rate of USV emission in WT mice.

Concerning USV bursts, *Shank3^−/−^* mice emitted fewer USVs per burst, but their USVs were weaker than those of B6 mice, with stable power throughout the burst, a trait rarely observed in B6 mice. Variations in USV duration were shown to be present in the *Shank3* mutant carrying the Q321R point mutation (Yoo et al., 2019), while the reduction of amplitude/power was also highlighted in the *Shank3* KO ex4–22 mice (Wang et al., 2016), in parallel with the unstable dominance hierarchy in complete *Shank3* KO triads relative to WT triads (Wang et al., 2016). Overall, in our hands, *Shank3*^−/−^ mice were able to emit USVs with similar acoustic structures and used them in similar contexts as B6 mice but perturbed hierarchical relationships may modify the structure of the USV bursts.

### Perspectives

Here, we showed sex- and age-related differences in the spontaneous communication of mice. Major quantitative and qualitative differences emerged between males and females from 3 months of age on. Females emitted more USVs than males, specifically during intense social investigations. With increasing age, mice emitted longer and more complex USVs, with specific differences between USV acoustic structure and behavioral contexts. Female *Shank3^−/−^* mice emitted USV bursts with fewer but weaker USVs than age-matched WT females. Future developments will include the implementation of the identification of the emitter. Indeed, identifying the sound source will refine the contexts in which USVs are emitted, allowing also to test the interaction between mice of different sexes or genotypes. Our system offers the possibility to characterize spontaneous mouse communication and paves the way for new studies investigating the complex interplay between genetic background, social experience, and hierarchy in the richness of social communication.

## Data Availability Statement

The datasets presented in this study can be found in online repositories. The names of the repository/repositories and accession number(s) can be found below: Zenodo repository (DOI: 10.5281/zenodo.5060503).

## Ethics Statement

The animal study was reviewed and approved by Ethical committee of the Institut Pasteur (CETEA n°89), followed by the approval of the Ministère de l’Enseignement Supérieur, de la Recherche et de l’Innovation under the reference APAFIS#7706-2016112317252460 v2.

## Author Contributions

FC created the segmentation and analysis methods and performed the data mining. SC and NL performed the genotyping of *Shank3* mutant mice. EE designed the study, performed and analyzed experiments. EE, FC, and TB conceived the project and wrote the manuscript. All authors contributed to the article and approved the submitted version.

## Conflict of Interest

The authors declare that the research was conducted in the absence of any commercial or financial relationships that could be construed as a potential conflict of interest.

## Publisher’s Note

All claims expressed in this article are solely those of the authors and do not necessarily represent those of their affiliated organizations, or those of the publisher, the editors and the reviewers. Any product that may be evaluated in this article, or claim that may be made by its manufacturer, is not guaranteed or endorsed by the publisher.

## Footnotes

^1^ https://usv.pasteur.cloud

^2^ https://packetsender.com/

^3^ https://github.com/fdechaumont/LMT-USV-Toolbox
