# Supplementary material for: LMT USV Toolbox, a Novel Methodological Approach to Place Mouse Ultrasonic Vocalizations in Their Behavioral Contexts—A Study in Female and Male C57BL/6J Mice and in Shank3 Mutant Females
Source: Front Behav Neurosci. 2021 Oct 13;15:735920. doi: 10.3389/fnbeh.2021.735920 (PMC8548730; doi:10.3389/fnbeh.2021.735920)
Supplement: Supplementary file 1 [file Data_Sheet_1.pdf]

Supplementary Figures

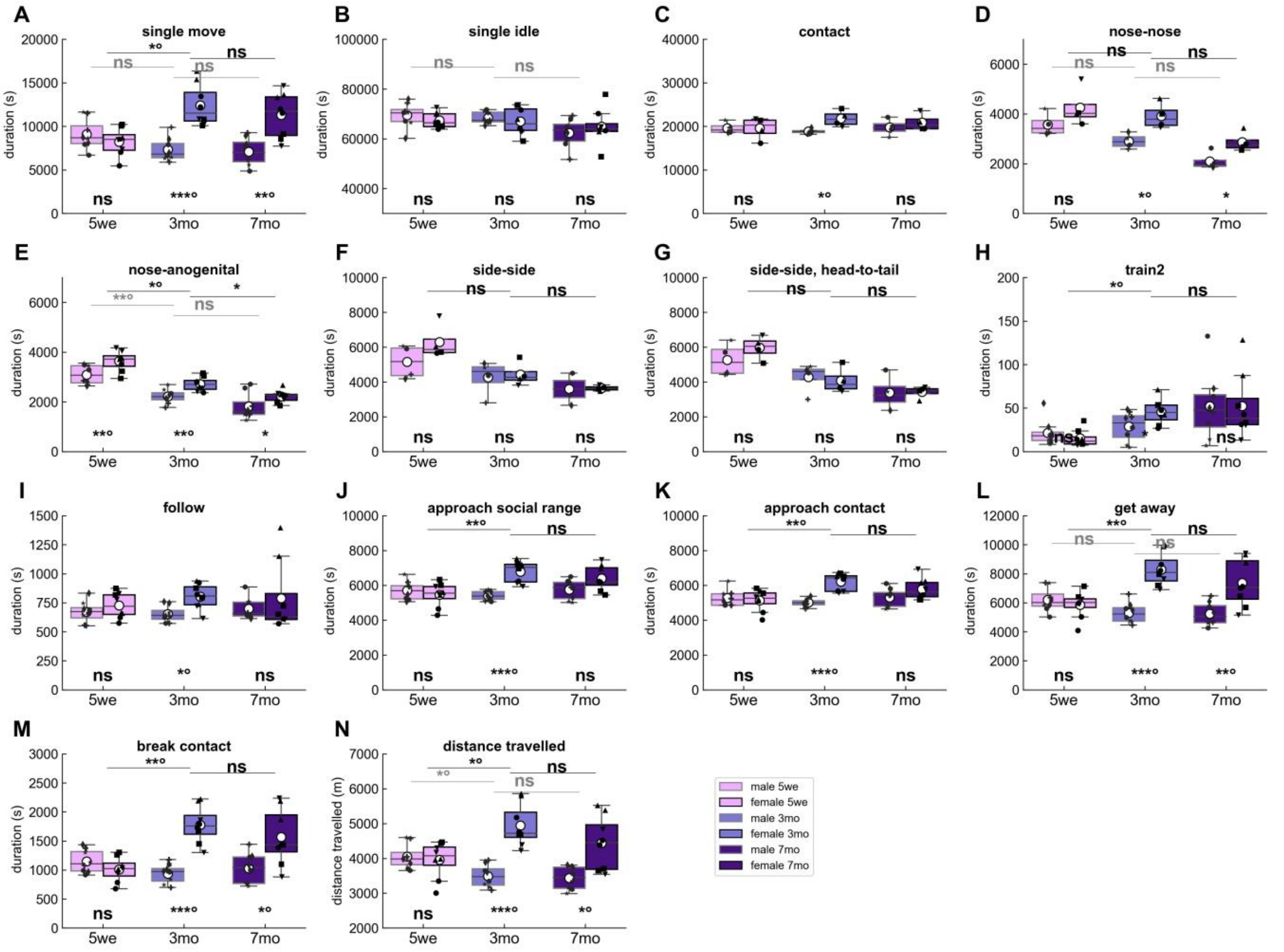

Supplementary Figure S1. Behavioral profile of B6 male and female mice aged of 5 weeks, 3 months and 7 months. We depicted the total time spent in each behavior over the three nights. Each black dot corresponds to an individual in A, B, E, H, I, J, K, L, M and N with similar shape for the two individuals of the pair, while in C, D, F, and G each dot corresponds to a pair since the behaviors are symmetrical (Mann-Whitney U-tests used to test for differences between sexes within each age class; Kruskal-Wallis test followed by Wilcoxon paired test if significant to test for differences between 5 weeks and 3 months and between 3 months and 7 months within each sex). Uncorrected p-values: ns: not significant, *: $p < 0.05$, **: $p < 0.01$, ***: $p < 0.001$. P-values followed by ° survived after correction for multiple testing.

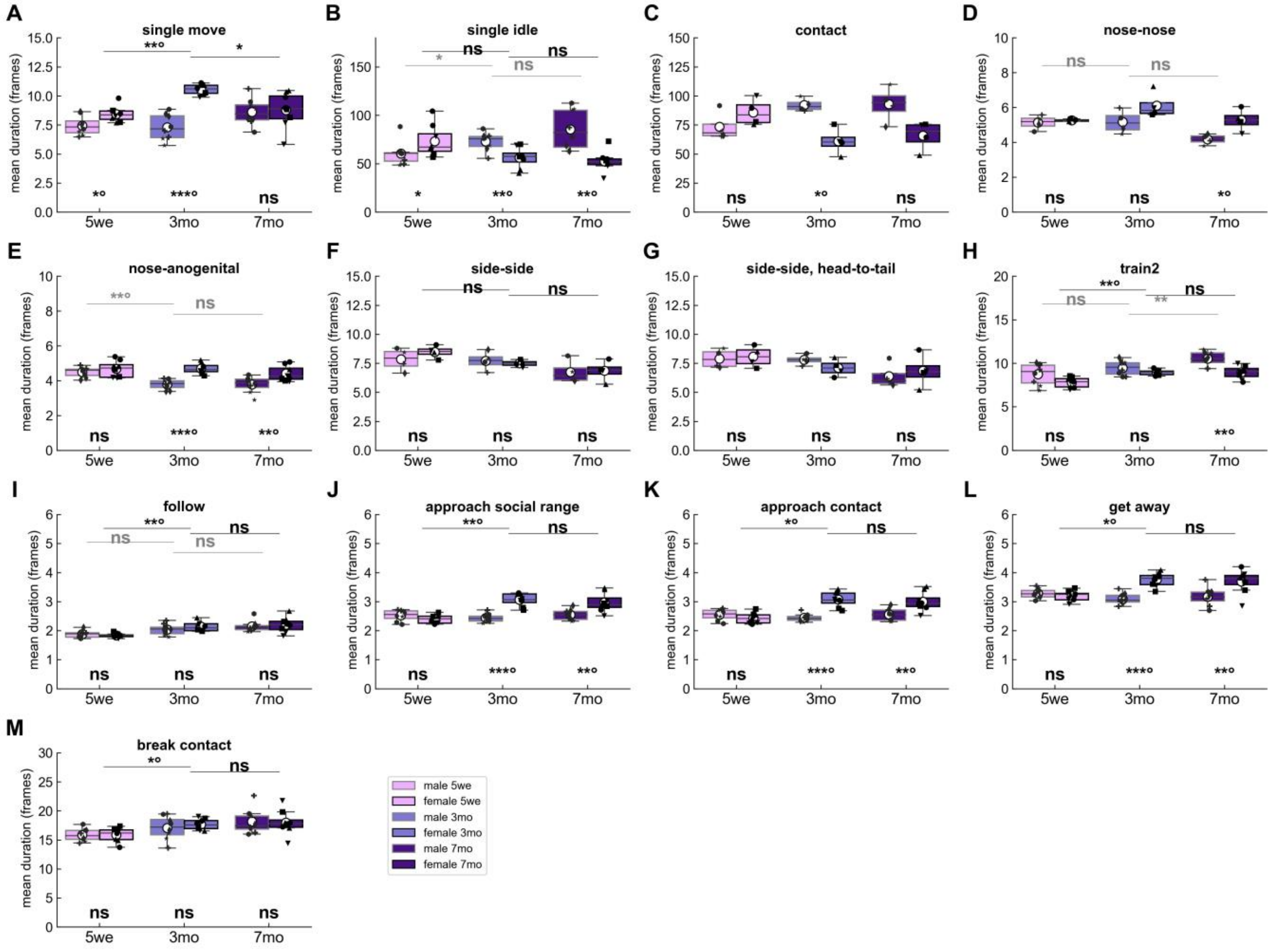

Supplementary Figure S2. Behavioral profile of B6 male and female mice aged 5 weeks, 3 months and 7 months. We depicted the mean duration of each behavior over the three nights. Each black dot corresponds to an individual in A, B, E, H, I, J, K, L, M and N with similar shape for the two individuals of the pair, while in C, D, F, and G each dot corresponds to a pair since the behaviors are symmetrical (Mann-Whitney U-tests used to test for differences between sexes within each age class; Kruskal-Wallis test followed by Wilcoxon paired test if significant to test for differences between 5 weeks and 3 months and between 3 months and 7 months within each sex). Uncorrected p-values: ns: not significant, *: $p < 0.05$, **: $p < 0.01$, ***: $p < 0.001$. P-values followed by ° survived after correction for multiple testing.

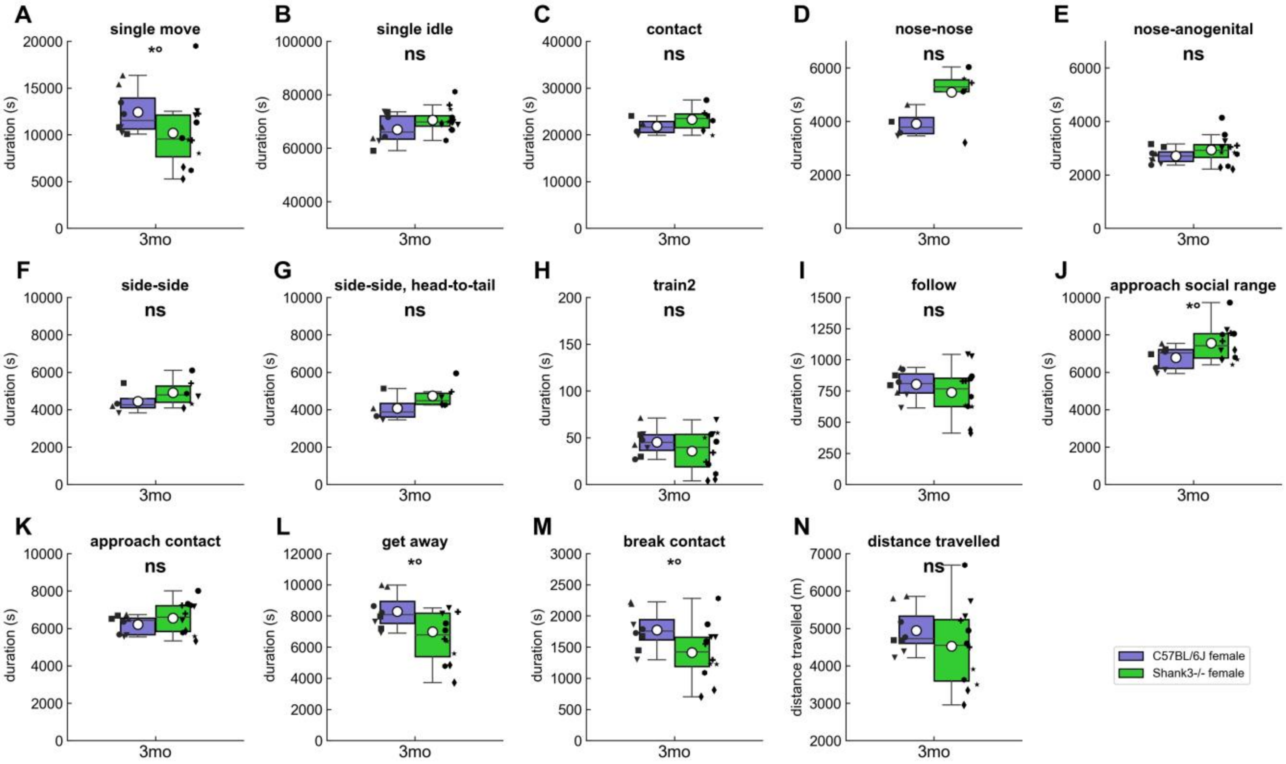

Supplementary Figure S3. Behavioral profile of B6 and *Shank3*$^{-/-}$ female mice aged 3 months. We depicted the total time spent in each behavior over the three nights. Each black dot corresponds to an individual in A, B, E, H, I, J, K, L, M and N with similar shape for the two individuals of the pair, while in C, D, F, and G each dot corresponds to a pair since the behaviors are symmetrical (Mann-Whitney U-tests used to test for differences between genotypes). Uncorrected p-values: ns: not significant, *: $p < 0.05$, **: $p < 0.01$, ***: $p < 0.001$. P-values followed by ° survived after correction for multiple testing.

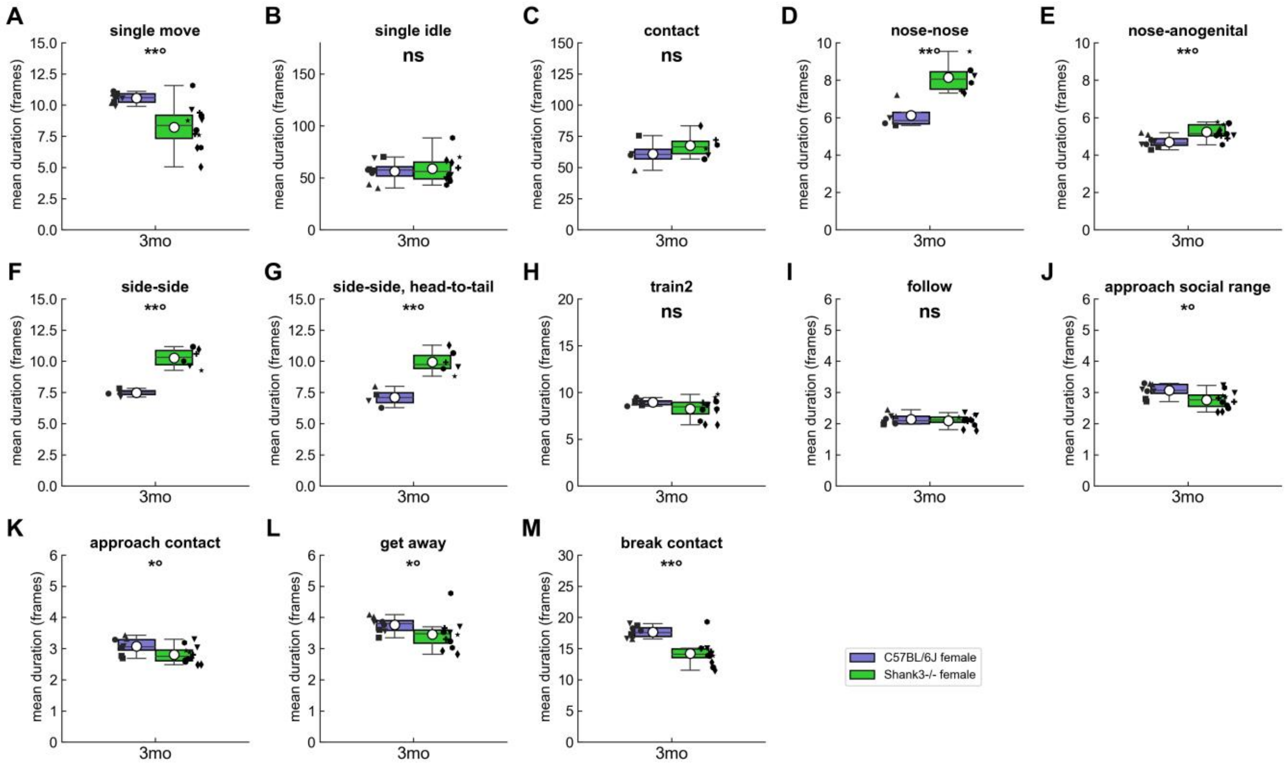

Supplementary Figure S4. Behavioral profile of B6 and *Shank3*$^{-/-}$ female mice aged 3 months. We depicted the mean duration of each behavior over the three nights. Each black dot corresponds to an individual in A, B, E, H, I, J, K, L, M and N with similar shape for the two individuals of the pair, while in C, D, F, and G each dot corresponds to a pair since the behaviors are symmetrical (Mann-Whitney U-tests used to test for differences between genotypes). Uncorrected p-values: ns: not significant, *: $p < 0.05$, **: $p < 0.01$, ***: $p < 0.001$. P-values followed by ° survived after correction for multiple testing.

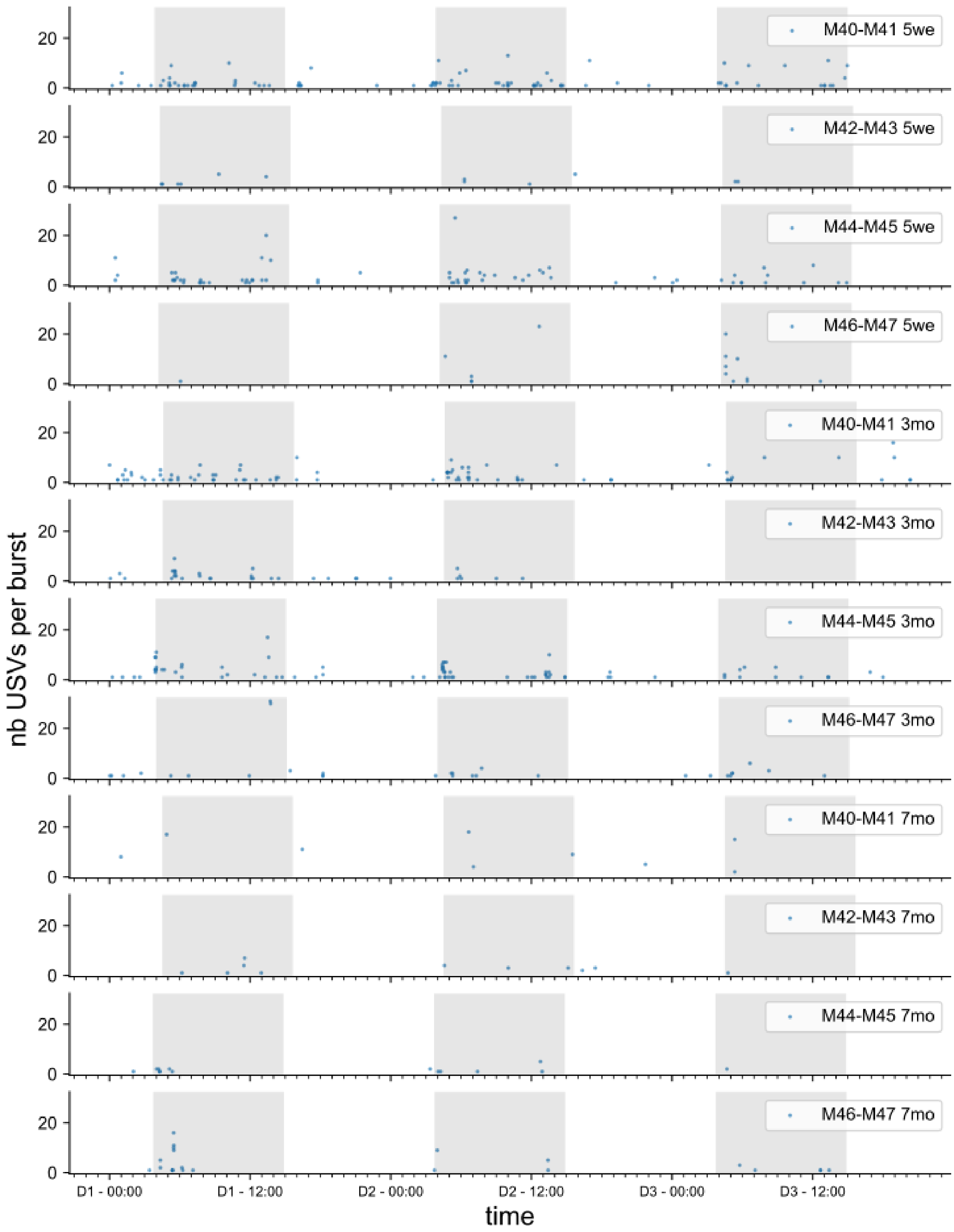

Supplementary Figure S5. Timeline of USV burst emission in B6 male pairs. We represented the amount of USVs per burst as a function of the time of emission over the three days of recording in B6 male pairs, recorded at 5 weeks, 3 months and 7 months.

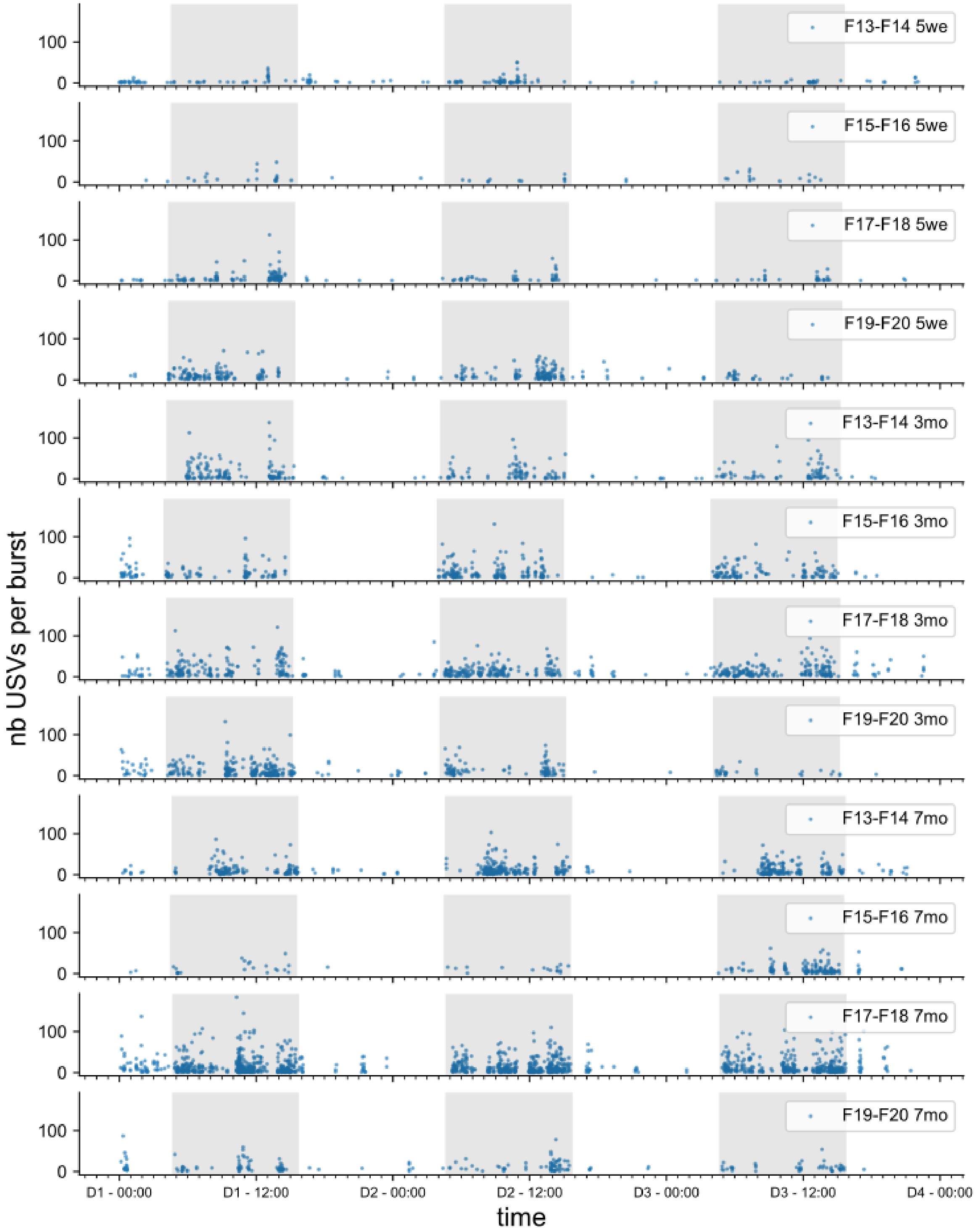

Supplementary Figure S6. Timeline of USV burst emission in B6 female pairs. We represented the amount of USVs per burst as a function of the time of emission over the three days of recording in B6 female pairs, recorded at 5 weeks, 3 months and 7 months.

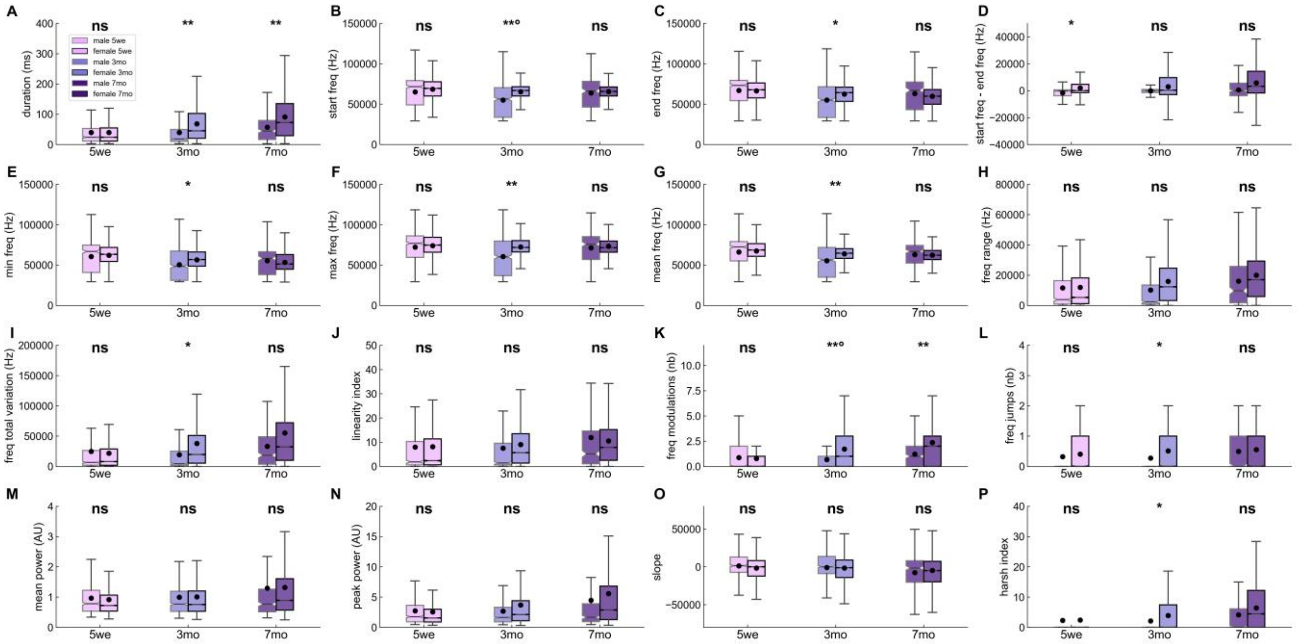

Supplementary Figure S7. Variations of acoustic features between males and females in B6 pairs at 5 weeks (males: 630 USVs, females: 7167 USVs), 3 months (males 688 USVs, females: 26357 USVs) and 7 months (males: 241 USVs, females: 33954 USVs) of age. (A) duration of USVs, (B) start frequency, (C) end frequency, (D) difference between the frequency at the end of the USV and the frequency at the start of the frequency, (E) minimum frequency, (F) maximum frequency, (G) mean frequency, (H) frequency range, (I) total variations of the frequency, (J) linearity index, (K) number of frequency modulations, (L) number of frequency jumps, (M) mean power of the USVs, (N) peak power, (O) general slope, and (P) harsh index. Linear Mixed model with sex as fixed factor and pair as random factor; ns: not significant, uncorrected p-values: *: $p < 0.05$, **: $p < 0.01$, ***: $p < 0.001$. P-values followed by ° survived after correction for multiple testing over acoustic variables.

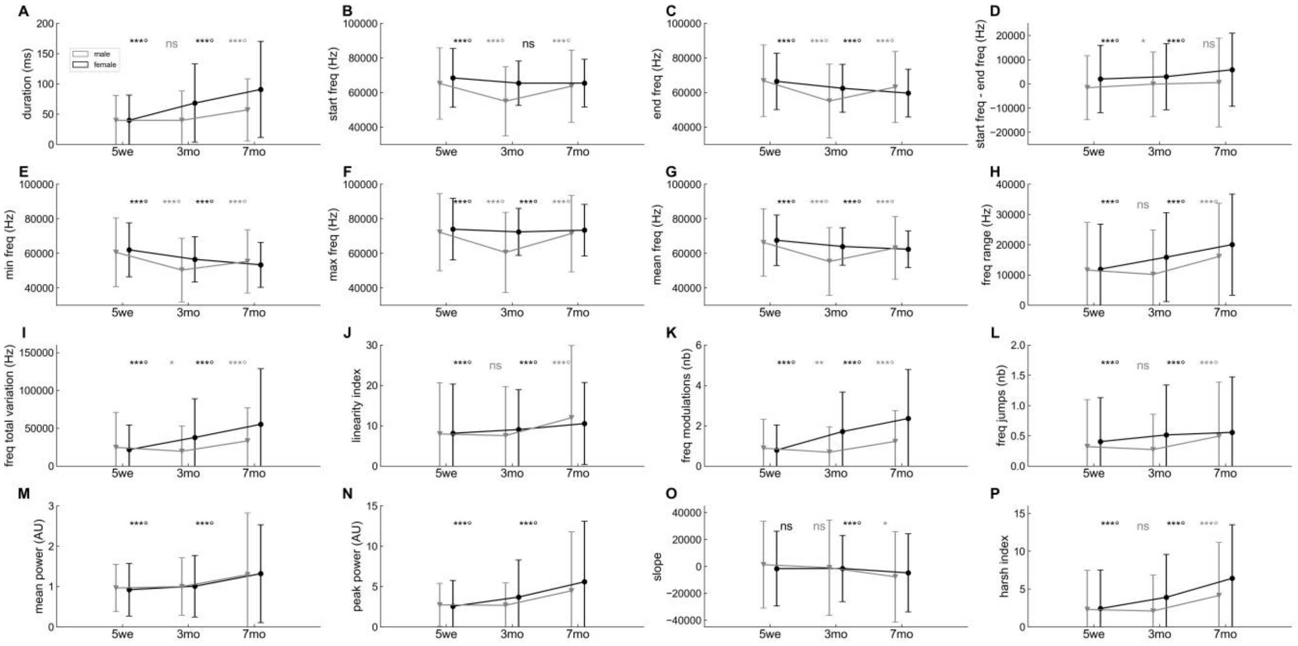

Supplementary Figure S8. Evolution with increasing age of acoustic features in B6 males and in B6 females. (A) duration of USVs, (B) start frequency, (C) end frequency, (D) difference between the frequency at the end of the USV and the frequency at the start of the frequency, (E) minimum frequency, (F) maximum frequency, (G) mean frequency, (H) frequency range, (I) total variations of the frequency, (J) linearity index, (K) number of frequency modulations, (L) number of frequency jumps, (M) mean power of the USVs, (N) peak power, (O) general slope, and (P) harsh index. For each sex separately, Kruskall-Wallis test followed by Student's T-tests between 5 week and 3 months and between 3 months and 7 months, p-values corrected for multiple testing over acoustic variables and age classes; ns: not significant, uncorrected p-values: *: $p < 0.05$, **: $p < 0.01$, ***: $p < 0.001$. P-values followed by ° survived after correction for multiple testing over acoustic variables.

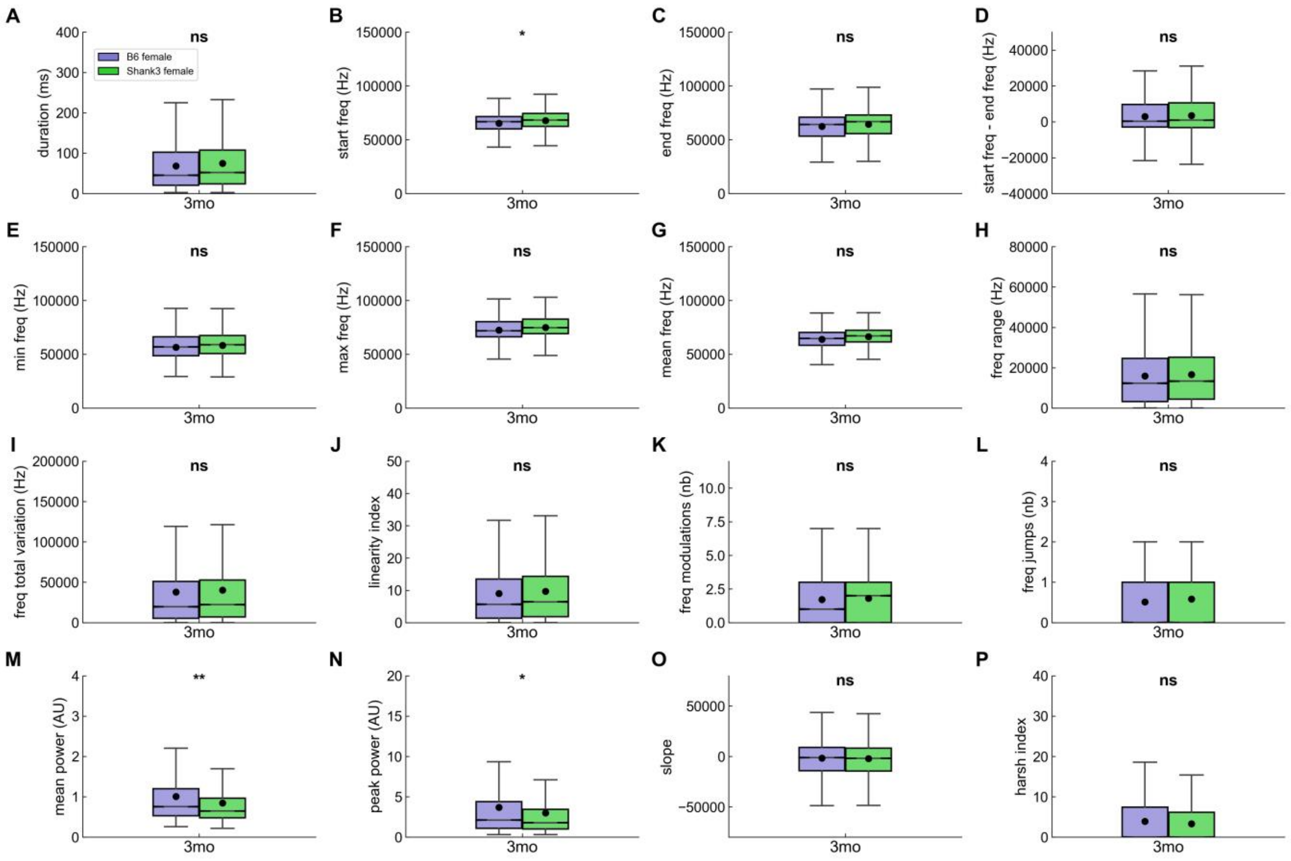

Supplementary Figure S9. Variations of acoustic features between B6 females and *Shank3*-/- females aged 3 months. (A) duration of USVs, (B) start frequency, (C) end frequency, (D) difference between the frequency at the end of the USV and the frequency at the start of the frequency, (E) minimum frequency, (F) maximum frequency, (G) mean frequency, (H) frequency range, (I) total variations of the frequency, (J) linearity index, (K) number of frequency modulations, (L) number of frequency jumps, (M) mean power of the USVs, (N) peak power, (O) general slope, and (P) harsh index. Linear Mixed Model with genotype as fixed factor and pair as random factor (B6 26357 USVs versus *Shank3*-/- 33162 USVs); ns: not significant; uncorrected p-values: *: $p < 0.05$, **: $p < 0.01$, ***: $p < 0.001$. P-values followed by ° survived after correction for multiple testing over acoustic variables.

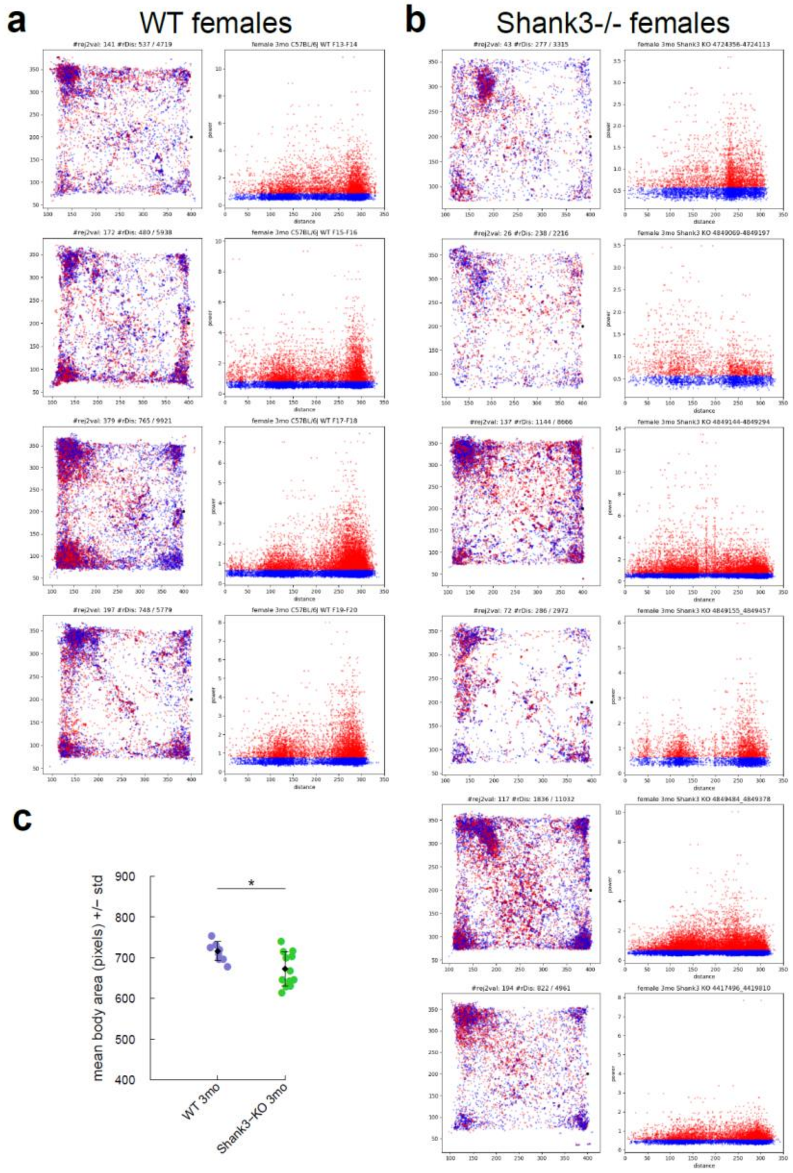

Supplementary Figure S10. Smaller body size might explain the lower power in USVs from *Shank3*$^{-/-}$ compared to WT more than the distance to the microphone during USV emission. Location of the pairs of mice and distance to the microphone during USV emission in (a) WT mice and (b) *Shank3*$^{-/-}$ mice. Left graphs: The mean position of the pair of mice over the whole USV duration is represented only when the two mice were detected within 10 cm from one another. Blue points represent USVs that were emitted with a power in the lowest 50th percentile of the power distribution for each experiment separately. Red points represent USVs that were emitted with a power in the highest 50th percentile of the power distribution for each experiment separately. The position of the microphone is depicted by a black dot in the middle of the right hand side of the cage. Right graphs: The power of the USVs recorded did not appeared to be correlated with the distance between the microphone and the position of the pair of the mice (the two mice were within 10 cm from one another). (c) Mean body surface measured on the masking detected by the Live Mouse Tracker system on the first xx hours of tracking in *Shank3*$^{-/-}$ female mice and in age-matched WT female mice. Mann-Whitney U-test was used for statistical comparison.

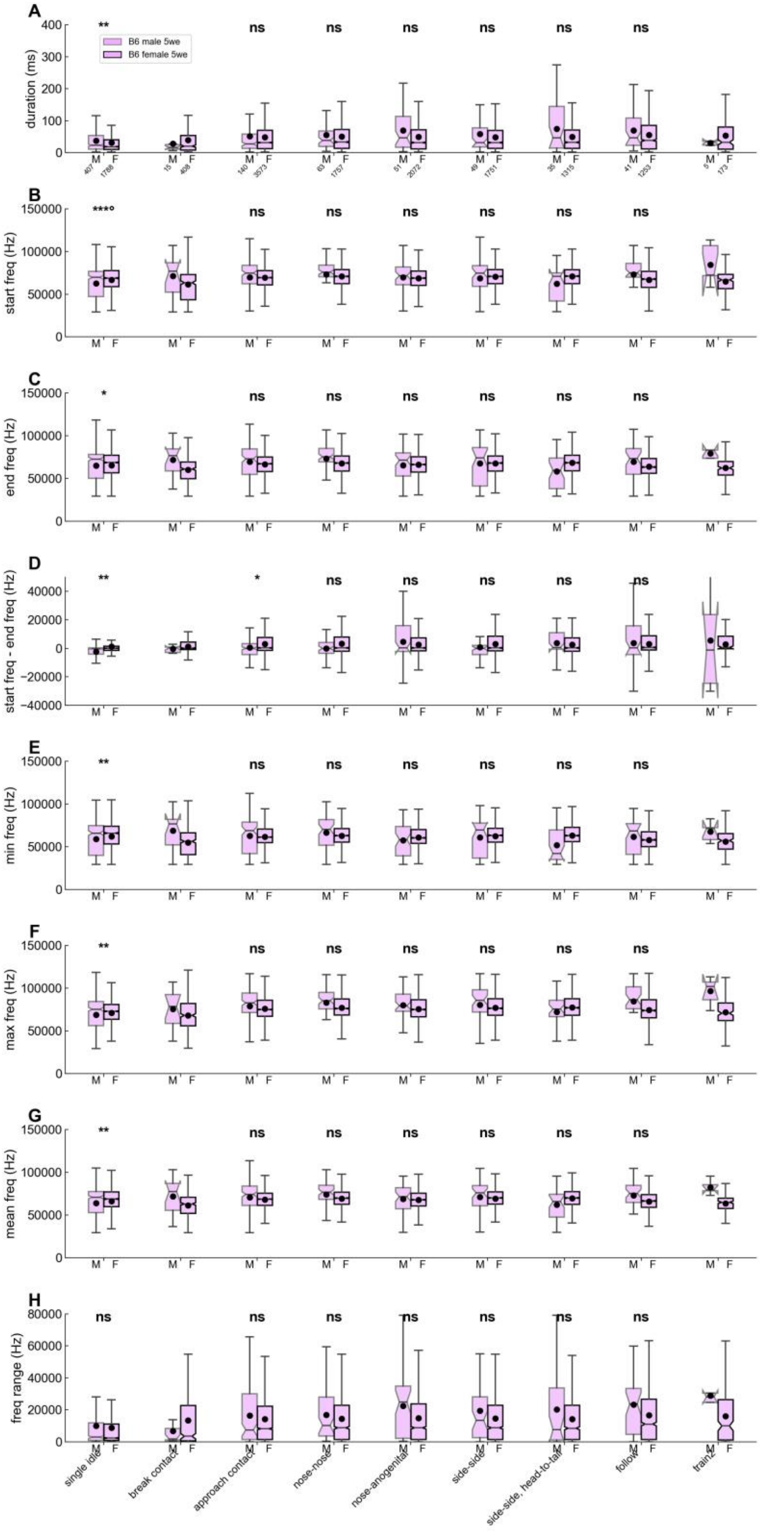
A
B
C
D
E
F
G
H
B6 male 5we
B6 female 5we
duration (ms)
start freq (Hz)
end freq (Hz)
start freq - end freq (Hz)
min freq (Hz)
max freq (Hz)
mean freq (Hz)
freq range (Hz)
single idle
break contact
approach contact
nose-nose
nose-anogenital
side-side
side-side, head-to-tail
follow
train2

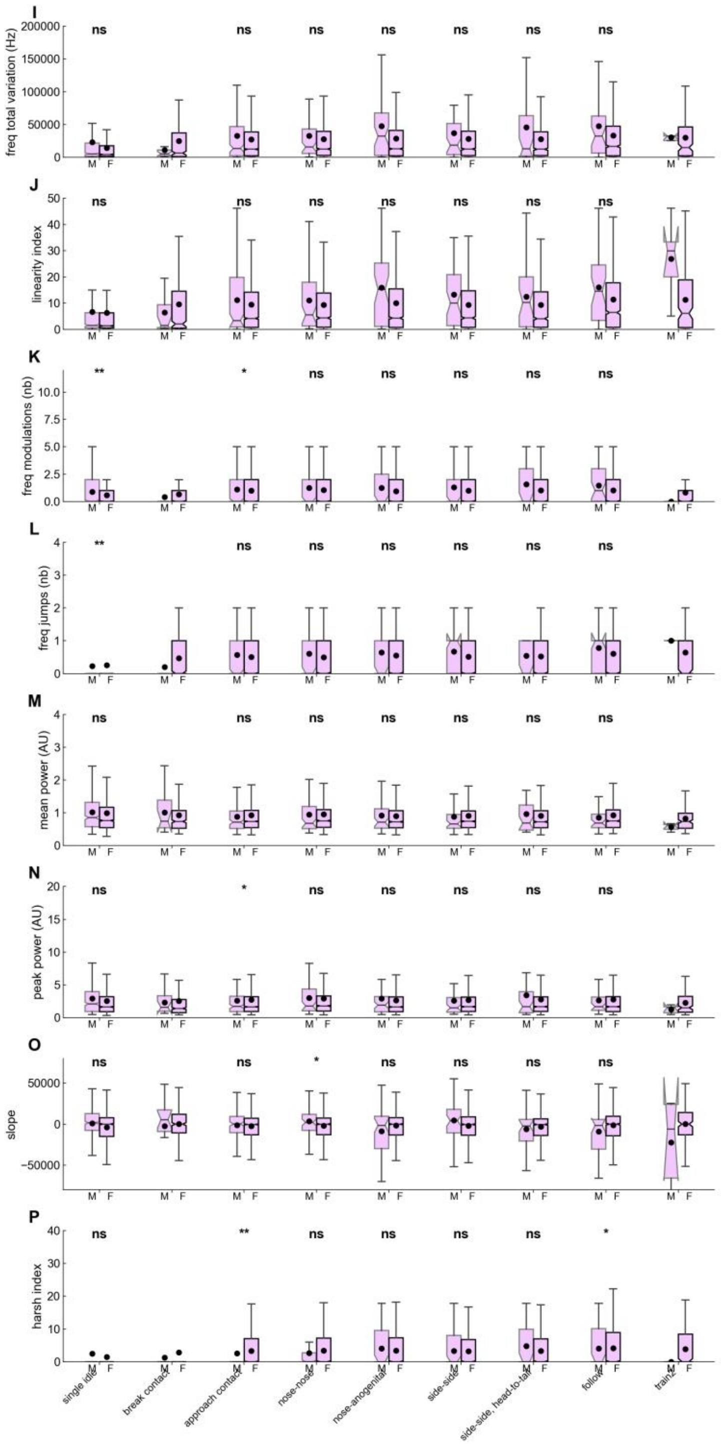

Supplementary Figure S11. Sex-related variations in acoustic features across contexts in B6 mice aged 5 weeks. Linear mixed model with sex as fixed factor and pair as random factor; ns: not significant; uncorrected p-values: *: $p < 0.05$, **: $p < 0.01$, ***: $p < 0.001$. P-values followed by ° survived after correction for multiple testing over acoustic variables and contexts.

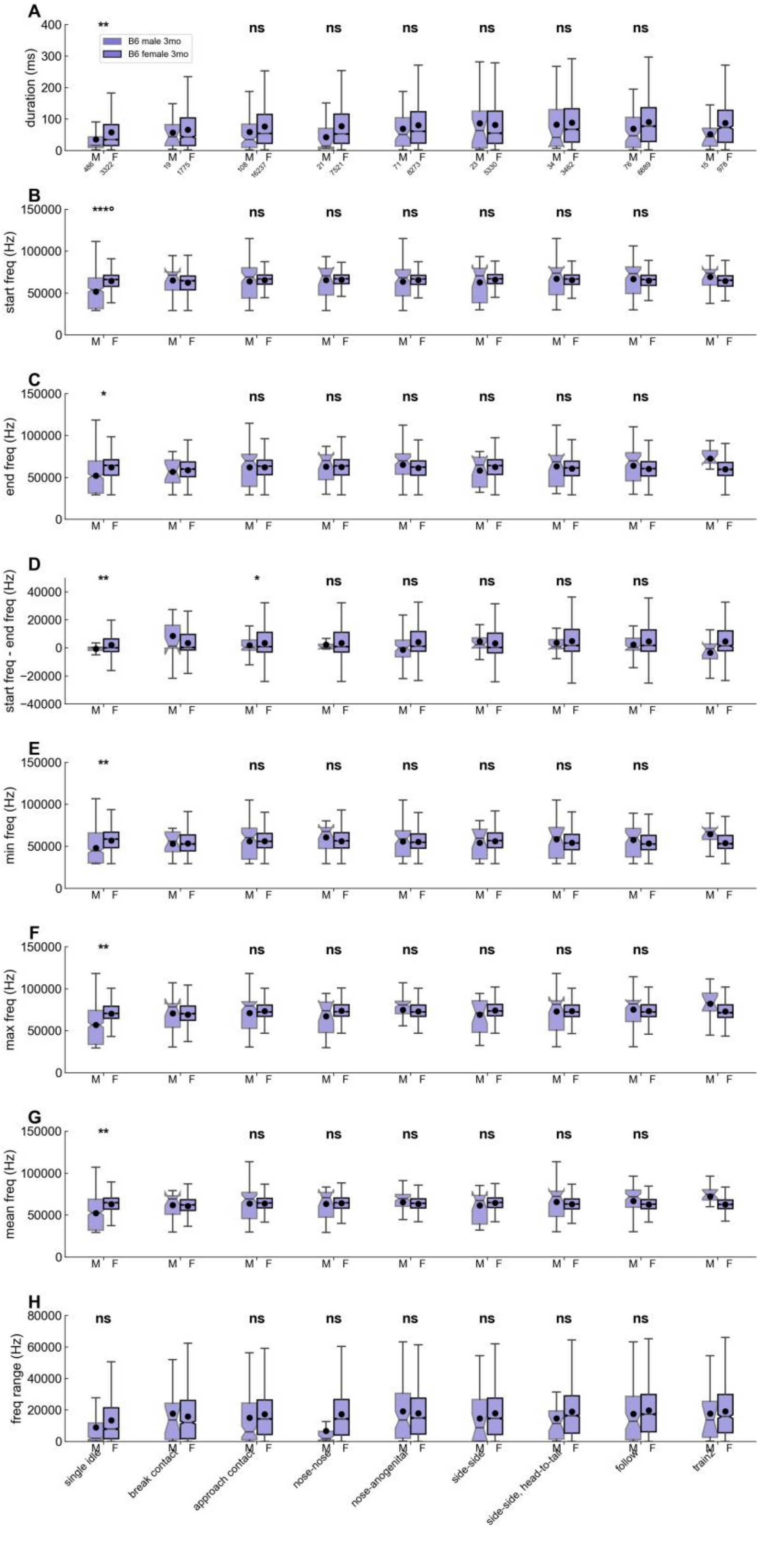

A
B
C
D
E
F
G
H
B6 male 3mo
B6 female 3mo
duration (ms)
start freq (Hz)
end freq (Hz)
start freq - end freq (Hz)
min freq (Hz)
max freq (Hz)
mean freq (Hz)
freq range (Hz)
single idle
break contact
approach contact
nose-nose
nose-anogenital
side-side
side-side, head-to-tail
follow
train2

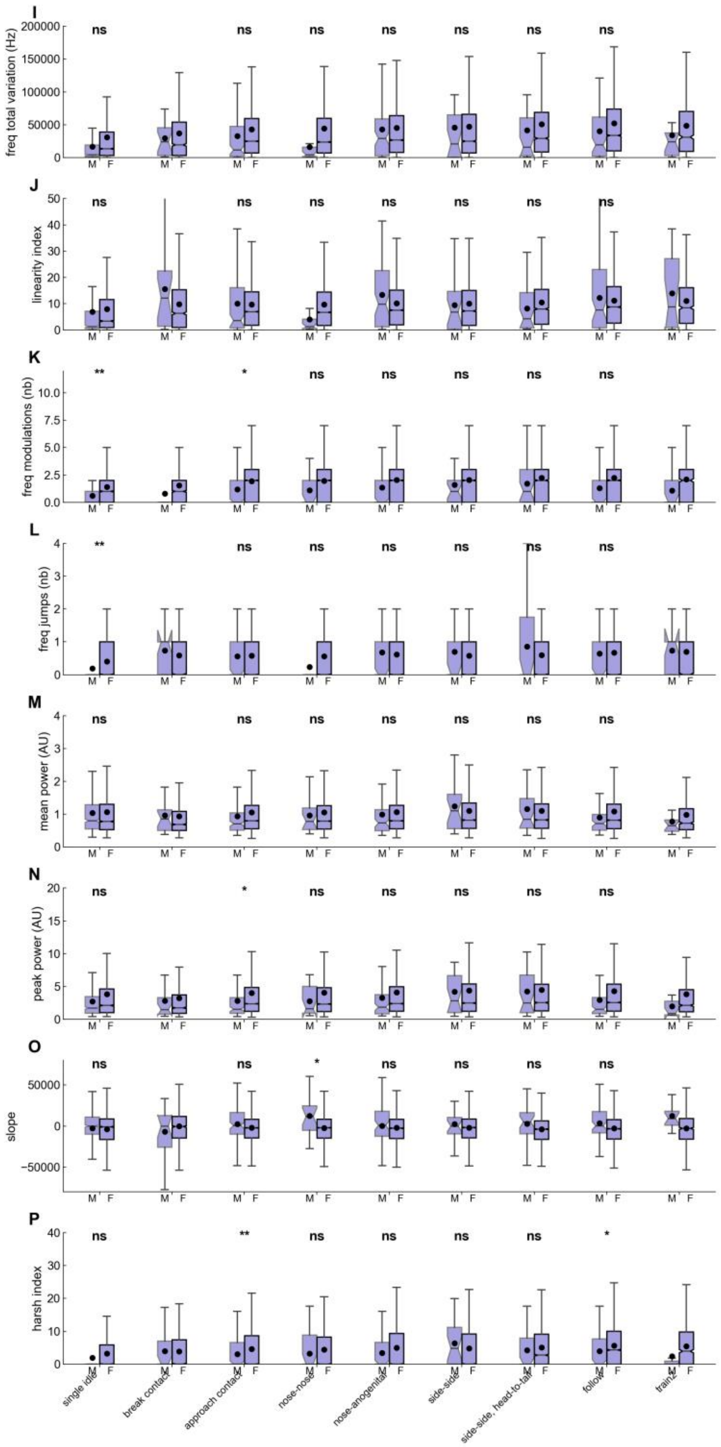

Supplementary Figure S12. Sex-related variations in acoustic features across contexts in B6 mice aged 3 months. Linear mixed model with sex as fixed factor and pair as random factor; ns: not significant; uncorrected p-values: *: $p < 0.05$, **: $p < 0.01$, ***: $p < 0.001$. P-values followed by ° survived after correction for multiple testing over acoustic variables and contexts.

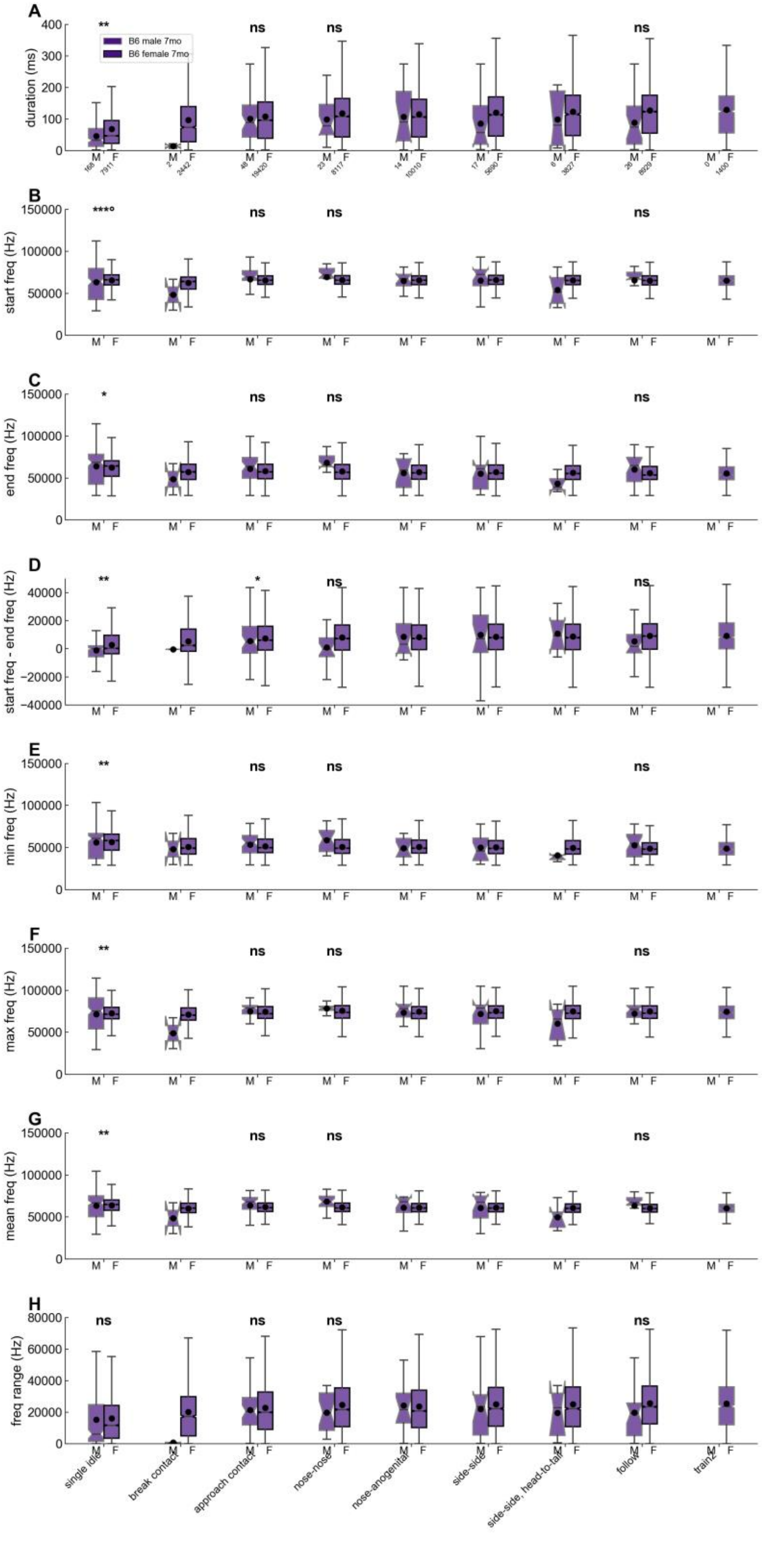
A
duration (ms)
B6 male 7mo
B6 female 7mo
B
start freq (Hz)
C
end freq (Hz)
D
start freq - end freq (Hz)
E
min freq (Hz)
F
max freq (Hz)
G
mean freq (Hz)
H
freq range (Hz)
single idle
break contact
approach contact
nose-nose
nose-anogenital
side-side
side-side, head-to-tail
follow
train2

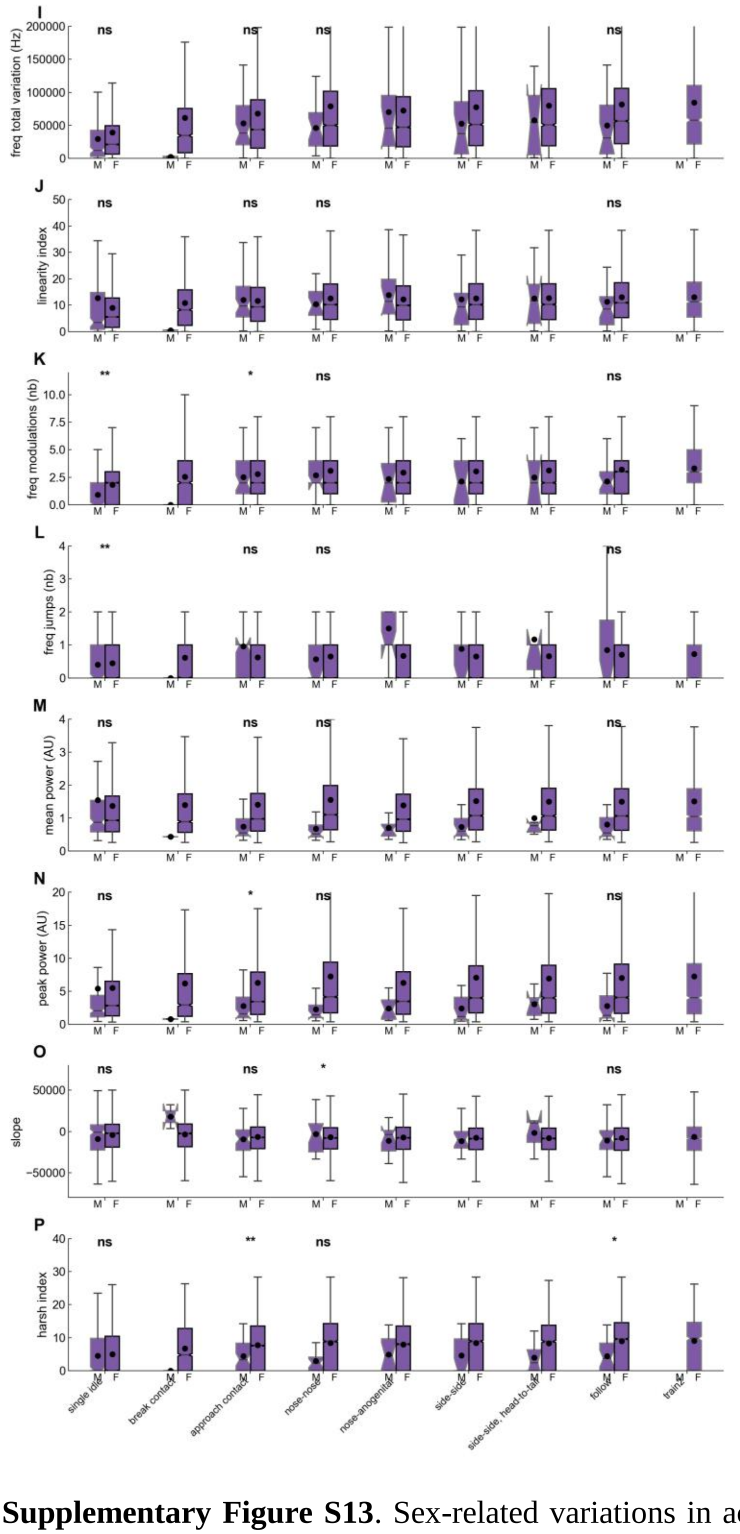

Supplementary Figure S13. Sex-related variations in acoustic features across contexts in B6 mice aged 7 months. Linear mixed model with sex as fixed factor and pair as random factor; ns: not significant; uncorrected p-values: *: $p < 0.05$, **: $p < 0.01$, ***: $p < 0.001$. P-values followed by ° survived after correction for multiple testing over acoustic variables and contexts.

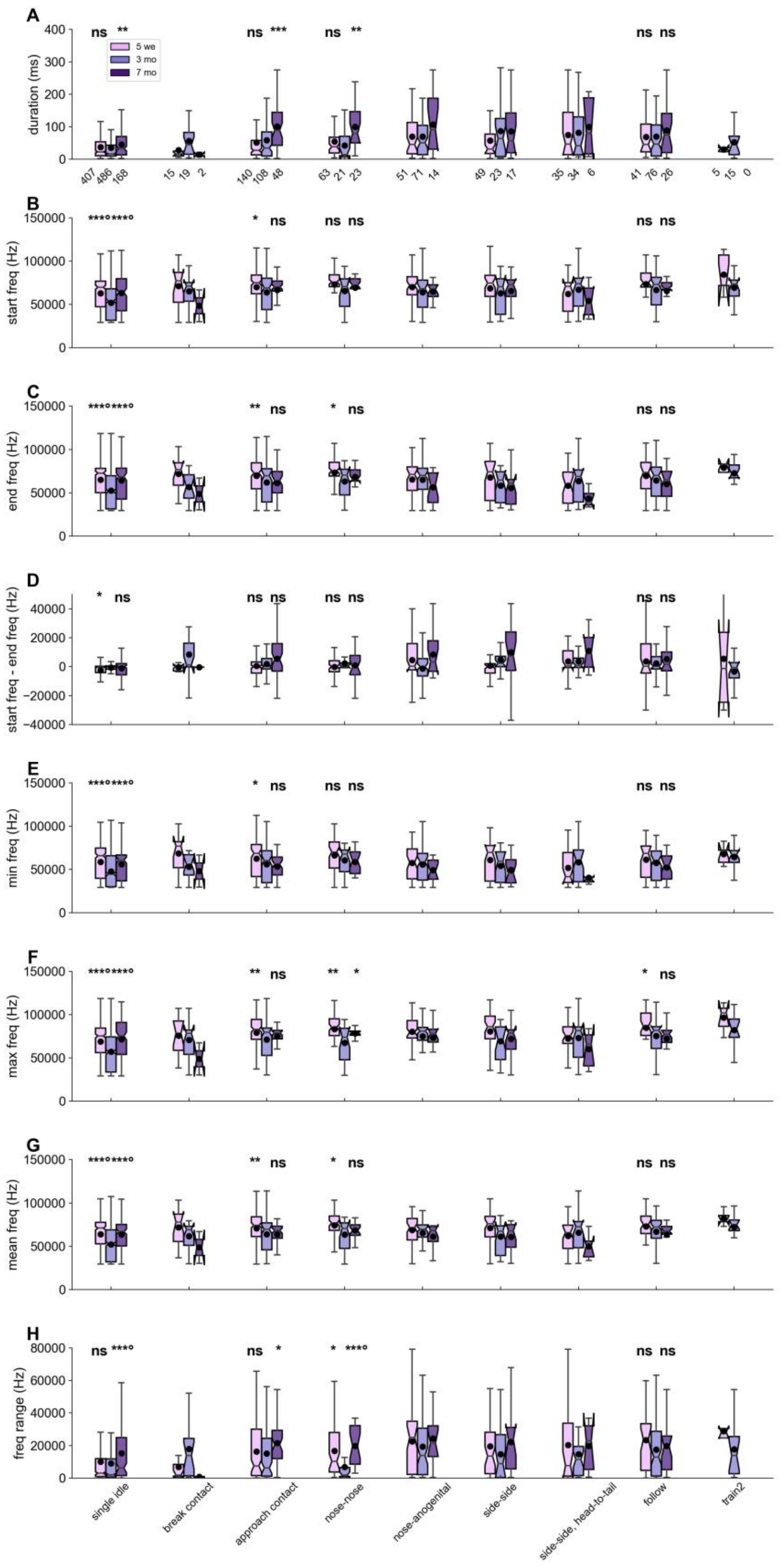

A
B
C
D
E
F
G
H
duration (ms)
start freq (Hz)
end freq (Hz)
start freq - end freq (Hz)
min freq (Hz)
max freq (Hz)
mean freq (Hz)
freq range (Hz)
5 we
3 mo
7 mo
single idle
break contact
approach contact
nose-nose
nose-anogenital
side-side
side-side, head-to-tail
follow
train2

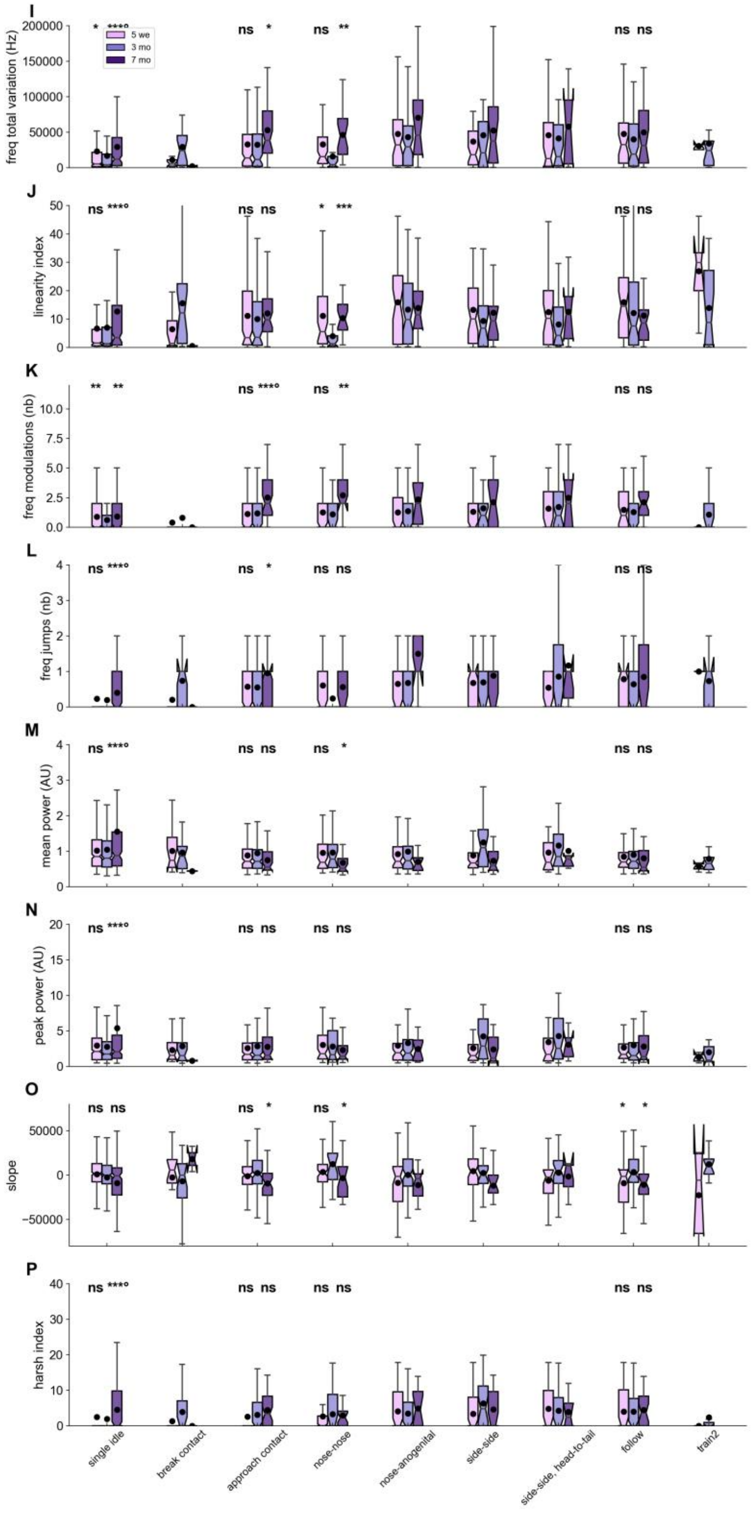

Supplementary Figure S14. Age-related variations in acoustic features across contexts in B6 males recorded at 5 weeks, 3 months and 7 months of age. Student's T-tests between 5 weeks and 3 months and between 3 months and 7 months; ns: not significant; uncorrected p-values: *: $p < 0.05$, **: $p < 0.01$, ***: $p < 0.001$. P-values followed by ° survived after correction for multiple testing with two tests over acoustic variables and contexts.

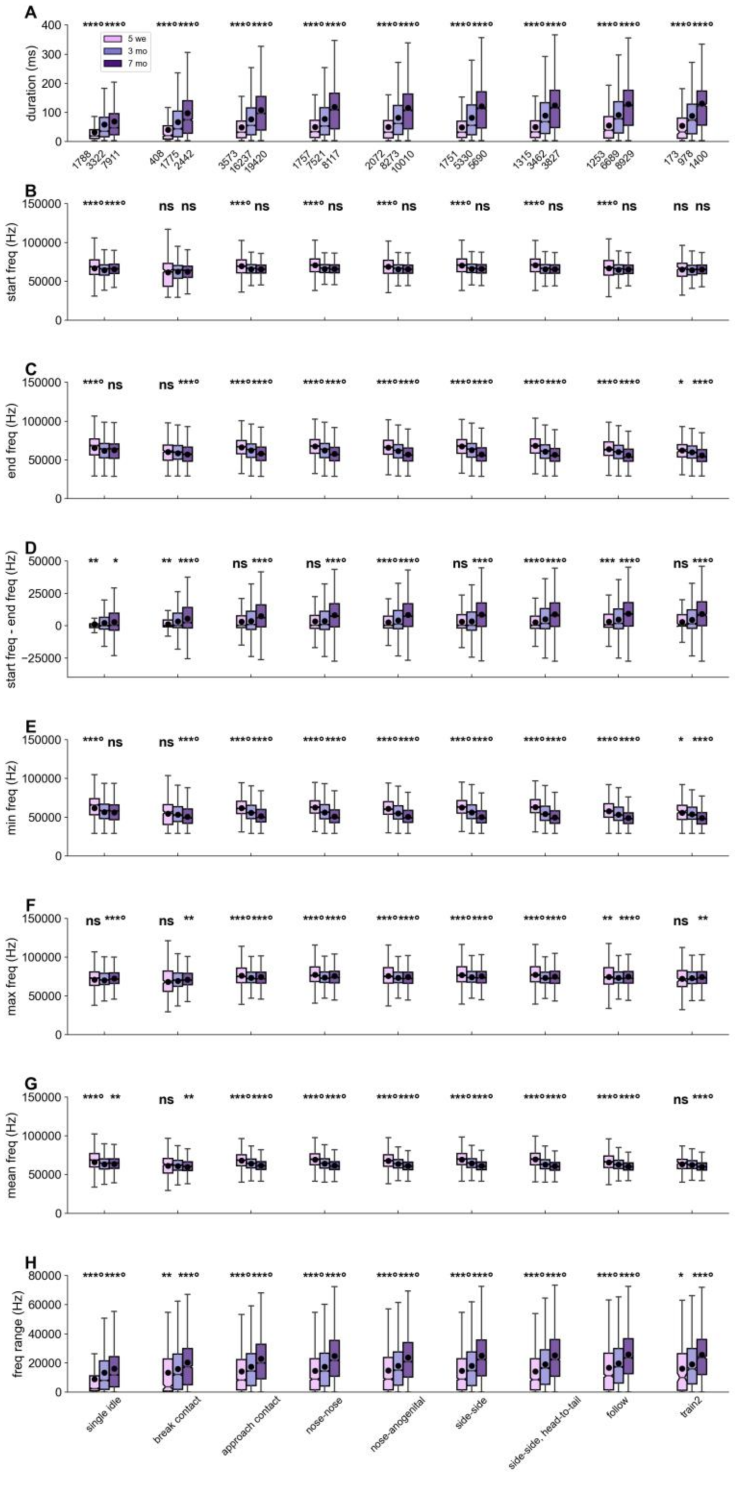
A
5 we
3 mo
7 mo
duration (ms)
1788 3322 7911
408 1775 2442
3573 16237 19420
1757 7521 8117
2072 8273 10010
1751 5330 5690
1315 3462 3827
1253 6689 8929
173 978 1400
B
start freq (Hz)
C
end freq (Hz)
D
start freq - end freq (Hz)
E
min freq (Hz)
F
max freq (Hz)
G
mean freq (Hz)
H
freq range (Hz)
single idle
break contact
approach contact
nose-nose
nose-anogenital
side-side
side-side, head-to-tail
follow
train2

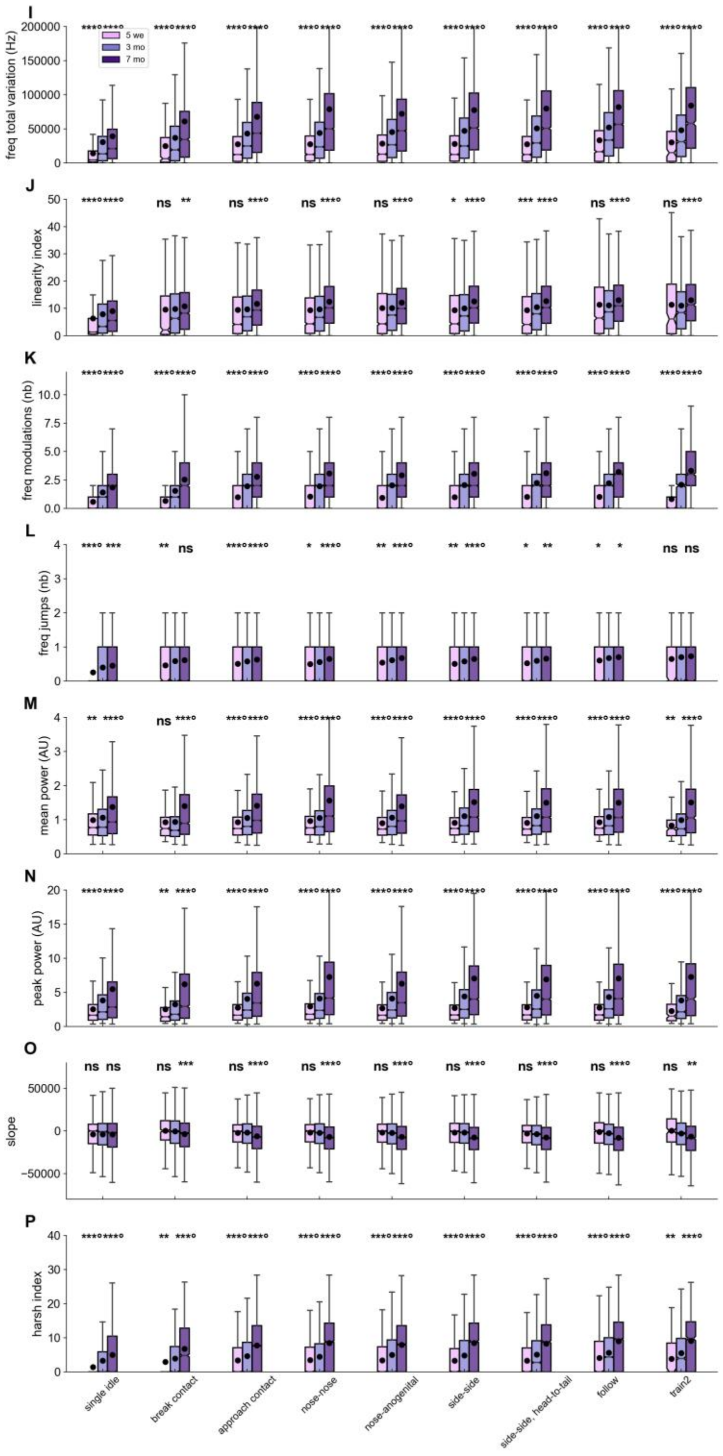

Supplementary Figure S15. Age-related variations in acoustic features across contexts in B6 females recorded at 5 weeks, 3 months and 7 months of age. Student's T-tests between 5 weeks and 3 months and between 3 months and 7 months; ns: not significant; uncorrected p-values: *: $p < 0.05$, **: $p < 0.01$, ***: $p < 0.001$. P-values followed by ° survived after correction for multiple testing with two tests over acoustic variables and contexts.

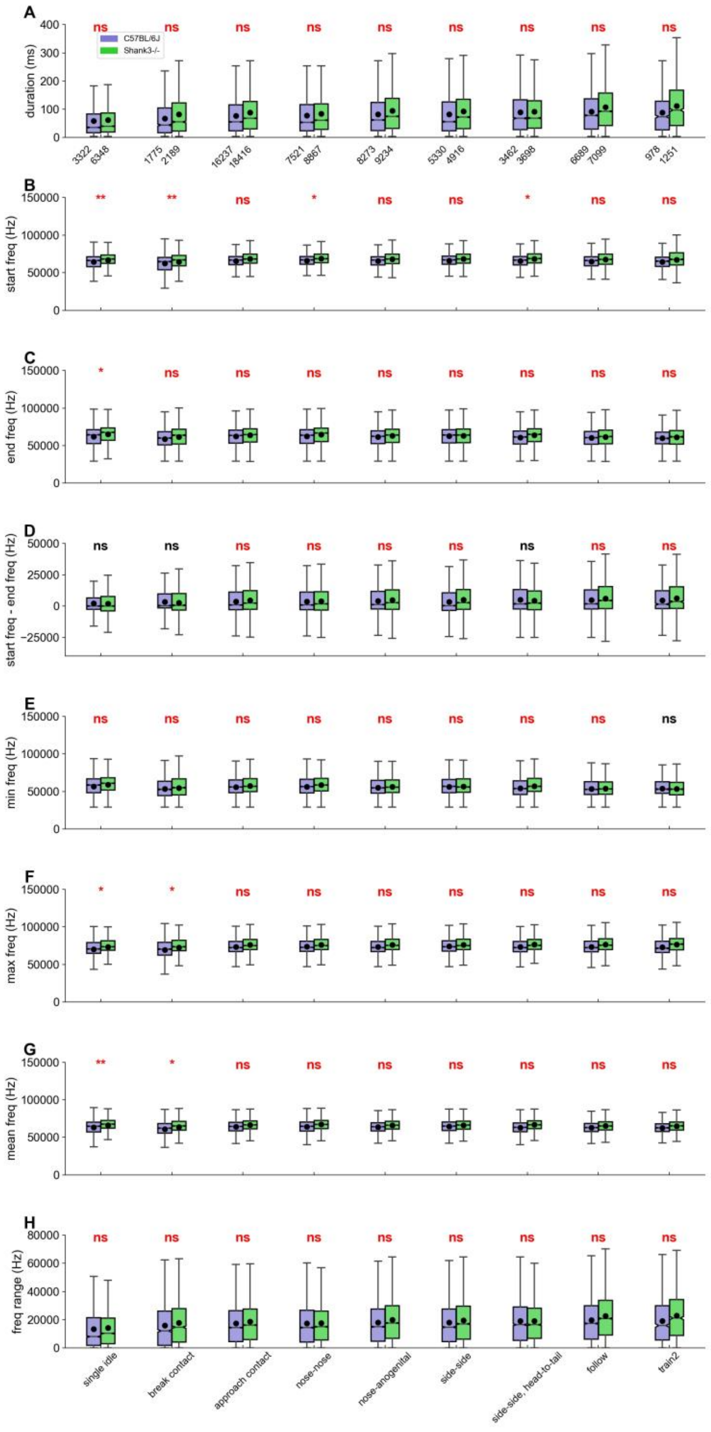
A
B
C
D
E
F
G
H
C57BL/6J
Shank3-/-
duration (ms)
start freq (Hz)
end freq (Hz)
start freq - end freq (Hz)
min freq (Hz)
max freq (Hz)
mean freq (Hz)
freq range (Hz)
3322 6348
1775 2189
16237 18416
7521 8867
8273 9234
5330 4916
3462 3698
6689 7099
978 1251
single idle
break contact
approach contact
nose-nose
nose-anogenital
side-side
side-side, head-to-tail
follow
train2

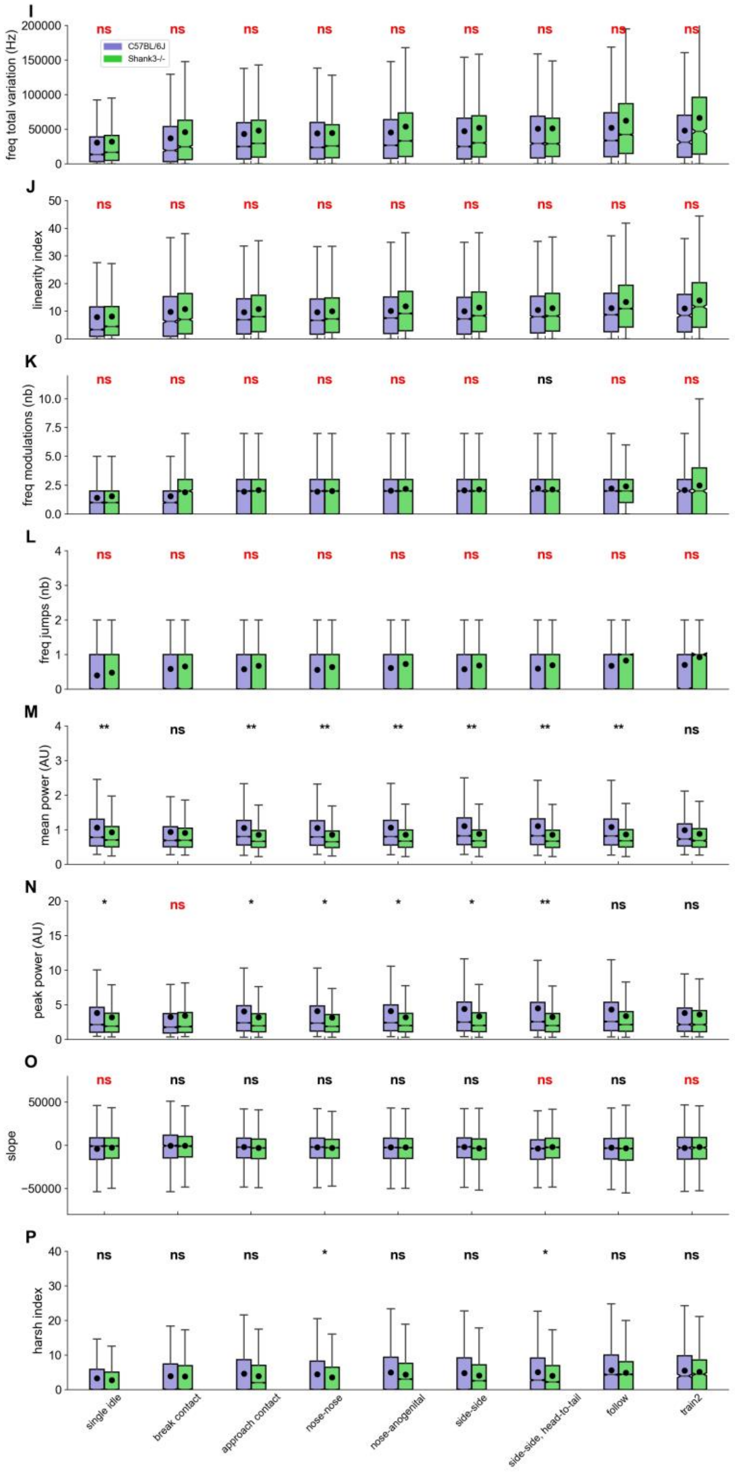

Supplementary Figure S16. Comparisons of the acoustic structure across contexts between B6 and *Shank3*$^{-/-}$ females. Linear mixed model with genotype as fixed factor and pair as random factor; ns: not significant; uncorrected p-values: *: $p < 0.05$, **: $p < 0.01$, ***: $p < 0.001$. P-values followed by ° survived after correction for multiple testing over acoustic variables and contexts (in black: values in B6 females are higher than in *Shank3*$^{-/-}$ females; in red: values in *Shank3*$^{-/-}$ females are higher than in B6 females).

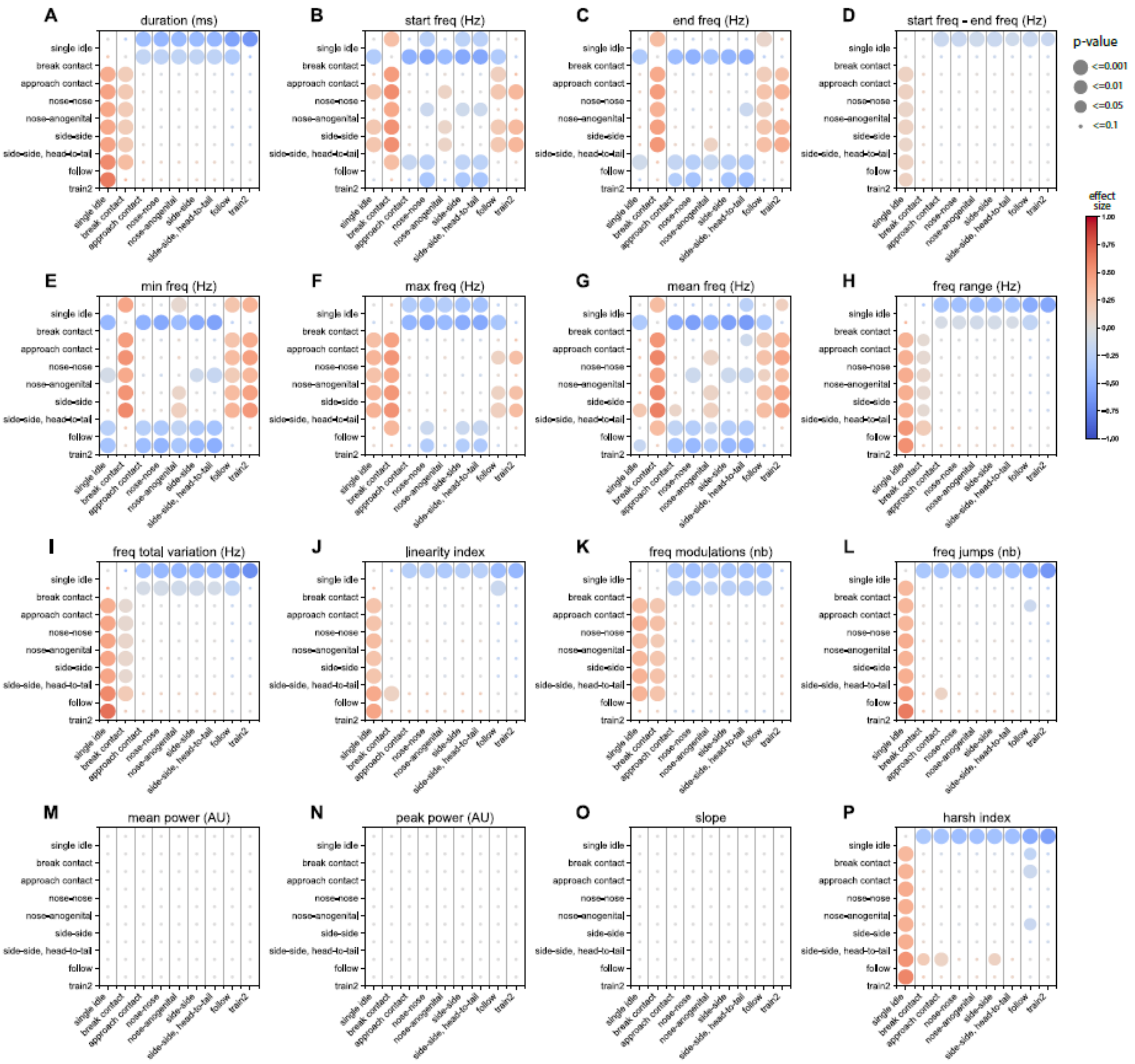

Supplementary Figure S17. Variations of the acoustic features of USVs emitted in different behavioral contexts in 5-week-old pairs of female B6 mice. All acoustic traits measured for each USV are depicted. (Linear Mixed Models: fixed factor=context, random factor=pair). Blue colors indicate that the acoustic feature of USVs given during y-event are lower than the acoustic feature of USVs given in x-event; red colors indicate that the acoustic feature of USVs given during y-event are higher than the acoustic feature of USVs given in x-event; the effect size is represented by the color scale while the significance levels are represented by the size of the circles.

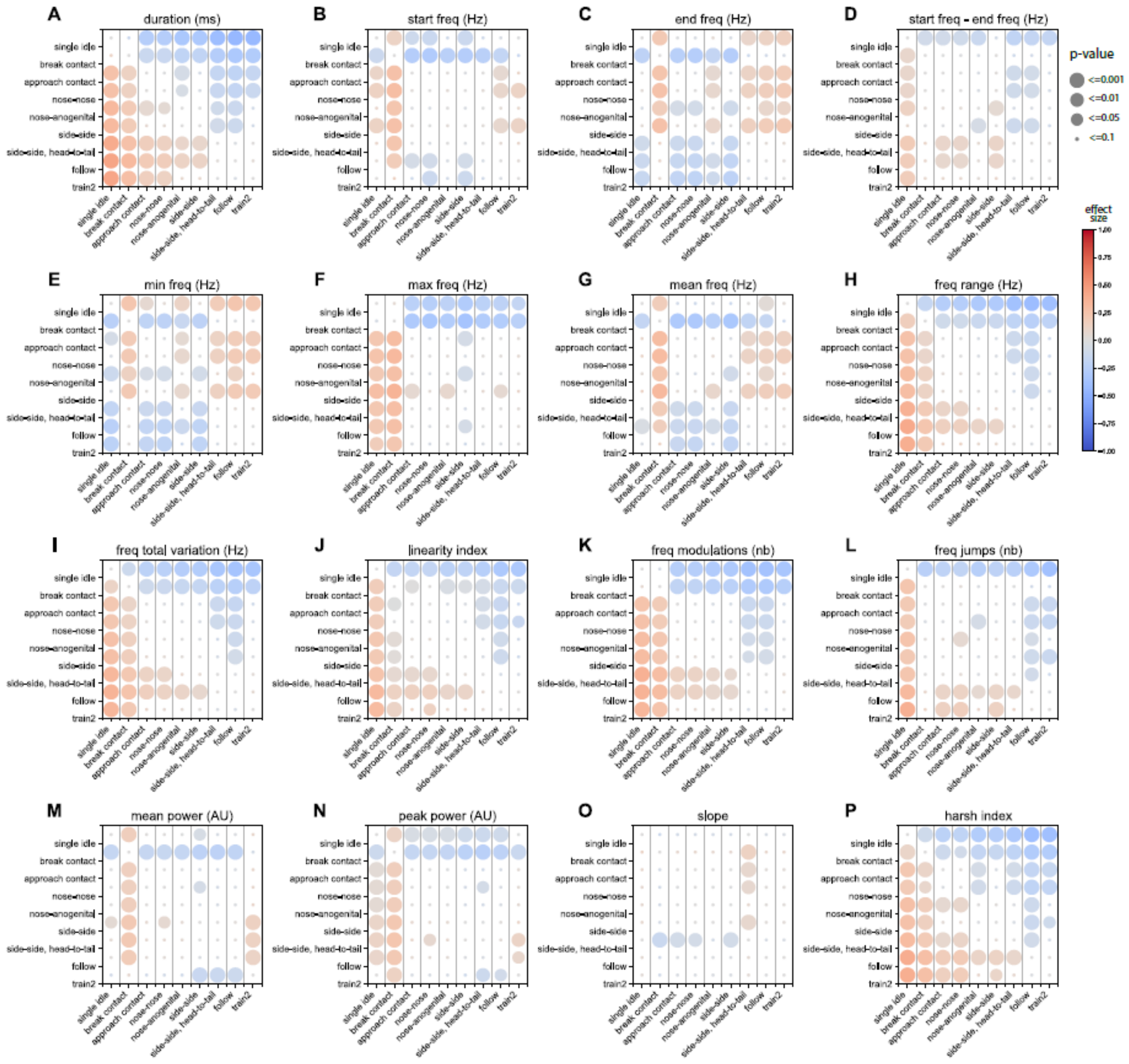

Supplementary Figure S18. Variations of the acoustic features of USVs emitted in different behavioral contexts in 3-months-old pairs of female B6 mice. All acoustic traits measured for each USV are depicted. (Linear Mixed Models: fixed factor=context, random factor=pair). Blue colors indicate that the acoustic feature of USVs given during y-event are lower than the acoustic feature of USVs given in x-event; red colors indicate that the acoustic feature of USVs given during y-event are higher than the acoustic feature of USVs given in x-event; the effect size is represented by the color scale while the significance levels are represented by the size of the circles.

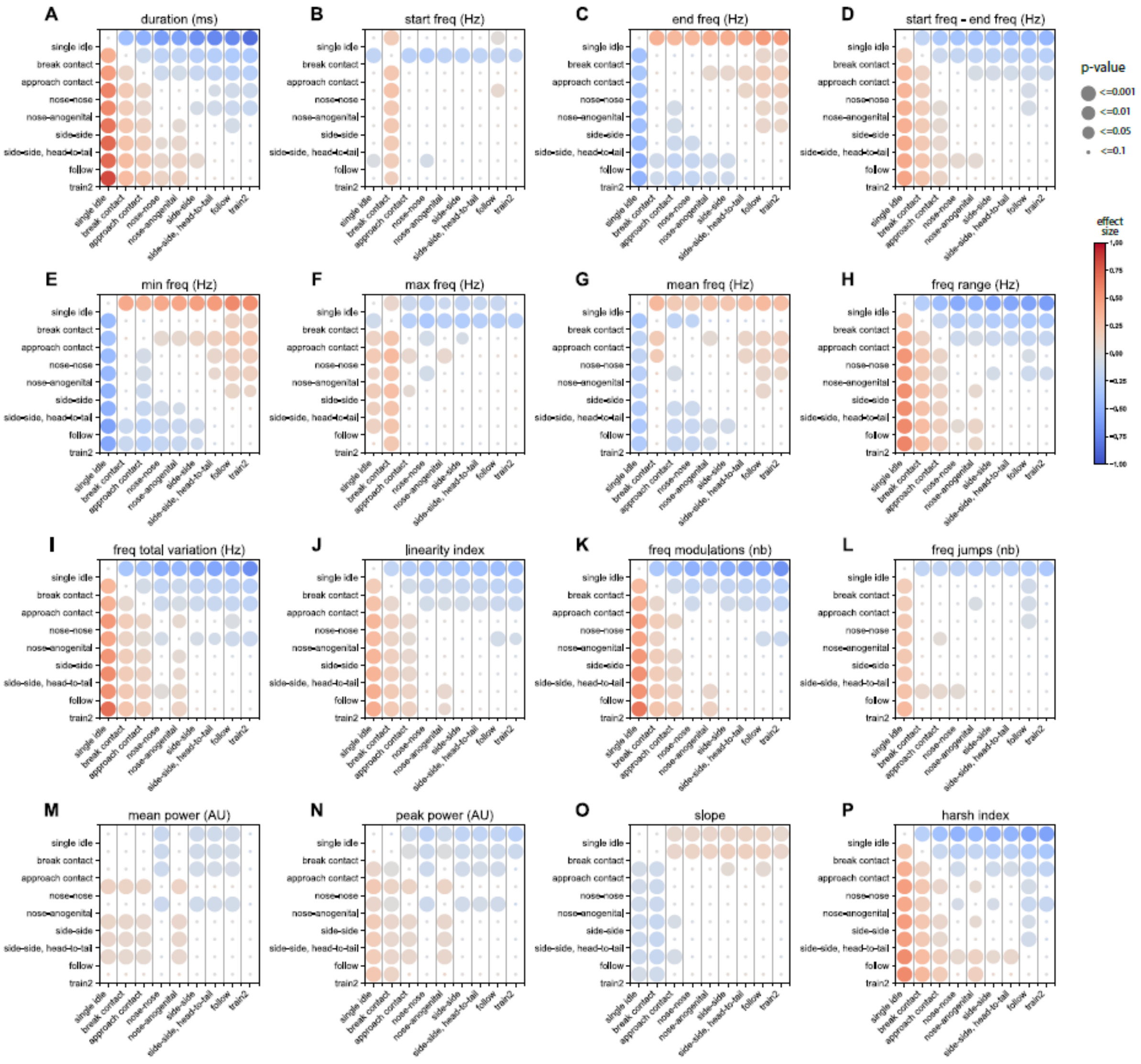

Supplementary Figure S19. Variations of the acoustic features of USVs emitted in different behavioral contexts in 7-months-old pairs of female B6 mice. All acoustic traits measured for each USV are depicted. (Linear Mixed Models: fixed factor=context, random factor=pair). Blue colors indicate that the acoustic feature of USVs given during y-event are lower than the acoustic feature of USVs given in x-event; red colors indicate that the acoustic feature of USVs given during y-event are higher than the acoustic feature of USVs given in x-event; the effect size is represented by the color scale while the significance levels are represented by the size of the circles.

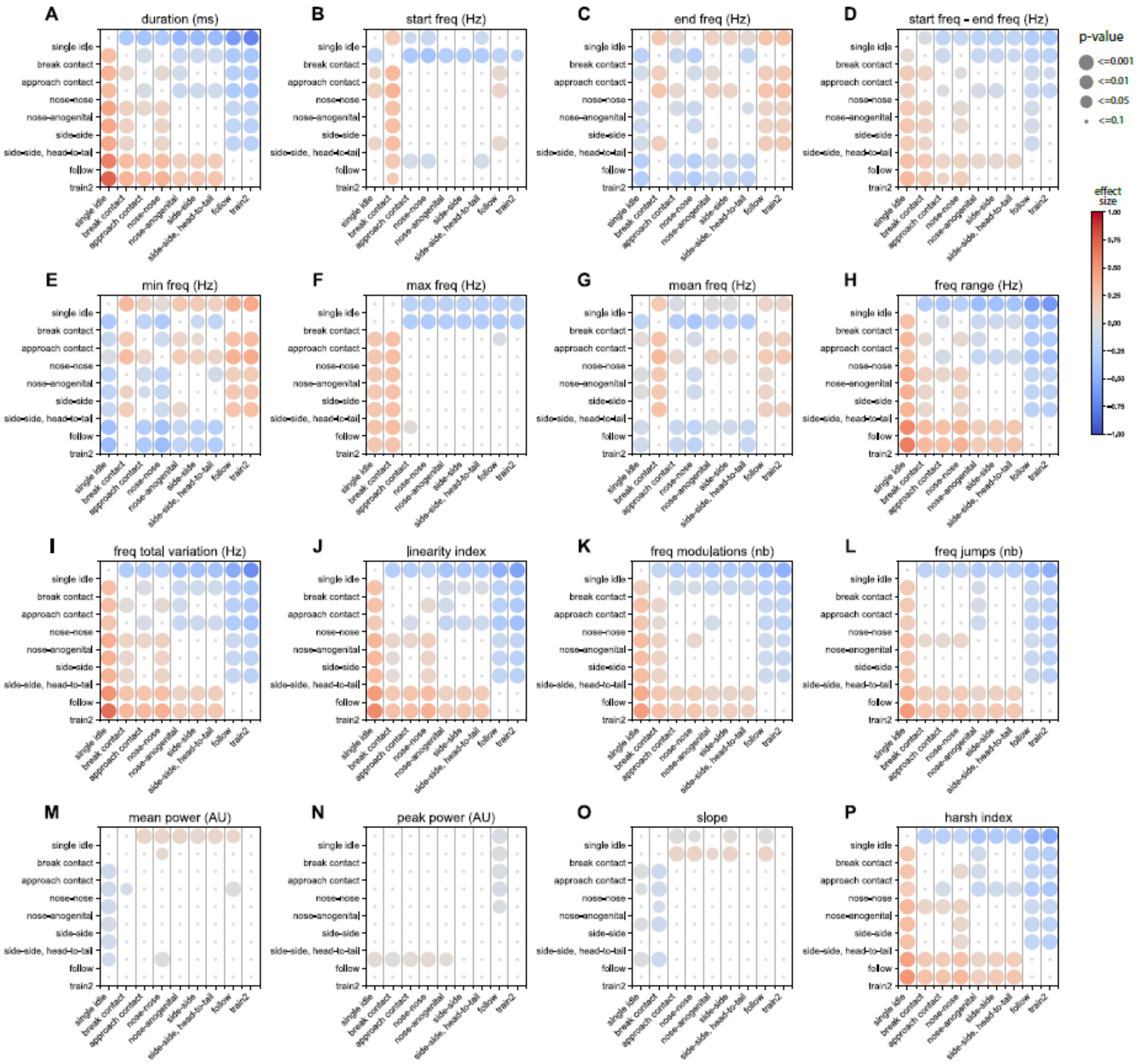

Supplementary Figure S20. Variations of the acoustic features of USVs emitted in different behavioral contexts in 3-months-old pairs of female *Shank3*$^{-/-}$ mice. All acoustic traits measured for each USV are depicted. (Linear Mixed Models: fixed factor=context, random factor=pair). Blue colors indicate that the acoustic feature of USVs given during y-event are lower than the acoustic feature of USVs given in x-event; red colors indicate that the acoustic feature of USVs given during y-event are higher than the acoustic feature of USVs given in x-event; the effect size is represented by the color scale while the significance levels are represented by the size of the circles.

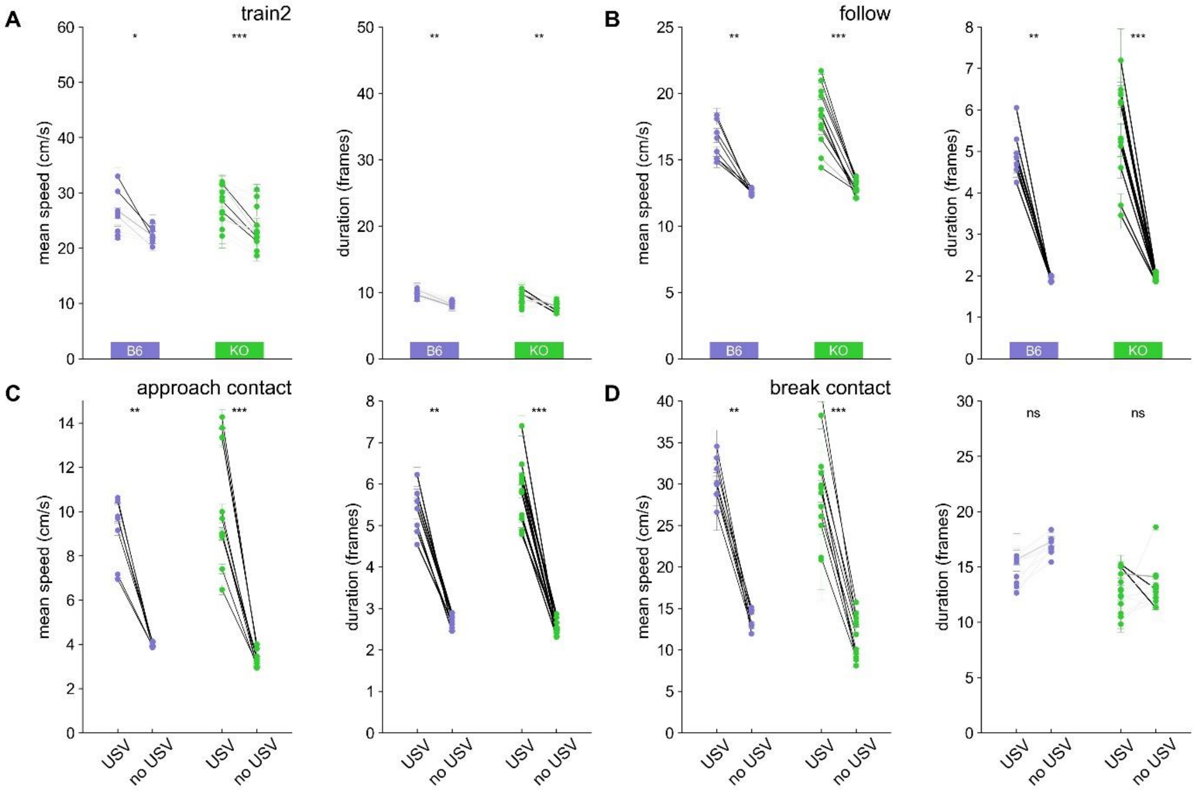

Supplementary Figure S21. Variations in mean speed and duration of each behavioral event according to the presence or absence of USVs in B6 and *Shank3*$^{-/-}$ females aged 3 months. Variations of the mean speed of the animal performing the behavior (left panel) and of the event duration (right panel) for (A) train2, (B) follow, (C) approach contact and (D) break contact. Significant differences using Mann-Whitney U-tests within each individual after Bonferroni correction (24 tests conducted for each behavioral event and variable tested) are depicted by the color of the segments linking the mean value with and without USVs: light grey: not significant, medium grey: adjusted p<0.05, dark grey: adjusted p<0.01, black: adjusted p<0.001.

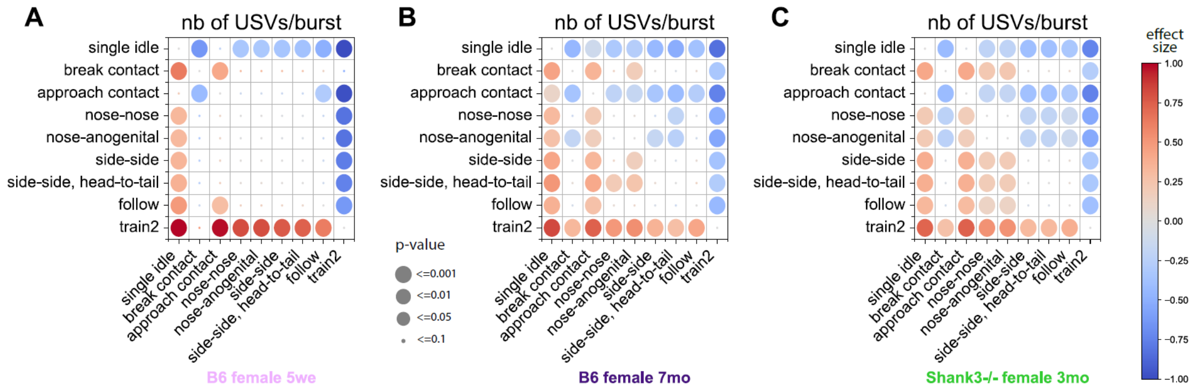

Supplementary Figure S22. Number of USVs per USV bursts across the different contexts of emission in females B6 aged 5 weeks and 7 months as well as in female *Shank3*$^{-/-}$ aged 3 months. (Linear Mixed Models with context as fixed factor and pair as random factor). Blue colors indicate that the number of USVs per burst given during y-event are lower than the number of USVs per burst given in x-event; red colors indicate that the number of USVs per burst given during y-event are higher than the number of USVs per burst given in x-event; the effect size is represented by the color scale while the significance levels are represented by the size of the circles.

Supplementary Tables

Supplementary Table I. Validation scores by comparison with manual annotation of USVs.

Sequence type	Number of USVs (Ground Truth)	False positive	False negative (Missed)	True Positive	Precision (nb TP / (nbTP+nbFP))	Recall (nbTP / nbGT)
5 weeks females	554	107	137	417	0.79	0.75
3 months females	982	105	172	810	0.88	0.82
7 months females	1384	296	310	1074	0.78	0.77
5 weeks males	162	52	32	130	0.71	0.8
3 months males	132	48	44	88	0.64	0.66

Supplementary Table II. Description of the acoustic variables measured for each USV

Trait list for USV	
Trait<br>(codename)	Description
duration	Duration of the USV, in millisecond.
frequencyDynamicHz	Difference between the maximum and the minimum peak frequency of the USV.
startFrequencyHz	Peak frequency at the beginning of the USV
endFrequencyHZ	Peak frequency at the end of the USV
diffStartEndFrequencyHz	endFrequencyHZ - startFrequencyHz
minFrequencyHz	Minimum peak frequency
maxFrequencyHz	Maximum peak frequency
meanFrequencyHz	Mean of the peak frequencies over each t in the USV
frequencyTVHz	Total variation of the peak frequency for each t (sum of the absolute values of the difference between consecutive peak frequencies).
meanFrequencyTVHz	FrequencyTVHz divided by the number of points in the USV.
linearityIndex	Sum of the euclidean distance to the linear regression divided by the number of points in the USV.
meanPower	Mean of the power for the USV

nbModulation	Number of times where the signal gets over and below the linear regression of the signal. Note that the signal should overtake linear regression by 876Hz (which corresponds to 3 times the frequency accuracy of the 1024 FFT)
Harsh index	This index reflects the frequency width of the peak signal. We compute a power threshold over which frequencies are considered. Then we measure the number of adjacent frequencies over this threshold. The result is normalized by the duration of the USV. Code is available in ComputeHarshIndex.py, on the gitHub repository.
slope	Difference between the last and the first frequency of the linear regression of the signal.
slopeNormalized	Corresponds to slope / durationMs
nbJump	The number of jumps is computed if the frequency dynamic of the USV is over 10 kHz. Then if the frequency changes from one t to the next over more than ⅓ of the frequency dynamic, we set one jump.

Supplementary Table III. Description of the characteristics for each USV burst

Trait list for USV Burst	
Trait	Description
nbUSV	Number of USVs contained in the burst
durationMs	Duration of the burst, in millisecond.
meanDuration	Mean duration of USVs contained in the burst
stdDuration	Standard deviation of the durations of USVs contained in the burst
meanInterval	Mean interval (silence duration) between the USVs
meanFrequency	Mean of mean peak frequency of USVs contained in the burst
mean power	Mean power of USVs contained in the burst

Supplementary Methods

1 Motivation to create a new recording and analysis pipeline

To detect and analyze USVs within background noise while monitoring the behaviors, automation was needed to handle large data sets, reduce processing time and avoid variability in human-generated errors. Existing systems (listed on the MouseTube website; (Torquet et al., 2016)) did not completely fulfil our needs. Indeed, some systems provide a classification of USV types without automated USV detection (e.g., VoiCE, (Burkett et al., 2015)). Other systems provide both automatic detection of USVs and extraction of acoustic features (A-MUD, (Zala et al., 2017); USVSEG, (Tachibana et al., 2020); Ax, (Seagraves et al., 2016)). Some even classify USVs into call types, either in a pre-determined repertoire (Mouse Song Analyzer v1.3, (Arriaga et al., 2012; Chabout et al., 2015)) or in an open repertoire determined by the data themselves (MUSE, (Neunuebel et al., 2015); MUPET, (Van Segbroeck et al., 2017); DeepSqueak, (Coffey et al., 2019)). Nevertheless, most of these systems do not handle background noise, and only MUSE provides the synchronization with behavioral monitoring, along with a heavy triangulation system that could not be easily replicated and adapted to our Live Mouse Tracker behavioral monitoring system.

2 USV Segmentation Method

2.1 Method parameters

We aimed to create a method with a minimum of parameters. Nevertheless, we still have one: is the vocalization emitted by an adult or a pup? Indeed, the major difference is the recording protocol. Pups are recorded at a close range, with low environmental noise. Juvenile and adult mice are recorded with the microphone placed at a distance to cover the surface of the test cage. Also, as they move freely, noise generated by their interactions with the environment is important. Therefore, as the signal to noise ratio is better in pup recordings than in adult conditions, we lower our detection threshold for pups. In addition, as the range of emission of pups rises up to 140 kHz, we increase the maximum frequency allowed for detection.

2.2 Filtering spectrum data

For each step, we provide pseudo code. For an easier reading, we removed the boundary check conditions that exists in the real code.

For each time point of the spectrum, we center the data by removing the mean of all magnitudes for all frequencies at the current time point.

```
for each timePoint in spectrogram

        mean_magnitude_for_current_T = mean ( spectrum[t] )

        for each frequency in spectrogram

                spectrum[timePoint][frequency] -= mean_magnitude_for_current_T
```

We then use a filter that creates continuity in the signal. It basically fills the gap in the signal if a direction is found in the spectrum's curves. To perform this filtering, we first create a filter bank for a number of angles. The following code pre-computes the filters for the different orientations:

```
filterWidth = 5

filterHeight = 1

filterList = List

for angle from -80 to 80 with a step of 20

        filter = createFilter()

        for width from -filterWidth to filterWidth

                for thick from -filterHeight to filterHeight

                        offset_x = cos ( angle ) * width

                        offset_y = sin ( angle ) * width

                        offset_x+= cos ( angle + 90 ) * thick

                        offset_y+= sin ( angle + 90 ) * thick

                        filter.storePoint( x , y )

        filterList.add( filter )
```

Then, we apply those pre-computed filters on each point of the spectrogram, and we keep the maximum response. This code is parallelized for each time point for maximum performance.

```
for each timePoint in spectrogram

        for each frequency in spectrogram

                max = -infinite

                for filter in filterList:

                        val = 0

                        for offset_point in filter:

                                val+= spectrum[ t+offset_point.x ][ f+offset_point.y ]

                if val > max:

                        max = val

                spectrumSmoothed[t][f] = max / numberOfPointInFilter
```

We then filter the vertical signal to remove noise (seen as strong vertical scratches in the spectrum). The following pseudo code removes for each frequency the local mean frequency of the spectrum, using a sliding window of +/- 1.5 kHz.

```
frequencyWindow = 10 // The window frequency is then ((10*2)+1)*(300000/1024*2) = 3076Hz

for each timePoint in spectrogram

        result = List

        for each frequency in spectrogram

                sum = 0

                for offsetFrequency from -frequencyWindow to frequencyWindow

                        sum+=spectrum[timePoint][frequency+offsetFrequency]

                mean = sum / (frequencyWindow *2+1)

                result[frequency] = mean


        for each frequency in spectrogram

                spectrum[timePoint][frequency] -= result[frequency]
```

2.3 Constant and blinking frequency canceler

In the experiments, we observed noise due to light, fan, power sources and air-conditioning. They appear and disappear randomly during experiment, at unpredictable frequencies, and can switch in frequency. We process the spectrum to find the frequencies of those noise to store them in a “frequency cancelation list”.

```
detectionThreshold= 0.1f

minFrequencyConsidered = 100 // ( 30kHz)

maxFrequencyConsidered = 512-100 // (120kHz)


if vocs are from pups

        maxFrequencyConsidered = 512 // (150kHz)

        detectionThreshold = 0.05


valueList = List

for each frequency from 100 (30kHz) to 512-100 (120kHz) in spectrogram
```

```
        for each timePoint in spectrogram
                valueList.append( spectrum[timePoint][frequency] )
mean = mean ( valueList )
std = standardDeviation( valueList )

threshold= mean+0.15*std
cancelFrequencyList = List
for each frequency from minFrequencyConsidered to maxFrequencyConsidered
        nbValOver = 0
        for each timePoint in spectrogram
                val = spectrum[timePoint][frequency]
                if val > threshold
                        nbValOver+=1
                if nbValOver > number of time point * 0.4:
                        cancelFrequencyList.append( frequency )

for frequencyCanceled in cancelFrequencyList
        for each timePoint in spectrogram
                spectrum[timePoint][frequencyCanceled] = 0
```

2.4 Vocalization segmentation

On the filtered signal, we now consider all values over 0 in spectrum.

```
// remove vertical noise
for ( int x = 0 ; x < width ; x++ )
{
        double sum = 0;
        for ( int y = 0 ; y < height ; y++ )
        {
                double val = buffer[x+ ( y )*width ];
                sum+=val;
```

```
                }
                double m = sum/height;
                for ( int y = 0 ; y < height ; y++ )
                {
                        buffer[x+ ( y )*width ]-=m*20d;
                }
        }

for each frequency from spectrum
        for each timePoint in spectrogram
                if spectrum[timePoint][frequency] < threshold
                        spectrum[timePoint][frequency] = 0

maskList = perform connected component detection
filteredMask = List

for mask in maskList:
        nbPoint = mask.nbPoint
        nbInSameTimePoint = getHowManyMaskAtSameTimePoint( mask, maskList )
        meanStdOfMask = getMeanStd( mask, spectrum )

        status = unknown
        if meanSTD < 0.15:
                status = rejected
        if nbPoint < 5:
                status = rejected
        if meanSTD > 1
                status = accepted
        if meanSTD > 0.15 and meanSTD < 1 and nbPoint > 50:
                status = accepted
        if nbInSameVertical==0 and status is not rejected
                status = accepted
```

```
        if nbInSameVertical > 3 and nbPoint < 150
                status = rejected
        if status == accepted
                keptMaskList.append( mask )
```

Then masks are merged together if they are sharing a time point. They are then temporally merged again if the silence between the signals is below 40 ms.

2.5 Spectrum signal extraction

The final extraction of the signal is the maximum magnitude per time point that belongs to the mask of the time point (if available).

```
vocList = List
for each mask in fusedMaskList
        voc = new Voc
        for t from mask.startT to mask.endT
                maxMagnitude = -infinity
                bestFound = False
                maxFrequency = 0
                for frequency in spectrum
                        if mask.contains( t , frequency )
                                value = spectrum[t][frequency]
                                if value > maxMagnitude
                                        maxMagnitude = 0
                                        bestFrequency = frequency
                                        bestFound = True
                if bestFound
                        voc.addPoint( t , frequency )
        if voc.nbPoint > 0
                vocList.append( voc )
```

2.6 USV Detection validation

To perform the validation, we consider the beginning and the end of each USV. We run our segmentation algorithm on a set of manually annotated USVs (10 files for each experiment). If the USV boundaries match at +/- 40 ms, we consider that the USV has been correctly detected (**Supplementary Table I**).

Nevertheless, this metric is based on the start and end time of the sequences, which raises two concerns:

- After the processing, we checked again the ground truth. We found that most discrepancies between the ground truth and the automatic segmentation emerge from the merging of two parts of USV connected by a low-power signal in the manual annotation, while the two parts were considered separately by the automatic segmentation. In that case, the penalty is very high, as this leads to two false positives and one false negative.
- The second concern is that our metric does not check if we segment correctly the peak frequency itself. This would have required the development of a dedicated annotation tool. Nevertheless, we believe that such method should be introduced in our further developments.

3 Filtering out wave files containing only noise

During an experiment, we do not record continuously the audio information. We use Avisoft-RECORDER automatic triggering capability which monitors the audio and starts the recording when a predetermined power threshold is reached. Therefore, the dataset of an experiment is composed of thousands of files that may contain USVs or just noise due to the activity within the cage. Therefore, data of an experiment can be preprocessed to filter out wave files containing only noise, which represent 50% of the files generated in our experiment.

An expert sorted files containing USVs from files containing noise to train a random forest classifier. We use the following features as machine learning features: for each file, we extract the mean power of the whole file, the number of presumed USVs detected, the duration of the USVs over the overall length of the file, the average duration of USV and its standard deviation, the mean peak frequency and the standard deviation of the peak frequency.

We then train the random forest classifier. We provided a pre-trained classifier but one can re-train the classifier with its own data. One just needs to start the python script and point two different folders (noise and USVs) to train the system. For our experiments, we trained the system with 451 files containing USVs and 247 with only noise. The accuracy of the training is 97%, using 10 folds.

Then, the system can be used in predictive mode to sort dataset containing both USVs and noise. The script copies the file in a noise and USV folder, so that the user can easily control the sorting accuracy.

3.1 Avisoft record and synchronization with Live Mouse Tracker

The system is designed to work for an unlimited duration. As USVs are infrequent events, we do not record the sound continuously. We instead use the automatic record trigger functionality of Avisoft-RECORDER. The automatic trigger of Avisoft-RECORDER monitors the sound level and start

recording a sound if the current sound level within a given frequency range is over a given threshold. The sound is recorded as long as the sound level is over the threshold. This function takes a hold time parameter: if Avisoft-RECORDER detects another signal during the hold period, the record is not interrupted. The hold period also adds a record period around the first and the last signal over threshold. In our experiment, we use a hold time of one second.

To synchronize USV recording with the tracking, we use the “Trigger control” of Avisoft-RECORDER. This function allows to launch an external program at each start and end of records. We use the free software PacketSender (https://packetsender.com/) to perform communication between Avisoft-RECORDER and Live Mouse Tracker (LMT). Through PacketSender, we send an UDP string packet containing the file number currently recorded by Avisoft-RECORDER. This information is recorded by LMT within the database as an “USV event”. The goal of the synchronization is to match the USV record with the current data frame number recorded by LMT.

3.2 USV Toolbox, an open-source, free and online USV analysis pipeline

The currently available methods to detect and analyze mouse USVs need specific installations and software. To facilitate the testing of our own algorithm, we provide a website to test the method or to process data online: https://usv.pasteur.cloud. The user simply drags and drops his/her wave file, waits a few seconds (depending on the length of the sample file) and finally evaluates the quality of the USV segmentation and the data extracted from the sound file. The goal of this website is to provide immediate access to the method without installing any software.

The first panel of the website is dedicated to evaluate USV detection. The first spectrogram represents the original data and the second one provides the annotated data. The player under this spectrogram allows to listen to the sound file slowed down by twenty times. The other panels display:

- the length of the wave file given as input.

- the number of USVs detected within the wave file.

- a timeline displaying the USVs detected over the whole file and their temporal organization in USV bursts, in which the intervals between USV are shorter than one second.

- the frequency characteristics of each USV within the sound file (in kHz). Each vertical black bar displays the min/max peak frequency of the USV (and therefore also the frequency range) while the black dot displays the mean peak frequency and the red dot displays the peak frequency with the maximum amplitude in each USV.

- the duration of each USV (in ms).

- the power (i.e., amplitude) of each USV, depicted in arbitrary unit.

- the proportion of USVs with frequency modulations.

- the proportion of USVs containing one or more frequency jump(s).

- a table gathering all acoustic variables extracted on each USV of the sound file.

The user can download all these results for his/her own sound file. These results are deleted after one hour. Data downloaded from this web page can be directly used with the scripts that we provide with the present study. To perform the analysis on thousands of files, we also provide the desktop version of the analysis program, working in batch mode (link available on https://usv.pasteur.cloud after publication).

3.3 USV analysis toolbox

As for Live Mouse Tracker, we provide an API in Python for the biologists to process USVs. This package allows one to re-create all data representations used in this study with its own data. This API is available on gitHub (will be released after publication process).

For Live Mouse Tracker, we provided a full API in Python to process event classification, and to process queries.

4 Selection of representative acoustic variables

We conducted a principal component analysis (PCA in Python with the package scikit-learn version 0.24.1) to select representative acoustic variables. The first four components explained 80% of the variance. Component 1 is represented by duration, frequency modulations and harsh index. Component 2 is represented by frequency characteristics. Component 3 is represented by power measures. Component 4 is represented by the frequency slope between the start and the end of the vocalization. Altogether, we selected duration, frequency range and harsh index as representative variables to be presented in main figures, while all other variables will be depicted in supplementary material.

5 Correlation, metrics and coefficients used in this study

- USVs or Burst versus events:

Number of frames in USV or bursts in common with a behavioral event / total number of frames of the behavioral events. This correlation reflects how much a vocal event (i.e. either USV or USV burst) is related to a behavioral event, and ponderates it by the total duration of the behavioral event. Therefore common events such as “Single idle” or “Contact” are disadvantaged as their total length is very high. Events are not exclusive, and therefore USVs or burst can be related to more than one event.

- Ratio of USVs or Burst linked to event:

Number of frames in USV or USV bursts in common with behavioral event / total number of frames of the vocal event. This ratio provides the ratio of frames correlated with an event over the total number of frames of the vocal event. With this measure, the size of the behavioral event does not matter. This measure reflects the proportion of vocal events that matches with a given behavioral event. Events are not exclusive, and therefore USVs or burst can be related to more than one event.

6 Determining intervals between USVs to define USV burst

A burst is a sequence of vocalizations. Within a burst, the silence intervals between two consecutive vocalizations do not exceed a threshold. In the literature, this threshold varies between 170 and 500 ms, depending on the contexts of recordings and the sex and strain of mice recorded (e.g., 170 ms (Hertz et al., 2020); 230 ms (Ey et al., 2013; Chabout et al., 2015); 275 ms (Castellucci et al., 2018); 500 ms (von Merten et al., 2014)). They are based on a cutoff on the distribution of intervals.

In our experiments, the distribution of intervals below 2s for all 3mo B6 Females (**Supplementary Figure SUP_USV**) has a maximum at 100-130ms. The classical method would set a threshold at 500ms, which would group 93% (19198 out of 20637) of the USVs in bursts. To question this threshold, we took advantage of all the behavioral events that we measured.

In our experiments, we make the assumption that vocalizations find their meaning in relation to the behavioral events of the mice. We determined this threshold by linking the behavioral events with the USVs. Therefore, the same way we computed correlations between USVs and behavioral events, we computed the same correlation between USV bursts and behavioral events (see correlation methods “USVs or Burst versus events”). In our method, we simulated all sets of bursts using different thresholds, from 0ms to 2000ms, and found how much USV bursts defined with each threshold correlate with events. The **Supplementary Table SUP_USV_Table** and **Supplementary Figure SUP_USV B** displays the evolution of the correlation with behavioral events as a function of the threshold for the Train2 event. We then extended this example to all the behavioral events (**Supplementary Figure SUP_USV C**). At zero, all vocalizations are separated, therefore bursts exactly fits the vocalizations and their correlation with the event is the lowest possible. Then, as we raise the maximum silence’s duration between USVs, bursts grow and gradually fill gaps between USVs, which makes them more correlated. The correlation always increases, but with different growth rates. Using 250ms step thresholds, we observe that between 0 and 250ms, the correlation ratio is raised by 1.2, then from 250ms to 500ms, this ratio also increases by a 1.17 factor, and between 500ms and 750ms, by 1.18. After 750ms, the ratio is lower: 1.04, 1.02, and 1.01 for 750-1000, 1000-1250, and 1250-2000, respectively. The optimal threshold is the one that maximizes the correlation while being located just before the plateau. Regarding this criteria, we set this threshold at 750ms for females B6 3mo. Rising this threshold over 750ms would not raise significantly the correlation of the burst with events. Using this method makes more significant bursts than building them with an arbitrary 250ms or 500ms threshold.

A)

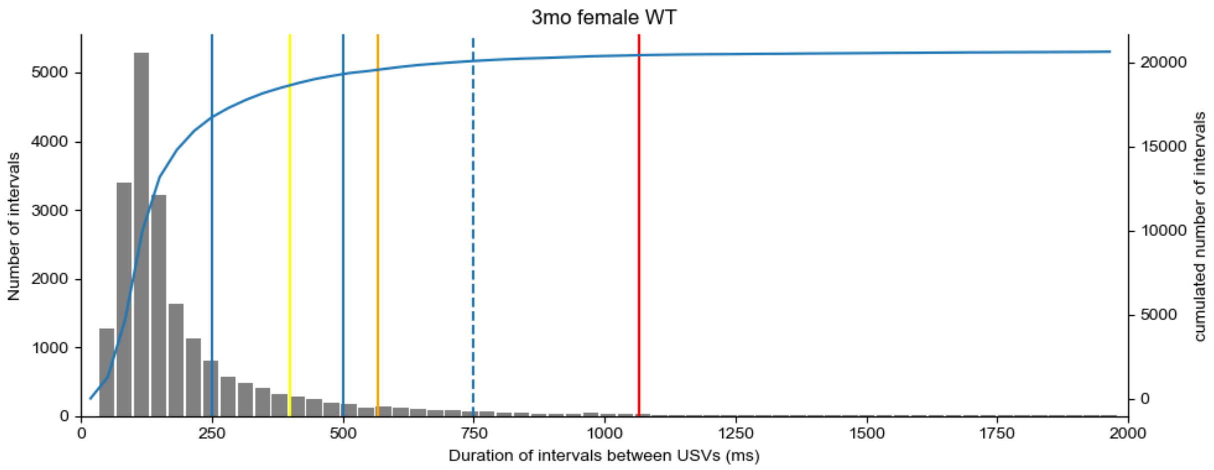

B)

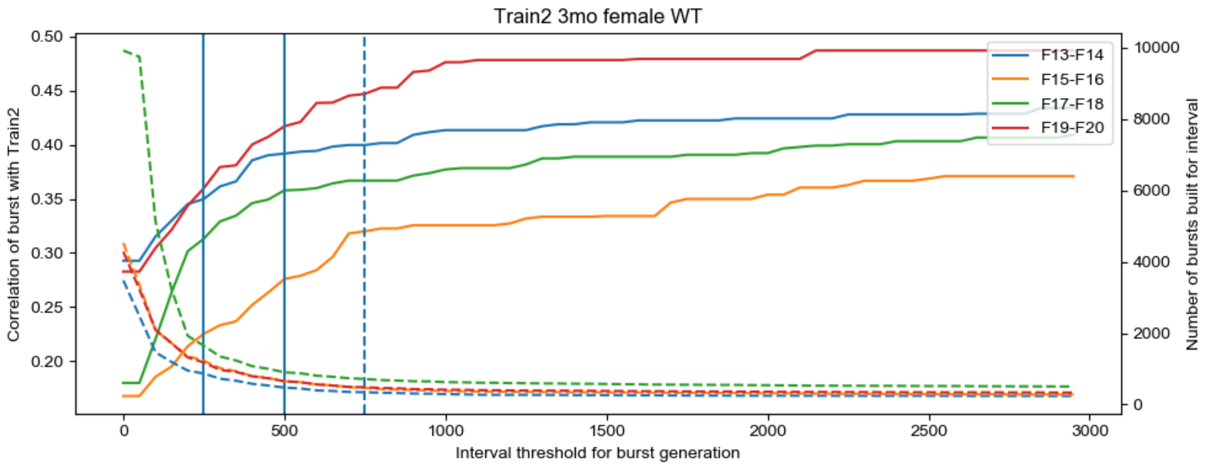

C)

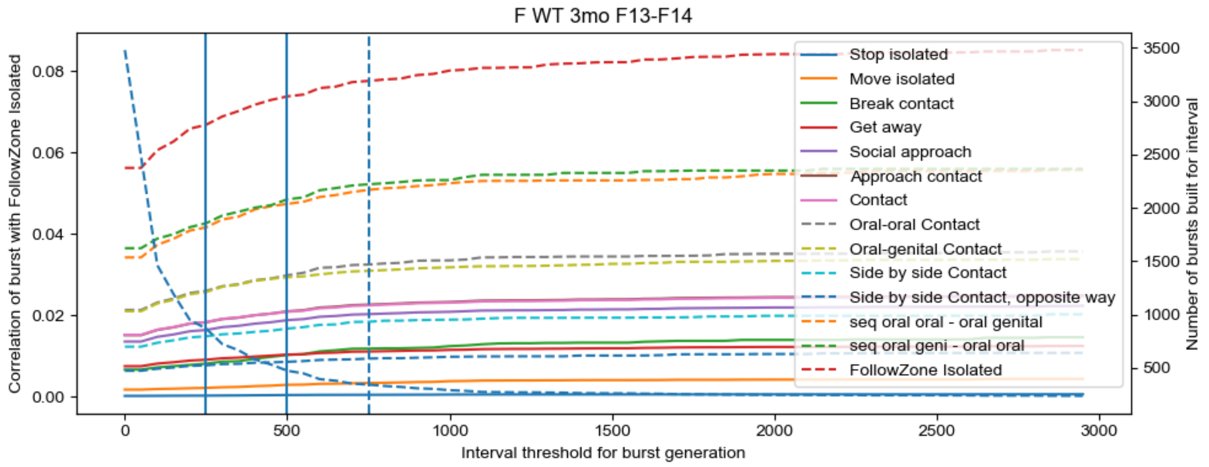

Figure SUP_USV: Interval distribution and simulation of burst as a function of the intervals for WT mice. A) Interval distribution for 3mo Female WT. Yellow, orange, red: 90%, 95%, 99% percentile of distribution. B) Simulation of burst generation as a function of the interval duration for 3mo Female WT. C) Simulation for 3mo Female WT F13-F14 of burst generation for each event.

Supplementary Table SUP_USV_Table: growing ratio based on all events. Data represented as mean +/- std.

correlations' ratio	0 - 250	250 - 500	500 - 750	750 - 1000	1000-1250	1250-1500	1500-1750	1750-2000
WT	1.21 +/- 0.02	1.17 +/- 0.09	1.18 +/- 0.03	1.04 +/- 0.03	1.02 +/- 0.01	1.01 +/- 0.005	1.01 +/- 0.008	1.01 +/- 0.005
Shank3	1.87 +/- 0.05	1.25 +/- 0.05	1.1 +/- 0.01	1.06 +/- 0.01	1.01 +/- 0.005	1.01 +/- 0.01	1.03 +/- 0.01	1.009 +/- 0.005

For *Shank3*$^{-/-}$ mice, we observed that the distribution of intervals is similar as WT (**Supplementary Figure SUP_USV_SHANK3**) and **Supplementary Table SUP_USV_Table**, but it is skewed toward short intervals.

A)

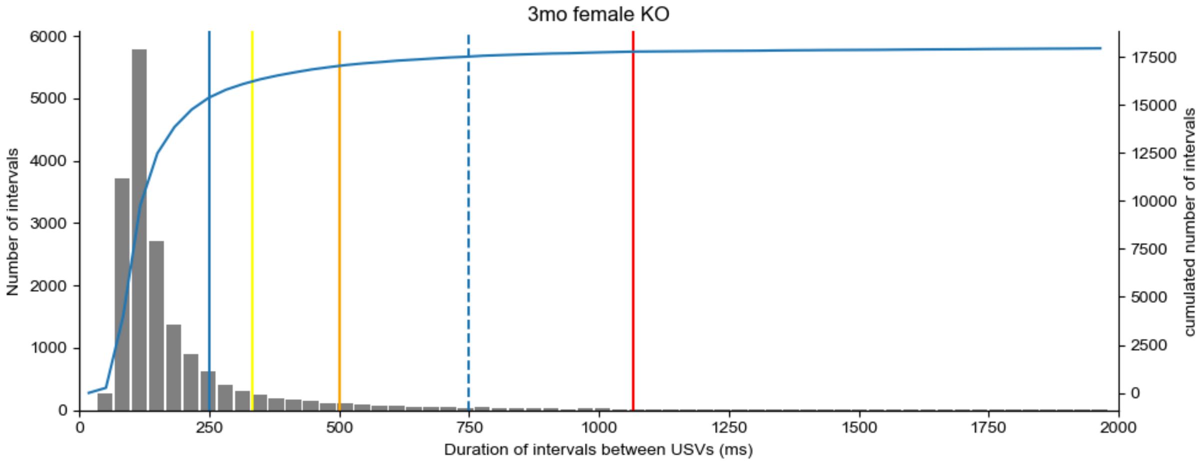

B)

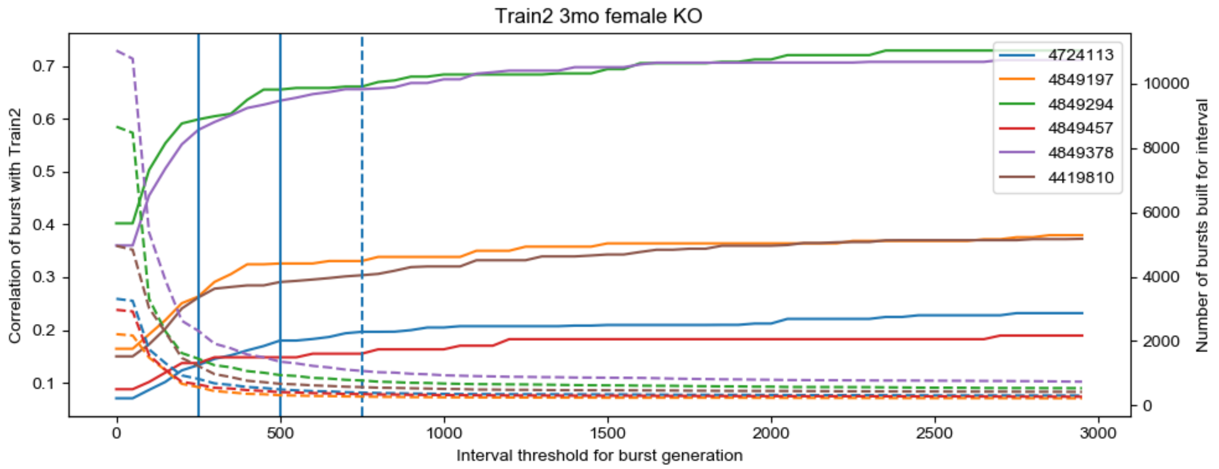

C)

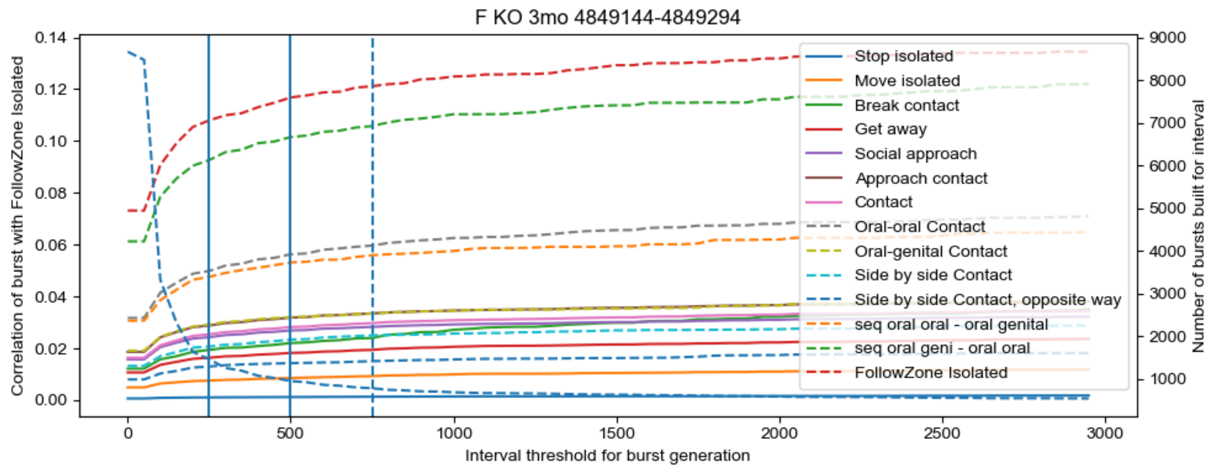

Figure SUP_USV_SHANK3: Interval distribution and simulation of burst as a function of the intervals for *Shank3*$^{-/-}$ mice. A) Interval distribution for 3mo *Shank3*$^{-/-}$ females. Yellow, orange, red: 90%, 95%, 99% percentile of distribution. B) Simulation of burst generation as a function of the interval duration for 3mo *Shank3*$^{-/-}$ females. C) Simulation for one pair of 3mo *Shank3*$^{-/-}$ females of burst generation for each event.

Distribution of intervals depending on age, sex, mutation

The distribution of intervals (**Supplementary Figure SUP_USV_INTERVALS** below) is quite similar for all different sex, train and age. Nevertheless, a few differences arise: *Shank3*$^{-/-}$ females emit less vocalizations with very low intervals than the others. 5 weeks B6 females tend to emit more vocalizations with low intervals. For males, there is not enough data to draw a conclusion or compare results.

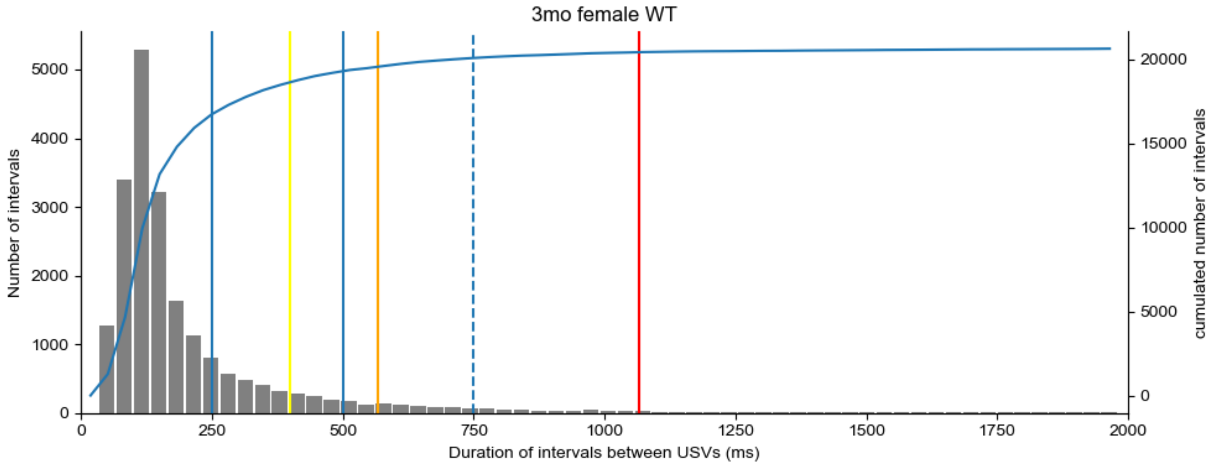
3mo female WT
Number of intervals
cumulated number of intervals
Duration of intervals between USVs (ms)

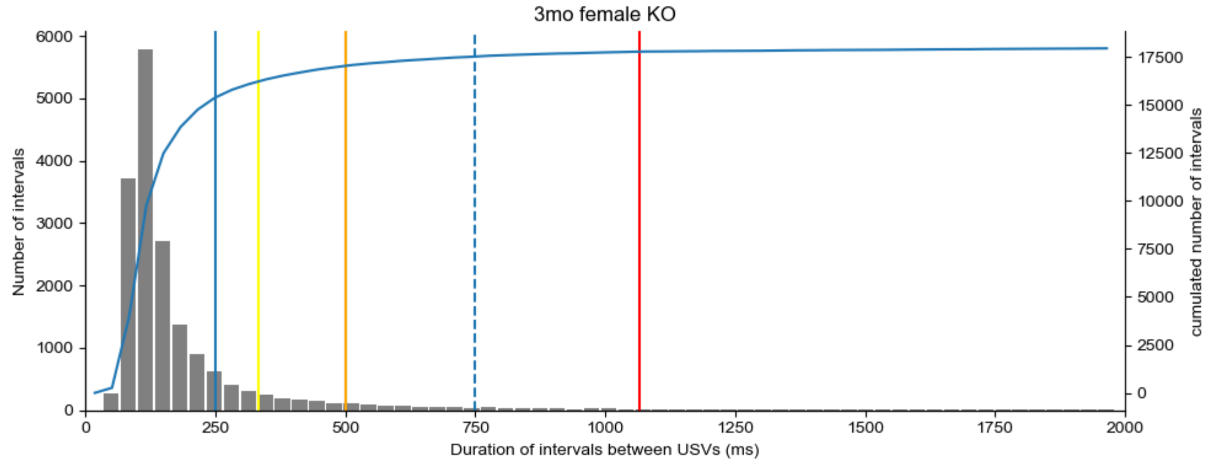
3mo female KO
Number of intervals
cumulated number of intervals
Duration of intervals between USVs (ms)

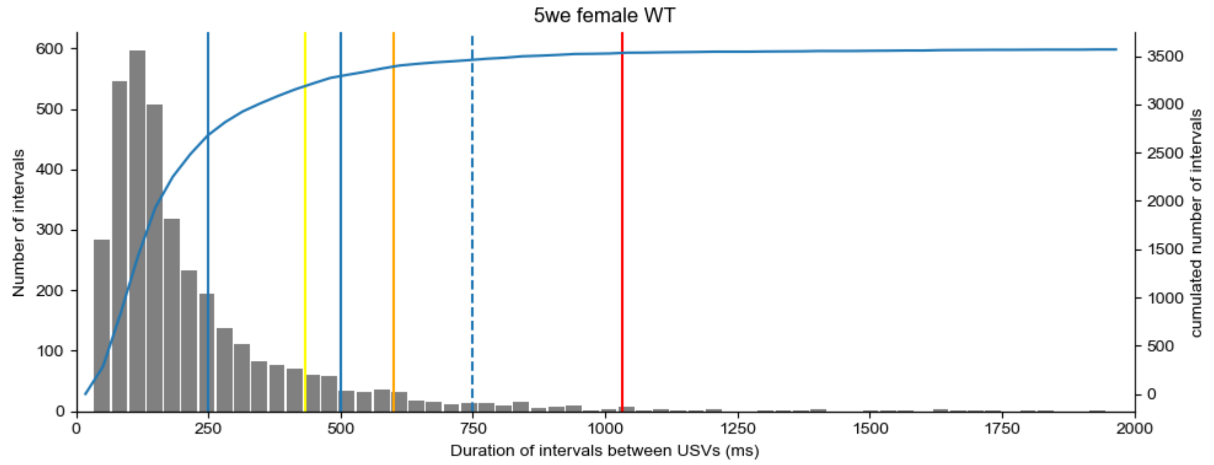
5we female WT
Number of intervals
cumulated number of intervals
Duration of intervals between USVs (ms)

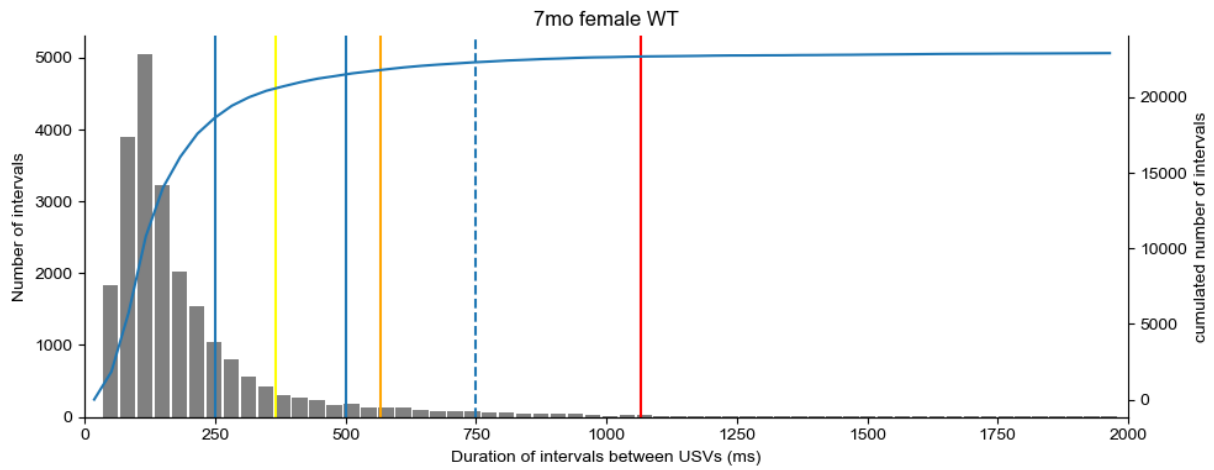
7mo female WT
Number of intervals
cumulated number of intervals
Duration of intervals between USVs (ms)

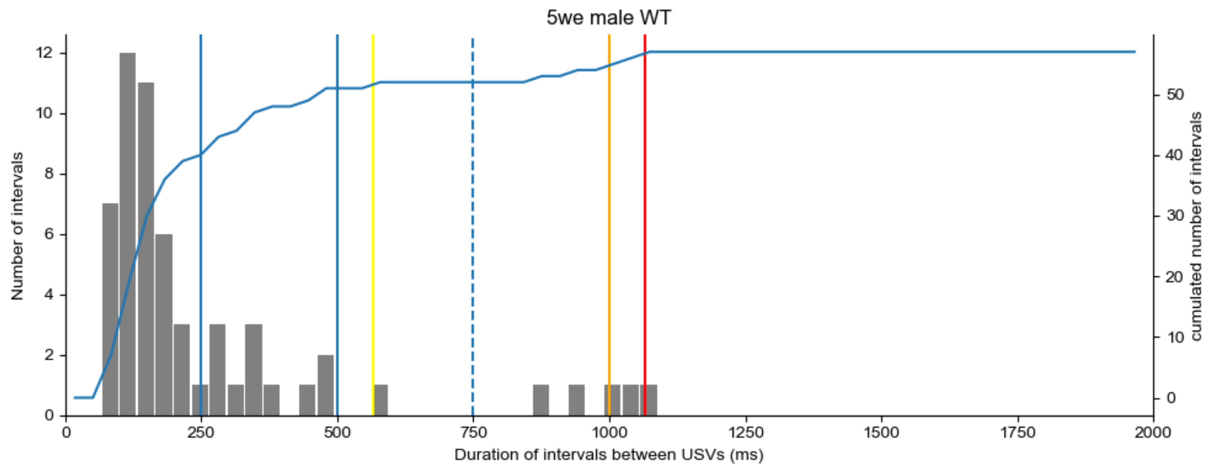
5we male WT
Number of intervals
cumulated number of intervals
Duration of intervals between USVs (ms)

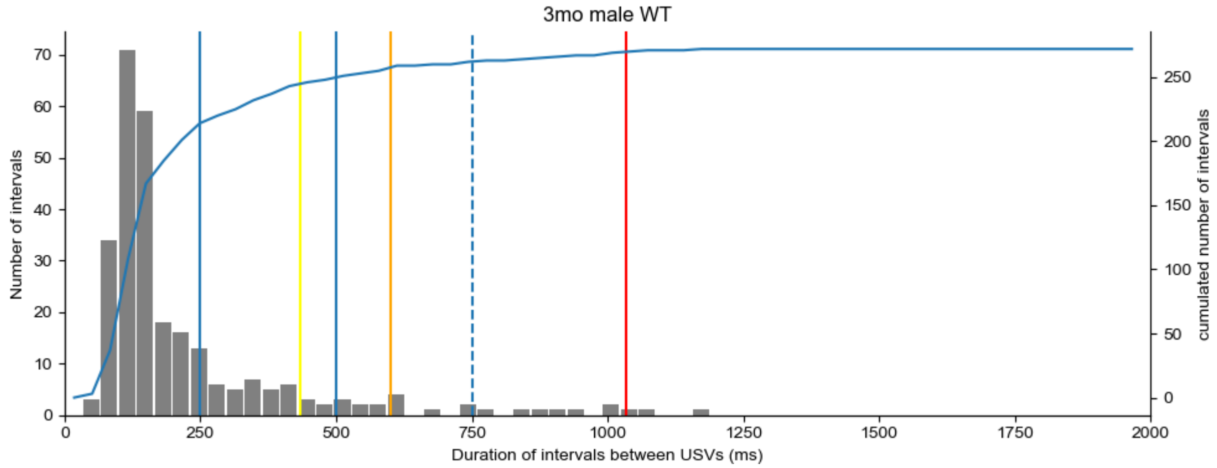
3mo male WT
Number of intervals
cumulated number of intervals
Duration of intervals between USVs (ms)

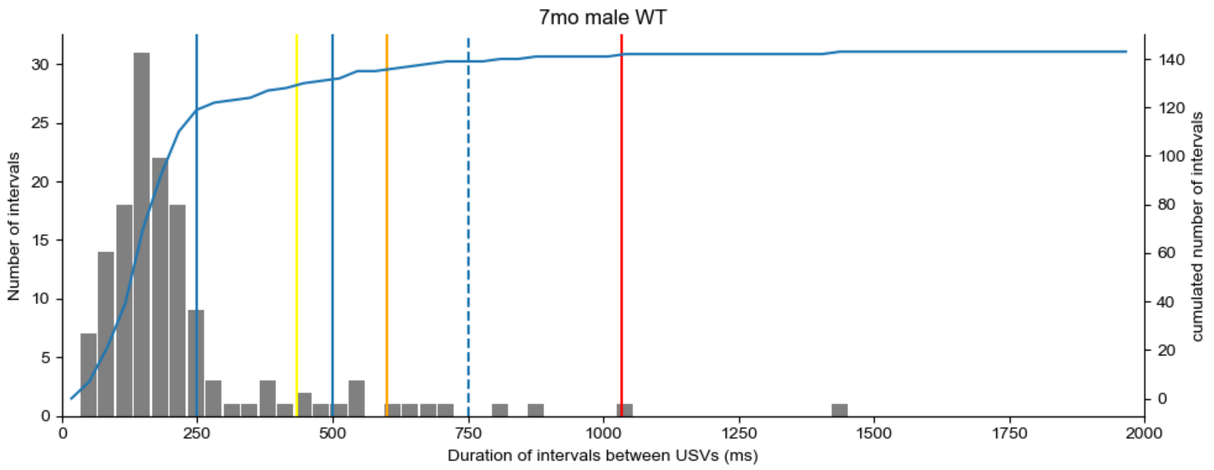

Figure SUP_USV_INTERVALS: Distribution of intervals depending on age, sex, mutation. Vertical blue bars: 250ms, 500ms, 1000ms. Yellow, orange, red: 90%, 95%, 99% percentile of distribution.

7 Vocalization are synchronized with behavioral events

To perform this analysis, we have a strong assumption that vocalizations are linked with the behaviors that Live Mouse Tracker can detect. We proved this point by randomly shifting USVs within 10 minutes around their original position. In that case, the graph shows no correlations between simulated USVs bursts and behavioral events. As we reduce the random distribution of USVs around their original position with a random factor (**Supplementary Figure BURST_SIMULATION**) of 10 minutes (A), 1 minute (B), 10 seconds (C), or no random factor (D), we observed that the curve tends to grow faster as the random factor decrease, up to no random, where the best correlation is observed.

A)

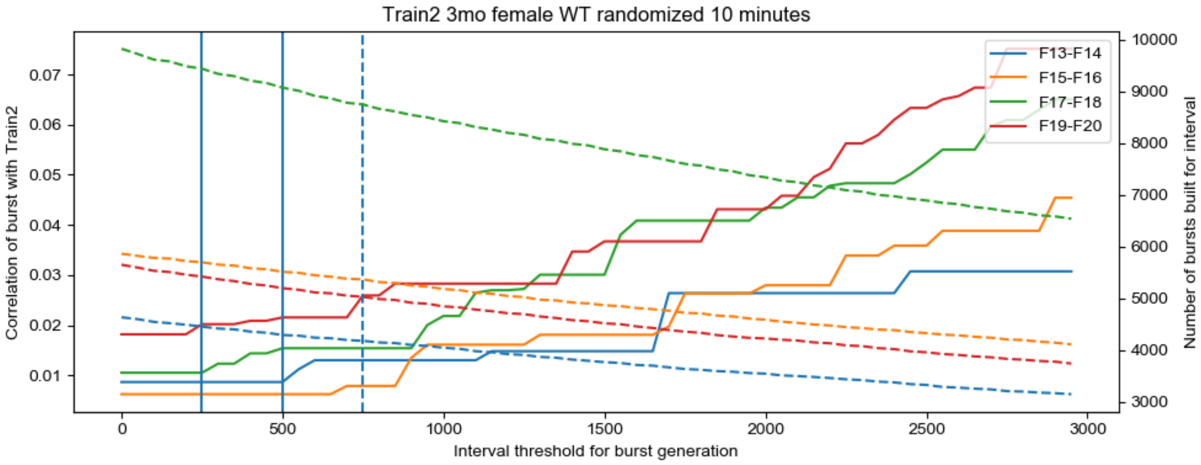
Train2 3mo female WT randomized 10 minutes
Correlation of burst with Train2
Number of bursts built for interval
Interval threshold for burst generation
F13-F14
F15-F16
F17-F18
F19-F20
0.01
0.02
0.03
0.04
0.05
0.06
0.07
3000
4000
5000
6000
7000
8000
9000
10000
0
500
1000
1500
2000
2500
3000

B)

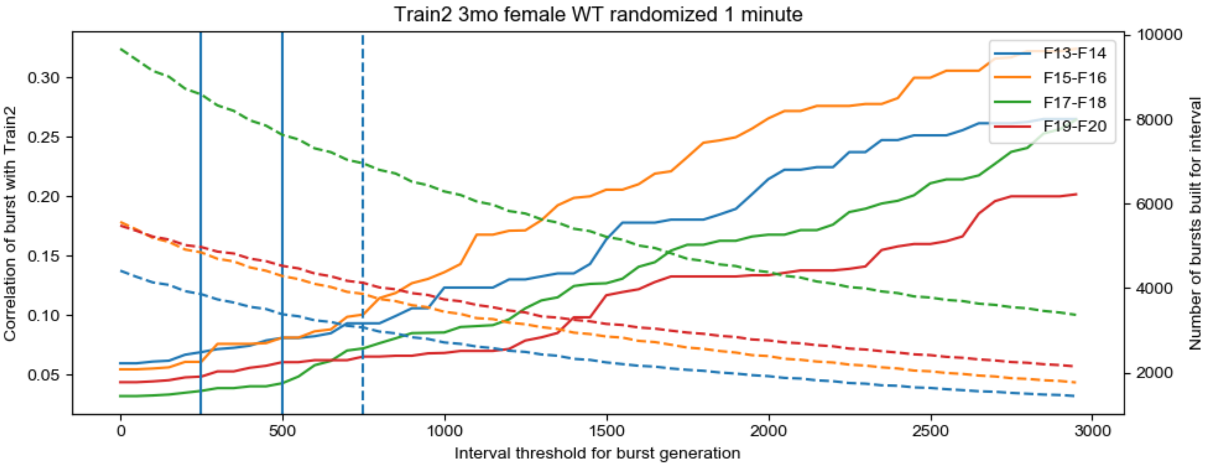
Train2 3mo female WT randomized 1 minute
Correlation of burst with Train2
Number of bursts built for interval
Interval threshold for burst generation
F13-F14
F15-F16
F17-F18
F19-F20
0.05
0.10
0.15
0.20
0.25
0.30
2000
4000
6000
8000
10000
0
500
1000
1500
2000
2500
3000

C)

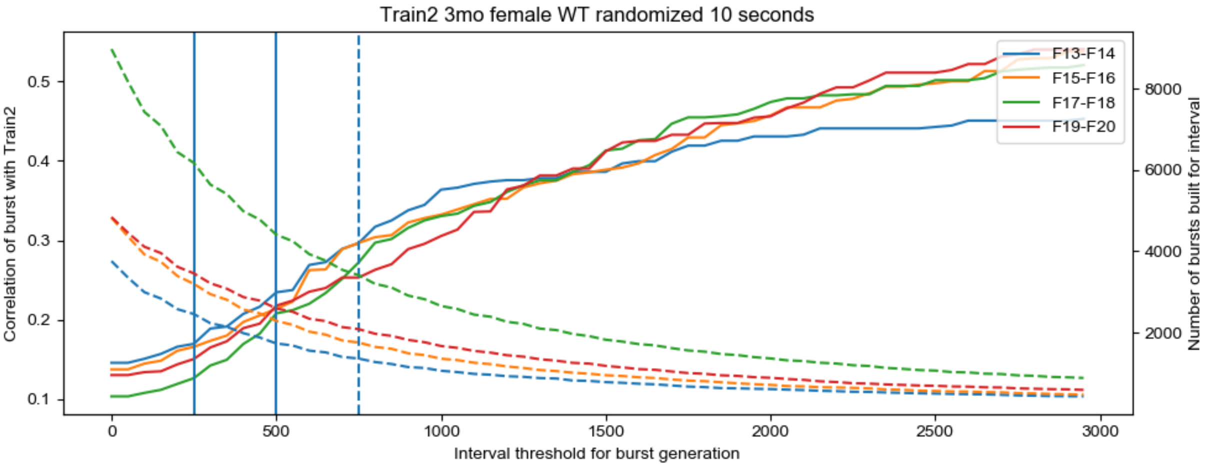
Train2 3mo female WT randomized 10 seconds
Correlation of burst with Train2
Number of bursts built for interval
Interval threshold for burst generation
F13-F14
F15-F16
F17-F18
F19-F20
0.1
0.2
0.3
0.4
0.5
2000
4000
6000
8000
0
500
1000
1500
2000
2500
3000

D)

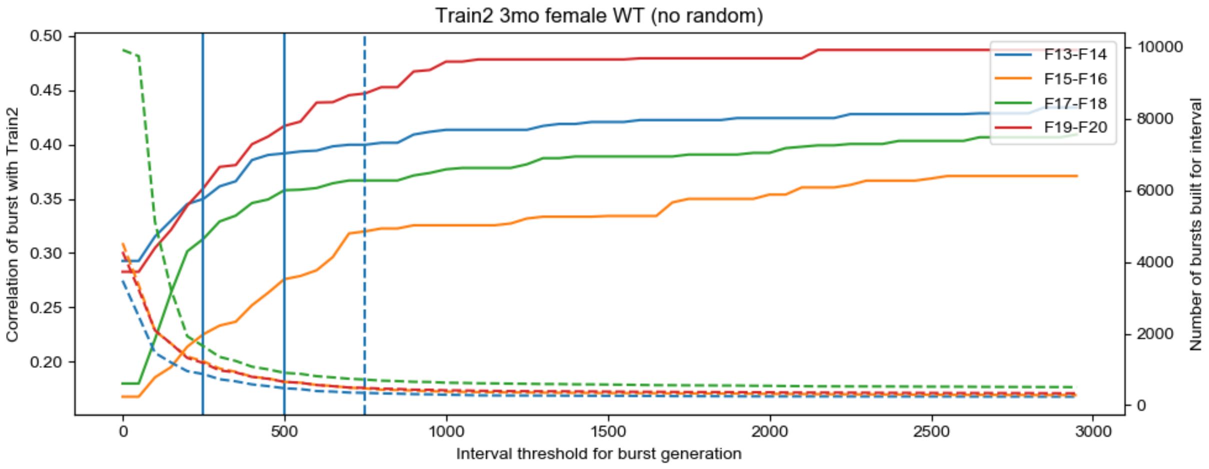

Figure BURST_SIMULATION: displays the correlation of bursts with the Train2, several simulations are displayed here using randomized dataset with a vocalization shuffling parameter of (A) 10 minutes, (B) 1 minute, (C) 10 seconds, (D) no random.

8 Supplementary References

Arriaga, G., Zhou, E. P., and Jarvis, E. D. (2012). Of mice, birds, and men: the mouse ultrasonic song system has some features similar to humans and song-learning birds. *PLoS One* 7, e46610–e46610. doi:10.1371/journal.pone.0046610.

Burkett, Z. D., Day, N. F., Peñagarikano, O., Geschwind, D. H., and White, S. A. (2015). VoICE: A semi-automated pipeline for standardizing vocal analysis across models. *Sci. Rep.* 5, 10237. doi:10.1038/srep10237.

Castellucci, G. A., Calbick, D., and McCormick, D. (2018). The temporal organization of mouse ultrasonic vocalizations. *PLoS One* 13, e0199929. Available at: https://doi.org/10.1371/journal.pone.0199929.

Chabout, J., Sarkar, A., Dunson, D. B., and Jarvis, E. D. (2015). Male mice song syntax depends on social contexts and influences female preferences. *Front. Behav. Neurosci.* 9. doi:10.3389/fnbeh.2015.00076.

Coffey, K. R., Marx, R. G., and Neumaier, J. F. (2019). DeepSqueak: a deep learning-based system for detection and analysis of ultrasonic vocalizations. *Neuropsychopharmacology* 44, 859–868. doi:10.1038/s41386-018-0303-6.

Ey, E., Torquet, N., Le Sourd, A.-M., Leblond, C. S., Boeckers, T. M., Faure, P., et al. (2013). The Autism ProSAP1/Shank2 mouse model displays quantitative and structural abnormalities in ultrasonic vocalisations. *Behav. Brain Res.* 256, 677–689. doi:10.1016/j.bbr.2013.08.031.

Hertz, S., Weiner, B., Perets, N., and London, M. (2020). Temporal structure of mouse courtship vocalizations facilitates syllable labeling. *Commun. Biol.* 3, 333. doi:10.1038/s42003-020-1053-7.

Neunuebel, J. P., Taylor, A. L., Arthur, B. J., and Egnor, S. R. (2015). Female mice ultrasonically interact with males during courtship displays. *Elife* 4. doi:10.7554/eLife.06203.

Seagraves, K. M., Arthur, B. J., and Egnor, S. E. R. (2016). Evidence for an audience effect in mice: male social partners alter the male vocal response to female cues. *J. Exp. Biol.* 219, 1437–1448. doi:10.1242/jeb.129361.

Tachibana, R. O., Kanno, K., Okabe, S., Kobayasi, K. I., and Okanoya, K. (2020). USVSEG: A robust method for segmentation of ultrasonic vocalizations in rodents. *PLoS One* 15, e0228907. Available at: https://doi.org/10.1371/journal.pone.0228907.

Torquet, N., de Chaumont, F., Faure, P., Bourgeron, T., and Ey, E. (2016). MouseTube - A database to collaboratively unravel mouse ultrasonic communication [version 1; referees: 2 approved]. *F1000Research* 5. doi:10.12688/F1000RESEARCH.9439.1.

Van Segbroeck, M., Knoll, A. T., Levitt, P., and Narayanan, S. (2017). MUPET - Mouse Ultrasonic Profile ExTraction - Signal Processing Tool for Rapid and Unsupervised Analysis of Ultrasonic Vocalizations. *Neuron* 94, 465-485.e5. doi:10.1016/j.neuron.2017.04.005.

von Merten, S., Hoier, S., Pfeifle, C., and Tautz, D. (2014). A Role for Ultrasonic Vocalisation in Social Communication and Divergence of Natural Populations of the House Mouse (Mus musculus domesticus). *PLoS One* 9, e97244. doi:10.1371/journal.pone.0097244.

Zala, S. M., Reitschmidt, D., Noll, A., Balazs, P., and Penn, D. J. (2017). Automatic mouse ultrasound detector (A-MUD): A new tool for processing rodent vocalizations. *PLoS One* 12, e0181200. Available at: https://doi.org/10.1371/journal.pone.0181200.
